# Selective C–H Iodination of (Hetero)arenes

**DOI:** 10.1021/acs.orglett.1c01530

**Published:** 2021-06-11

**Authors:** Lalita Tanwar, Jonas Börgel, Johannes Lehmann, Tobias Ritter

**Affiliations:** † Max-Planck-Institut für Kohlenforschung, Kaiser-Wilhelm-Platz 1, 45470 Mülheim an der Ruhr, Germany; ‡ Institute of Organic Chemistry, RWTH Aachen University, Landoltweg 1, 52074 Aachen, Germany

## Abstract

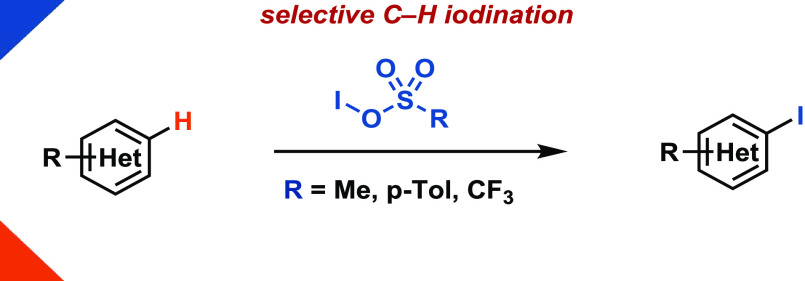

Iodoarenes are versatile intermediates and common synthetic targets in organic synthesis. Here, we present a strategy for selective C–H iodination of (hetero)arenes with a broad functional group tolerance. We demonstrate the utility and differentiation to other iodination methods of supposed sulfonyl hypoiodites for a set of carboarenes and heteroarenes.

Aromatic C–I bonds are among the most versatile synthetic handles in organic synthesis^1,2^ because they exhibit desirable reactivity, often superior to the other C–halogen bonds, such as in cross coupling reactions,^3−7^ when transformed into λ^3^-iodanes,^8^ for lithium-halogen exchange,^9^ or for the generation of aryl radicals.^10,11^ Electrophilic aromatic substitution (S_E_Ar) reactions are among the most widely used synthetic methods to install C–I bonds but typically afford mixtures of isomers.^12^ Iodination of arenes is generally more difficult to achieve than chlorination and bromination due to the limited availability of electrophilic iodination reagents that are comparable in reactivity to their chlorine and bromine counterparts. Molecular iodine (I_2_) and other electrophilic iodinating reagents such as *N*-iodosuccinimde (NIS),^13^ and 1,3-diiodo-5,5-dimethylhydantoin (DIH)^14^ are generally not sufficiently reactive to react with electron-deficient arenes and many heterocycles and, if so, commonly give mixtures of constitutional isomers.^15^ Herein, we demonstrate the discovery of a novel regioselective (hetero)arene iodination reaction by a mixture of bis(methanesulfonyl) peroxide (**1**) and iodide (Figure 1). We presumed the formation of previously unexplored sulfonyl-based hypoiodite as an electrophilic iodination reagent and subsequently designed its independent in situ formation by the synthetically more convenient addition of silver mesylate to molecular iodine to result in a previously unappreciated, practical iodination reaction that expands the scope of contemporary electrophilic aromatic iodination chemistry.

**Figure 1:**
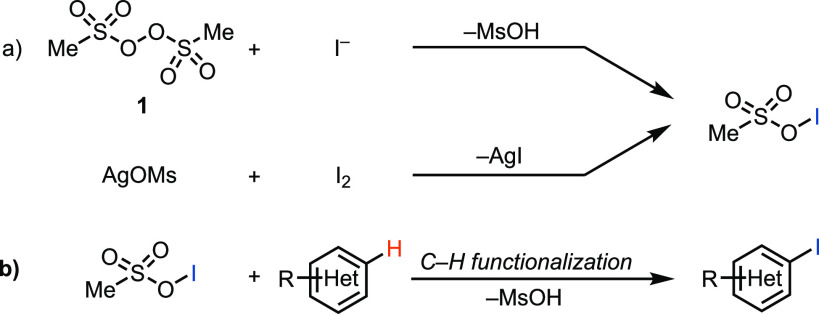
(a) Two different methods to obtain hypoiodites. (b) Aromatic C–H iodination of (hetero)arenes via sulfonyl hypoiodites.

Notwithstanding rare enzyme-catalyzed aromatic C–H iodination,^16,17^ many of the reported arene iodination methods often require strongly acidic and harsh reaction conditions such as the use of 95% H_2_SO_4_ as a solvent or reaction temperatures in excess of 120 °C, which limits the functional group tolerance and overall utility of iodination chemistry.^18−21^ Activation of molecular iodine for aromatic iodination, by modifying its electrophilicity, has been achieved by using oxidizing reagents such as Pb(OAc)_4_, or CrO_3_ dissolved in a mixture of acetic acid with acetic anhydride,^22^ which can result in overiodination of electron-rich arenes. Olah and co-workers reported a C–H iodination of deactivated arenes with NIS in neat TfOH^23^ and BF_3_–H_2_O.^24^ In 2018, the Crousse group reported halogenation of (hetero)arenes in HFIP that is limited to electron-rich substrates.^25^ Furthermore, the Nagib group reported the site-selective incorporation of various anions including Cl^–^, Br^–^, OMs^–^, OTs^–^, and OTf^–^ to heteroarenes via an iodane intermediate; however, the incorporation of iodide was not shown.^26^ Moreover, the iodination of simple arenes such as toluene and benzene has been reported by using AgOTf/I_2_, Ag_2_SO_4_/I_2_, and AgNO_2_/I_2_.^27−31^ In 2011, the Lehmler group reported the iodination of chlorinated arenes using Ag_2_SO_4_/I_2_, AgSbF_6_/I_2_, AgBF_4_/I_2_, and AgPF_6_/I_2_, which introduce the iodine in the *para* position to the Cl-substituent.^32^ In addition, significant progress has been made to enhance the reactivity of NIS by using Brønsted and Lewis acids as well as Lewis base catalysts; however, such methods have only been shown to perform on relatively simple arenes, such as anisole.^33^ Iodination of more complex small molecules has not been described with any of the methods described above. Hence, there is still a demand for developing mild and effective methods for selective C–H iodination of complex arenes. Herein, we methodically explore the regioselective aromatic C–H iodination of complex (hetero)arenes, with a special emphasis on the use of Ag(I) sulfonates. Sulfonates could react with iodine to sulfonyl hypoiodites that are not accessible in reactions with other silver salts exhibiting counterions, which had been evaluated before, such as BF_4_ or SbF_6_. The reactivity profile of the putative sulfonyl hypoiodites is adaptable through the appropriate choice of the silver salt and enlarges the currently available scope for (hetero)aromatic iodination chemistry.

Based on our reaction chemistry developed with **1**,^34^ we have discovered a productive, high-yielding iodination reaction in the presence of iodide and **1** (Table 1). Because **1** is explosive, we attempted to reproduce the observed reactivity with reagents that are more convenient and safer. We assumed the formation of methanesulfonylhypoiodite as the reactive electrophilic iodinating reagent that formed in situ upon mixing **1** and iodide and attempted to intercept it independently through the reaction of molecular iodine with silver mesylate. We successfully observed a similar reactivity, which is superior when compared to conventional iodination reagents and reactions (Table 1). Because the putative sulfonyl hypoiodites are prepared in situ in solution, this reaction setup does not share the same safety concerns associated with the explosiveness of bis(methansulfonyl) peroxide that was used as an isolated solid.

**Table 1:**
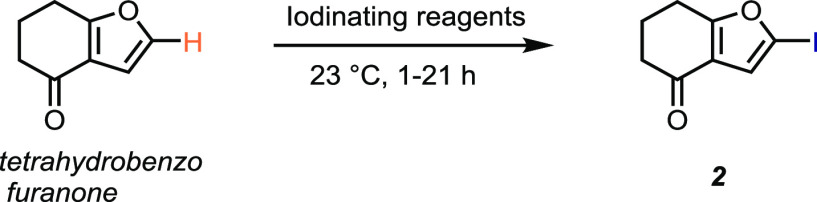
Comparison of Sulfonyl Hypoiodites with Other Known Electrophilic Iodinating Methods^a^

comparison with electrophilic iodinating methods	yield^b^
(MsO)_2_ (**1**, 1.8 equiv) + TBAI (2.0 equiv) in 0.2 M MeCN	84%
I_2_ (1.3 equiv) + AgOMs (1.3 equiv) in 0.2 M MeCN	90%
I_2_ (1 equiv) + AgOTf (1 equiv) in 0.2 M DCM	18%
NIS (1 equiv) in 0.2 M HFIP	54%
NIS (10 equiv) in 0.2 M TfOH	0%
Ph_2_S_2_ (5 mol %) + DIH (0.75 equiv) in 0.3 M MeCN	29%
I_2_ (1 equiv) + AgPF_6_ (1 equiv) in 0.2 M DCM	16%

a Reactions were carried out on a 0.1 mmol scale.

b Yields determined by ^1^H NMR spectroscopy with dibromomethane as an internal standard.

Although NIS is a practical and convenient reagent for the iodination of simple, electron-rich (hetero)arenes, its utility is severely limited for less electron-rich substrates. While NIS can furnish the same iodinated product **2** (Table 1), albeit in a substantially lower yield, for more complex, functionalized, or electron-poor substrates, it often fails, as shown in Table 2, and for a selection of a dozen compounds in the Supporting Information, Table S1.

**Table 2:**
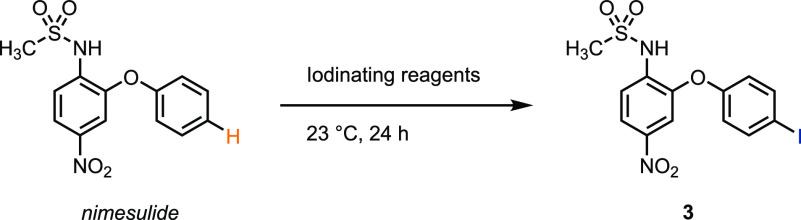
C–H Iodination of Nimesulide^a^

electrophilic C–H iodination method	yield^b^
I_2_ (2.0 equiv) + AgOMs (2.0 equiv) in 0.2 M MeCN	92%^c^
NIS (1 equiv) in 0.2 M HFIP	<1%

a Reactions were carried out on a 0.1 mmol scale.

b Yields determined by ^1^H NMR spectroscopy with dibromomethane as an internal standard.

c Isolated yield.

The simple reaction setup of mixing a silver salt that could form a putative iodine–oxygen bond potentially enables the in situ generation of a variety of hypoiodites that could, in the best case, be adapted to the required reactivity for efficient iodination of a given arene. In other words, tuning the reactivity of the presumed hypoiodite would allow for an appropriate electrophilicity for any given (hetero)arene.

After a brief evaluation of simple arenes (Table 3), we focused our attention on the C–H iodination of various heteroarenes because N-containing heterocycles represent an important class of compounds in medicinal chemistry.^35^ A variety of functional groups such as electron-rich pyridines, carboxylic acids, esters, amines, sulfonamides, and phthalimides are well tolerated. If acid-sensitive functional groups are present, the addition of Li_2_CO_3_ as a base to neutralize the in situ formed acid byproduct results in productive iodination. The iodination reaction reported here could be extended to electron-rich heteroarenes such as *N*-methylpyrrole (**10**) and 2,6–dimethoxypyridine (**11**), with the best results obtained when using silver acetate. Compounds containing ketones are generally challenging for iodination; however, ketone **2** was obtained in 80% isolated yield with less than 5% α-iodination byproduct. Other 5-membered heteroarenes such as thiazole (**14**) and pyrroles (**17**) afforded the highest yields with silver tosylate.

**Table 3:**
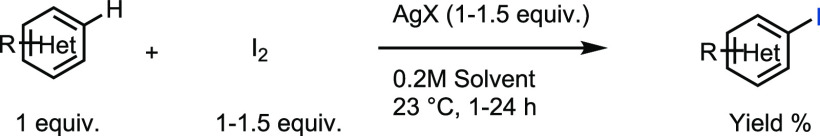

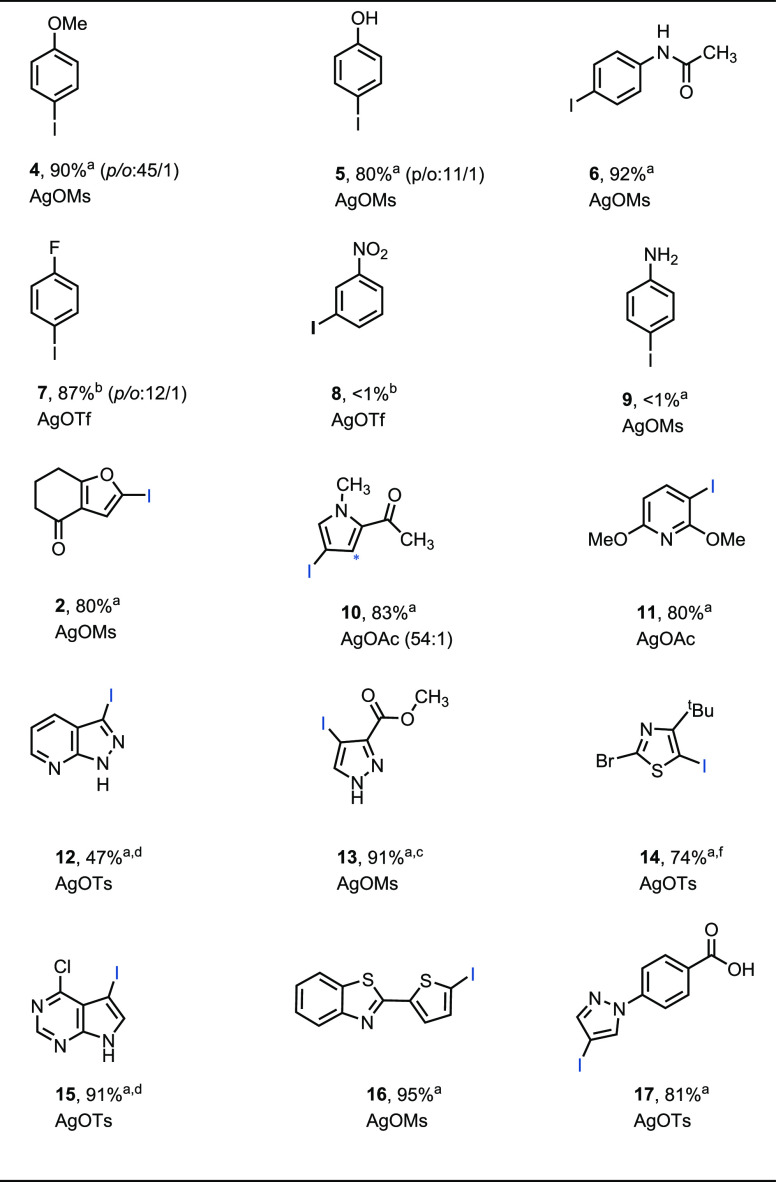
C–H Iodination of Various (Hetero)arenes^g^

a Reaction was conducted in 0.2 M MeCN.

b Reaction was conducted in 0.2 M DCM.

c I_2_ (1.3 equiv) and AgX (1.3 equiv).

d Li_2_CO_3_ (1.0 equiv) was used.

e I_2_ (1.2 equiv) and AgOTf (1.2 equiv).

f I_2_ (1.5 equiv) and AgOTs (1.5 equiv).

g General conditions except where otherwise noted: arene (0.2 mmol), AgX (0.2 mmol, 1.0 equiv), I_2_ (0.2 mmol, 1.0 equiv), 23 °C.

As can be seen in Table 4, the scope of the new iodination reaction includes a range of small-molecule pharmaceutical carboarenes. The reaction condition proved to be compatible with structurally complex arenes, such as nimesulide (**3**), procymidone (**20**), boscalid (**21**), and strychnine (**25**). Notably, no competing addition of iodine to double bonds was observed for arenes **18**, **19**, and **25**. The method often affords a high yield and high positional selectivity. A detailed study of the hypothesis that the magnitude of the selectivity can be rationalized by a charge transfer complex between hypoiodite and arene as we observed in the related mesyloxylation reaction^34^ was prevented by in situ formation of the reactive intermediate. Scale-up to the gram scale was established for iodination of coumarin1 with silver methanesufonate to afford product **19** in 91% yield. Electron-withdrawing arenes gave low yields for the corresponding iodinated products.

**Table 4:**
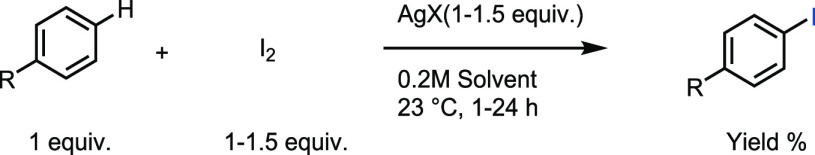

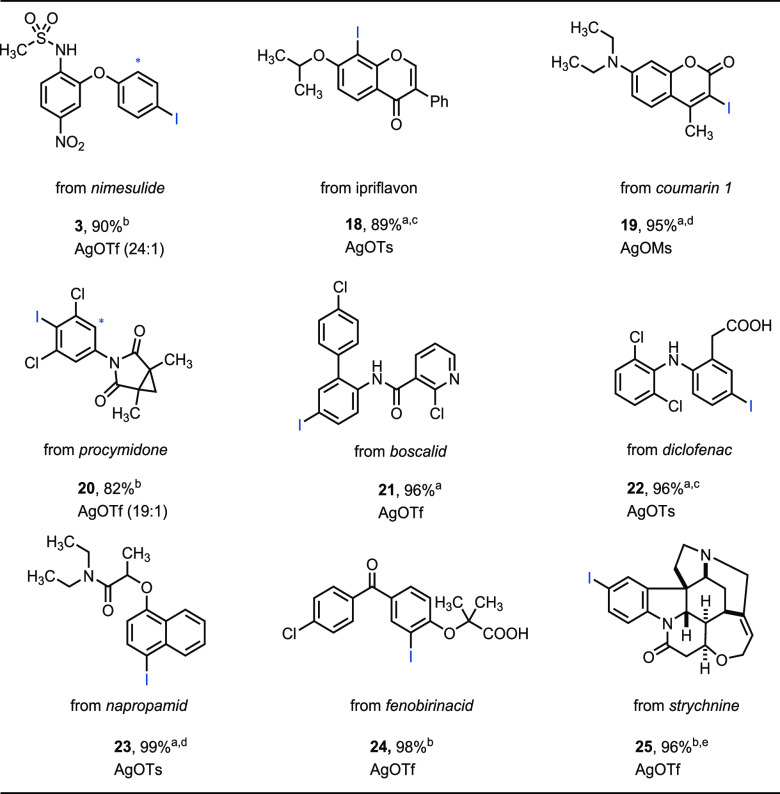
C–H Iodination of Small-molecule Drugs^g^

a Reaction was conducted in 0.2 M MeCN.

b Reaction was conducted in 0.2 M DCM.

c I_2_ (1.3 equiv) and AgX (1.3 equiv).

d Li_2_CO_3_ (1.0 equiv) was used.

e I_2_ (1.2 equiv) and AgOTf (1.2 equiv).

f I_2_ (1.5 equiv) and AgOTs (1.5 equiv).

g General conditions except where otherwise noted: arene (0.2 mmol), AgX (0.2 mmol, 1.0 equiv), I_2_ (0.2 mmol, 1.0 equiv), 23 °C.

We observed chemoselective iodination for sp^2^ C–H functionalization with no benzylic or α-carbonyl oxidation observed, an advantage when compared to the combination of iodine and other oxidants.^36^ The reaction is insensitive to oxygen or traces of water and thus can be carried out under an ambient atmosphere. For most substrates, clean conversion of the starting material to the product was observed, which renders purification straightforward. When compared to conventional iodinating reagents such as NIS, the reaction conditions shown here typically afforded substantially higher yields, higher selectivity, and no overiodination (see Table S1 in the Supporting Information for a comparison).

In summary, we have presented a simple C–H iodination of various carboarenes and heteroarenes via putative sulfonyl hypoiodites that has not been appreciated before and extends the substrate scope of iodination chemistry. The operational ease, scalability, broad functional group tolerance, and substrate scope make this protocol suitable for both academic and industrial settings.

## Supporting Information Available

Detailed experimental procedures and spectroscopic characterization (PDF)
