# Supplementary material for: Selective C–H Iodination of (Hetero)arenes
Source: Org Lett. 2021 Jun 11;23(13):5024–7. doi: 10.1021/acs.orglett.1c01530 (PMC8289285; doi:10.1021/acs.orglett.1c01530)
Supplement: Supplementary file 1 — ol1c01530_si_001.pdf [file ol1c01530_si_001.pdf]

SUPPORTING INFORMATION

Selective C–H iodination of (hetero)arenes

Lalita Tanwar,[†,‡] Jonas Börgel,[†] Johannes Lehmann,[†] Tobias Ritter*[,†,‡]

[†]Max-Planck-Institut für Kohlenforschung, Kaiser-Wilhelm-Platz 1, D-45470 Mülheim an der Ruhr, Germany

[‡]Institute of Organic Chemistry, RWTH Aachen University, Landoltweg 1, 52074, Aachen, Germany

*Correspondence to: ritter@kofo.mpg.de

TABLE OF CONTENTS

TABLE OF CONTENTS 1

MATERIALS AND METHODS 4

EXPERIMENTAL DATA 5

General procedure for C–H iodination of (hetero)arenes 5

2-Iodo-tetrahydrobenzofuranone (**2**) 5

Iodinated-nimesulide (**3**) 6

4-Iodoanisole (**4**) 6

4-Iodophenol (**5**) 8

4-Iodoacetanilide (**6**) 8

4-Iodofluorobenzene (**7**) 9

4-Iodonitrobenzene (**8**) 9

4-Iodoaniline (**9**) 10

2-Acetyl-4-iodo-1-methylpyrrole (**10**) 11

3-Iodo-2,6-dimethoxypyridine (**11**) 12

3-Iodoazaindazole (**12**) 12

4-Iodopyrazole-3-carboxylate (**13**) 13

2-Iodo-5-bromothiazole (**14**) 14

6-Chloro-7-iododezapurine (**15**) 14

2-(5-Iodothiophene)-benzothiazole (**16**) 15

4-Iodopyrazole-1-benzoic acid (**17**) 15

Iodinated-ipriflavone (**18**) 16

Iodinated-coumarin1 (**19**) 17

Gram-scale synthesis: Iodinated-coumarin1 (**19**) 17

Iodinated-procymidone (**20**) 18

Iodinated-boscalid triflate salt (**21**) 19

Iodinated-diclofenac (**22**) 19

Iodinated-napropamid (**23**) 20

Iodinated-fenobirinacid (**24**) 21

Iodinated-strychnine triflate salt (**25**) 22

Formation of acetyl hypoiodite 22

Comparison to NIS method 23

Table S1. Comparison of hypoiodites method with NIS 24

Electrochemical data 24

Cyclic Voltammograms 24

Table S2. Value of E^{red} vs Ag/AgCl in MeCN. 25
Table S3. Modulation of sulfonyl based hypoiodites. 26

SPECTROSCOPIC DATA 27
^{1}H NMR of 2-Iodo-tetrahydrobenzofuranone (**2**) 27
^{13}C NMR of 2-Iodo-tetrahydrobenzofuranone (**2**) 28
^{1}H NMR of Iodinated-nimesulide (**3**) 29
^{13}C NMR of Iodinated-nimesulide (**3**) 30
^{1}H NMR of 4-Iodoanisole (**4**) 31
^{13}C NMR of 4-Iodoanisole (**4**) 32
^{1}H NMR of 4-Iodophenol (**5**) 33
^{13}C NMR of 4-Iodophenol (**5**) 34
^{1}H NMR of 4-Iodoacetanilide (**6**) 35
^{13}C NMR of 4-Iodoacetanilide (**6**) 36
^{1}H NMR of 4-Iodofluorobenzene (**7**) 37
^{19}F NMR of 4-Iodofluorobenzene (**7**) 38
^{13}C NMR of 4-Iodofluorobenzene (**7**) 39
^{1}H NMR of 2-Acetyl-4-iodo-1-methylpyrrole (**10**) 40
^{13}C NMR of 2-Acetyl-4-iodo-1-methylpyrrole (**10**) 41
^{1}H NMR of 3-Iodo-2,6-dimethoxypyridine (**11**) 42
^{13}C NMR of 3-Iodo-2,6-dimethoxypyridine (**11**) 43
^{1}H NMR of 3-Iodoazaindazole (**12**) 44
^{13}C NMR of 3-Iodoazaindazole (**12**) 45
^{1}H NMR of 4-Iodopyrazole-3-carboxylate (**13**) 46
^{13}C NMR of 4-Iodopyrazole-3-carboxylate (**13**) 47
^{1}H NMR of 2-Iodo-5-bromothiazole (**14**) 48
^{13}C NMR of 2-Iodo-5-bromothiazole (**14**) 49
^{1}H NMR of 6-Chloro-7-iododezapurine (**15**) 50
^{13}C NMR of 6-Chloro-7-iododezapurine (**15**) 51
^{1}H NMR of 2-(5-Iodothiophene)-benzothiazole (**16**) 52

^{13}C NMR of 2-(5-Iodothiophene)-benzothiazole (**16**) 53
^{1}H NMR of 4-Iodopyrazol-1-benzoic acid (**17**) 54
^{13}C NMR of 4-Iodopyrazol-1-benzoic acid (**17**) 55
^{1}H NMR of Iodinated-ipriflavone (**18**) 56
^{13}C NMR of Iodinated-ipriflavone (**18**) 57
^{1}H NMR of Iodinated-coumarin1 (**19**) 58
^{13}C NMR of Iodinated-coumarin1 (**19**) 59
^{1}H NMR of Iodinated-procymidone (**20**) 60
^{13}C NMR of Iodinated-procymidone (**20**) 61
^{1}H NMR of Iodinated-boscalid triflate salt (**21**) 62
^{13}C NMR of Iodinated-boscalid triflate salt (**21**) 63
^{19}F NMR of Iodinated-boscalid triflate salt (**21**) 64
^{1}H NMR of Iodinated-diclofenac (**22**) 65
^{13}C NMR of Iodinated-diclofenac (**22**) 66
^{1}H NMR of Iodinated-napropamid (**23**) 67
^{13}C NMR of Iodinated-napropamid (**23**) 68
^{1}H NMR of Iodinated-fenobirinacid (**24**) 69
^{13}C NMR of Iodinated-fenobirinacid (**24**) 70
^{1}H NMR of Iodinated-strychnine triflate salt (**25**) 71
^{13}C NMR of Iodinated-strychnine triflate salt (**25**) 72
^{19}F NMR of Iodinated-strychnine triflate salt (**25**) 73

REFERENCES 74

MATERIALS AND METHODS

All reactions were carried out under an ambient atmosphere unless otherwise stated and monitored by thin-layer chromatography (TLC). Concentration under reduced pressure was performed by rotary evaporation at 25–40 °C at an appropriate pressure. Purified compounds were further dried under high vacuum (0.008–0.5 Torr). Yields refer to purified and spectroscopically pure compounds.

Solvents

Dichloromethane was purchased from Sigma-Aldrich and used as received. Acetonitrile was purchased from fisher scientific and used as received. All deuterated solvents were purchased from Eur*iso*-Top.

Chromatography

Thin layer chromatography (TLC) was performed using EMD TLC plates pre-coated with 250 μm thickness silica gel 60 F_{254} plates and visualized by fluorescence quenching under UV light. Flash chromatography was performed using silica gel (40–63 μm particle size) purchased from Geduran®. Preparatory high-performance liquid chromatographic separation was executed on a Shimadzu Prominence Preparative HPLC system.

Spectroscopy and Instruments

NMR spectra were recorded on a Bruker Ascend™ 500 spectrometer operating at 500 MHz, 125 MHz, and 470 MHz for ^{1}H, ^{13}C, and ^{19}F acquisitions, respectively; or on a Varian Unity/Inova 600 spectrometer operating at 600 MHz, 150 MHz, and 565 MHz for ^{1}H, ^{13}C, and ^{19}F acquisitions, respectively. Chemical shifts are reported in ppm with the solvent resonance as the internal standard. For ^{1}H NMR: $CDCl_3$, δ 7.26; $(CD_3)_2SO$, δ 2.50. For ^{13}C NMR: $CDCl_3$, δ 77.16; $(CD_3)_2SO$, δ 39.52.[1] Data is reported as follows: s = singlet, d = doublet, t = triplet, q = quartet, m = multiplet, br = broad; coupling constants in Hz; integration.

Starting materials

All substrates were used as received from commercial suppliers, unless otherwise stated.

EXPERIMENTAL DATA

General procedure for C–H iodination of (hetero)arenes

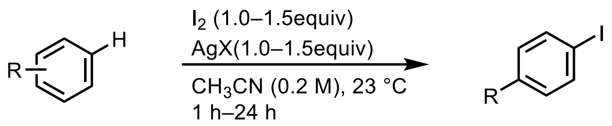

Under an ambient atmosphere, a 4 mL borosilicate vial equipped with magnetic stir bar was charged with arene (0.200 mmol, 1.00 equiv), molecular iodine (50.8 mg, 0.200 mmol, 1.00 equiv), Ag (I) salt (0.200 mmol, 1.00 equiv), and MeCN (1.0 mL, c = 0.20 M). Subsequently, the vial was capped, and the reaction mixture was stirred at 23 °C for 24 h. The reaction mixture was diluted with EtOAc (5 mL), and the resulting mixture was filtered through a short pad of silica gel using EtOAc (5 mL) as eluent. The filtrate was collected and concentrated by rotary evaporation. The residue was purified by column chromatography on silica gel to afford iodinated product.

2-Iodo-tetrahydrobenzofuranone (2)

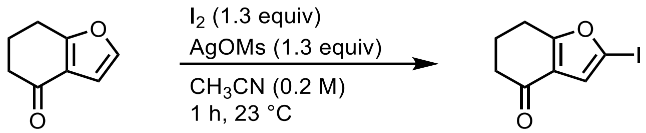

S2 **2,** 80%

Under an ambient atmosphere, a 4 mL borosilicate vial equipped with magnetic stir bar was charged with molecular iodine (66.0 mg, 0.260 mmol, 1.30 equiv), AgOMs (52.0 mg, 0.260 mmol, 1.30 equiv), tetrahydrobenzofuranone (**S2**) (27.2 mg, 0.200 mmol, 1.00 equiv), and MeCN (1.0 mL, c = 0.20 M). The vial was capped, and the reaction mixture was stirred at 23 °C for 1 h. The reaction mixture was diluted with EtOAc (5 mL), and the resulting mixture was filtered through a short pad of silica gel using EtOAc (5 mL) as eluent. The filtrate was collected and concentrated by rotary evaporation. The residue was purified by column chromatography on silica gel eluting with a solvent mixture of ethyl acetate:pentane (5:95 (v:v)) to afford 42 mg of **2** as a colorless solid (80% yield).

R_f (ethyl acetate:pentane, 10:90 (v:v)) = 0.21.

NMR Spectroscopy:

^{1}H NMR (500 MHz, $CDCl_3$, 25 ºC, δ): 6.82 (s, 1H), 2.90 (t, *J* = 6.3 Hz, 2H), 2.49 – 2.44 (m, 2H), 2.16 (p, *J* = 6.4 Hz, 2H).

^{13}C NMR (126 MHz, $CDCl_3$, 25 ºC, δ): 193.0, 171.0, 124.1, 117.1, 89.2, 37.6, 23.5, 22.6.

HRMS-EI-Ion trap (m/z) calc'd for $C_8H_7O_2I$ $[M]^+$, 261.9489; found, 261.9485; deviation: –1.5 ppm.

Iodinated-nimesulide (3)

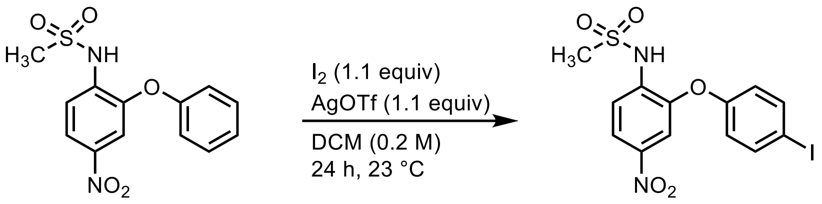

S3 **3,** 90%

Under an ambient atmosphere, a 4 mL borosilicate vial equipped with magnetic stir bar was charged with molecular iodine (55.0 mg, 0.220 mmol, 1.10 equiv), AgOTf (56.0 mg, 0.220 mmol, 1.10 equiv), nimesulide (**S3**) (61.70 mg, 0.200 mmol, 1.00 equiv), and DCM (1.0 mL, c = 0.20 M). The vial was capped, and the reaction mixture was stirred at 23 °C for 24 h. The reaction mixture was diluted with EtOAc (5 mL), and the resulting mixture was filtered through a short pad of silica gel using EtOAc (5 mL) as eluent. The filtrate was collected and concentrated by rotary evaporation. The residue was purified by column chromatography on silica gel eluting with a solvent mixture of ethyl acetate:pentane (30:70 (v:v)) to afford 90 mg of **3** as a pale yellow solid. Analytically pure sample was obtained by purification with high performance liquid chromatography with an YMC-Pack Triart C18 column with an eluent mixture of 0.1% (v/v) aqueous trifluoroacetic acid /acetonitrile (30:70 (v/v) at a flow rate of 42.5 mL·min^{-1} and UV detection at a wavelength of 220 nm to afford 78 mg of **3** as a pale yellow solid (90% yield).

R*f* (ethyl acetate:pentane, 30:70 (v:v)) = 0.35.

NMR Spectroscopy

^{1}H NMR (500 MHz, DMSO, 25 ºC, δ): 10.19 (s, 1H), 8.06 (dd, *J* = 9.1, 2.6 Hz, 1H), 7.78 (d, *J* = 8.8 Hz, 2H), 7.74 (d, *J* = 9.1 Hz, 1H), 7.63 (d, *J* = 2.6 Hz, 1H), 6.97 (d, *J* = 8.8 Hz, 2H), 3.18 (s, 3H).

^{13}C NMR (126 MHz, DMSO, 25 ºC, δ): 155.4, 146.5, 143.1, 138.8, 135.9, 121.5, 121.0, 119.9, 113.5, 88.4, 40.8.

HRMS-ESIneg- Ion trap (m/z) calc’d for $C_{13}H_{10}N_2IO_5S^-$ [M–H]$^-$, 432.9367; found, 432.9360; deviation: –1.5 ppm.

4-Iodoanisole (4)

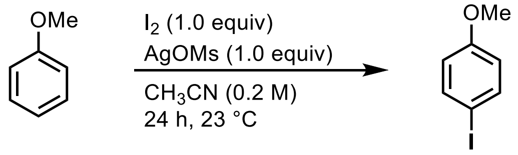

S4 **4,** 90% (*p*/*o*:45/1)

Under an ambient atmosphere, a 4 mL borosilicate vial equipped with magnetic stir bar was charged with molecular iodine (51.0 mg, 0.200 mmol, 1.00 equiv), AgOMs (40.6 mg, 0.200 mmol, 1.00 equiv), anisole (**S4**) (21.8 µL, 21.6 mg, 0.200 mmol, 1.00 equiv), and MeCN (1.0 mL, c = 0.20 M). The vial was capped, and the

reaction mixture was stirred at 23 °C for 24 h. The reaction mixture was diluted with EtOAc (5 mL), and the resulting mixture was filtered through a short pad of silica gel using EtOAc (5 mL) as eluent. The filtrate was collected and concentrated by rotary evaporation to afford 42 mg of **4** as a mixture of constitutional isomers a pale yellow oil (90% yield).The ratio of isomers was determined by integration of the ^{1}H-NMR in $CDCl_3$ of the methoxy groups.

R*f* (ethyl acetate:pentane, 05:95 (v:v)) = 0.63.

NMR Spectroscopy:

^{1}H NMR (500 MHz, $CDCl_3$, 25 ºC, δ): 7.27 (d, *J* = 9.0 Hz, 2H), 6.40 (d, *J* = 9.0 Hz, 2H), 3.49 (s, 3H).

^{13}C NMR (126 MHz, $CDCl_3$, 25 ºC, δ): 159.6, 138.3, 116.5, 82.8, 55.5.

HRMS-EI- Ion trap (m/z) calc'd for C_7H_7OI $[M]^{+}$, 233.9536; found, 233.9538; deviation: –0.8 ppm.

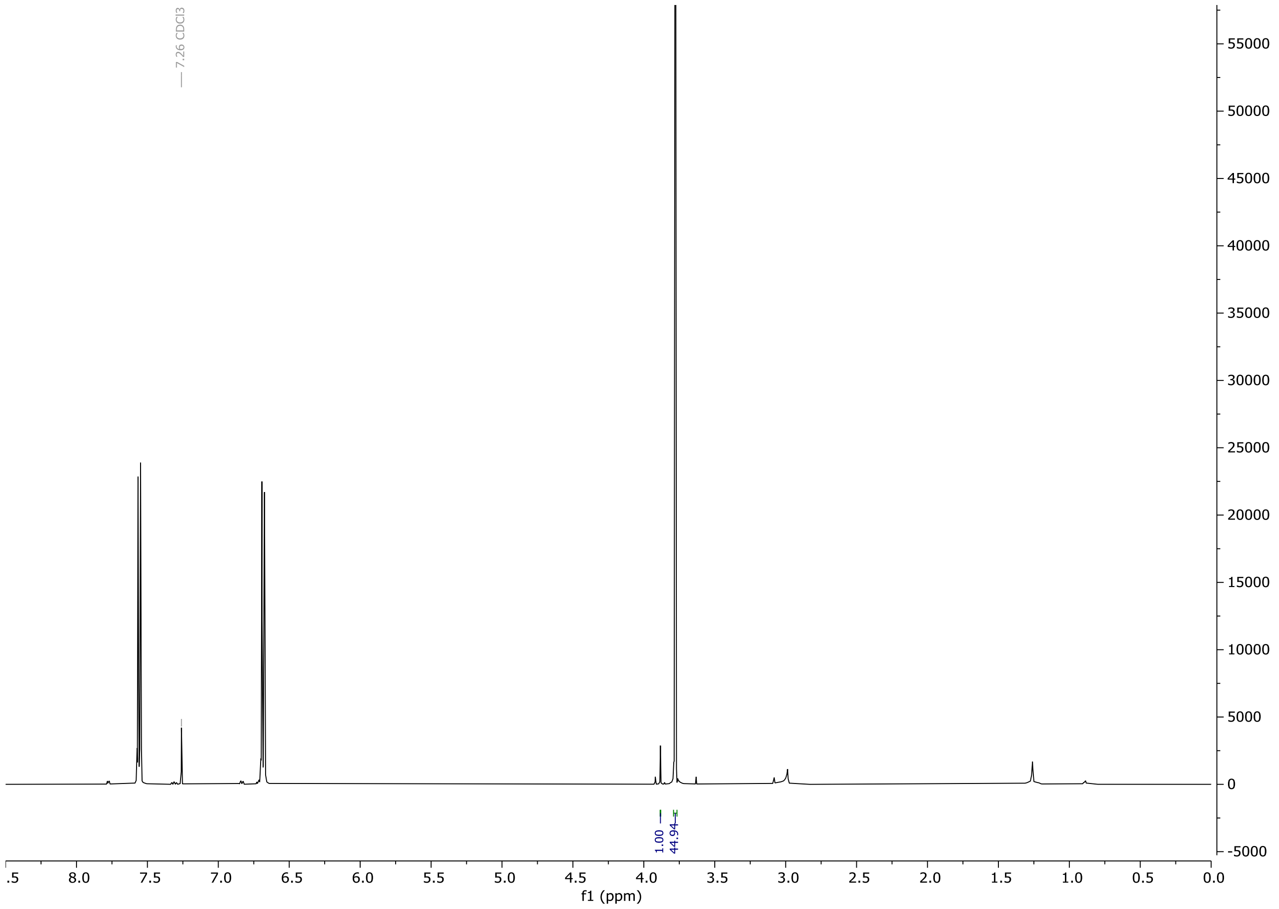

4-Iodophenol (5)

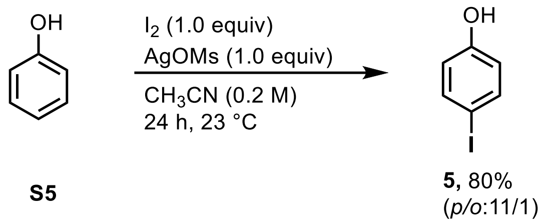

Under an ambient atmosphere, a 4 mL borosilicate vial equipped with magnetic stir bar was charged with molecular iodine (51.0 mg, 0.200 mmol, 1.00 equiv), AgOMs (40.6 mg, 0.200 mmol, 1.00 equiv), phenol (**S5**) (18.8 mg, 0.200 mmol, 1.00 equiv), and MeCN (1.0 mL, c = 0.20 M). The vial was capped, and the reaction mixture was stirred at 23 °C for 24 h. The reaction mixture was diluted with EtOAc (5 mL), and the resulting mixture was filtered through a short pad of silica gel using EtOAc (5 mL) as eluent. The filtrate was collected and concentrated by rotary evaporation to afford 35 mg of **5** as a mixture of constitutional isomers as pale yellow solid (80% yield).The ratio of isomers was determined by integration of the ^{1}H-NMR in $CDCl_3$ of the aromatic –CH protons.

R_f (ethyl acetate:pentane, 20:80 (v:v)) = 0.22.

NMR Spectroscopy:

^{1}H NMR (500 MHz, $CDCl_3$, 25 ºC, δ): 7.52 (d, J = 9.0 Hz, 2H), 6.63 (d, J = 9.0 Hz, 2H).

^{13}C NMR (126 MHz, $CDCl_3$, 25 ºC, δ): 155.4, 138.6, 117.9, 82.9.

HRMS-EI- Ion trap (m/z) calc'd for C_6H_5OI $[M]^+$, 219.9380; found, 219.9380; deviation: –0.5 ppm.

4-Iodoacetanilide (6)

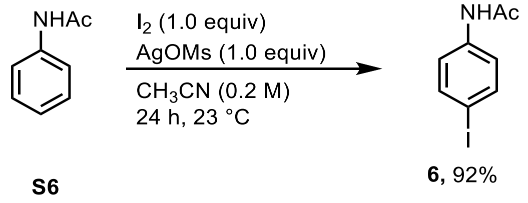

Under an ambient atmosphere, a 4 mL borosilicate vial equipped with magnetic stir bar was charged with molecular iodine (51.0 mg, 0.200 mmol, 1.00 equiv), AgOMs (40.6 mg, 0.200 mmol, 1.00 equiv), acetanilide (**S6**) (27.0 mg, 0.200 mmol, 1.00 equiv), and MeCN (1.0 mL, c = 0.20 M). The vial was capped, and the reaction mixture was stirred at 23 °C for 24 h. The reaction mixture was diluted with EtOAc (5 mL), and the resulting mixture was filtered through a short pad of silica gel using EtOAc (5 mL) as eluent. The filtrate was collected and concentrated by rotary evaporation. The residue was purified by column chromatography on silica gel eluting with a solvent mixture of ethyl acetate:pentane (30:70 (v:v)) to afford 48 mg of **6** as a colorless solid (92% yield).

R_f (ethyl acetate:pentane, 40:60 (v:v)) = 0.24.

NMR Spectroscopy:

[1]**H NMR** (300 MHz, CD_3CN, 25 ºC, δ): 8.36 (s, 1H), 7.62 (d, *J* = 8.8 Hz, 2H), 7.37 (d, *J* = 8.8 Hz, 2H), 2.04 (s, 3H).

[13]**C NMR** (75 MHz, CD_3CN, 25 ºC, δ): 169.7, 140.1, 138.6, 122.1, 86.6, 24.3.

HRMS-EI- Ion trap(m/z) calc'd for C_8H_8ONI $[M]^+$, 260.9645; found, 260.9648; deviation: –1.1 ppm.

4-Iodofluorobenzene (7)

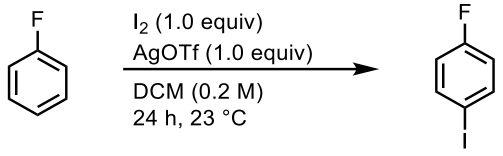

S7 **7,** 87% (*p*/*o*:12/1)

Under an ambient atmosphere, a 4 mL borosilicate vial equipped with magnetic stir bar was charged with molecular iodine (51.0 mg, 0.200 mmol, 1.00 equiv), AgOTf (51.0 mg, 0.200 mmol, 1.00 equiv), fluorobenzene (S**7**) (18.8 µL, 19.2 mg, 0.200 mmol, 1.00 equiv), and DCM (1.0 mL, c = 0.20 M). The vial was capped, and the reaction mixture was stirred at 23 °C for 24 h. The reaction mixture was diluted with EtOAc (5 mL), and the resulting mixture was filtered through a short pad of silica gel using EtOAc (5 mL) as eluent. The filtrate was collected and concentrated by rotary evaporation to afford 39 mg of **7** as a mixture of constitutional isomers as pale yellow liquid (87% yield).The ratio of isomers was determined by integration of the ^{1}H-NMR in CD_2Cl_2 of the aromatic –CH protons.

R*f* (ethyl acetate:pentane, 20:80 (v:v)) = 0.21.

NMR Spectroscopy:

[1]**H NMR** (500 MHz, CD_2Cl_2, 25 ºC, δ): 7.66 (dd, *J* = 8.9 Hz, 2H), 6.87 (m, *J* = 8.9 Hz, 2H).

[19]**F NMR** (471 MHz, CD_2Cl_2, 25° C): –77.64 (s, $^-$OTf), 115.1(m, *J* = 5.16, 1F).

[13]**C NMR** (126 MHz, CD_3CN, 25 ºC, δ): 163.7 (d, *J* = 245.1 Hz), 140.2 (d, *J* = 7.8 Hz), 118.7 (d, *J* = 22.2 Hz), 87.7 (d, *J* = 3.2 Hz).

HRMS-EI- Ion trap (m/z) calc'd for C_6H_4FI $[M]^+$, 221.9335; found, 221.9336; deviation: –0.1 ppm.

4-Iodonitrobenzene (8)

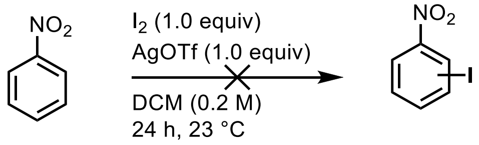

S8 **8,** 0%

Under an ambient atmosphere, a 4 mL borosilicate vial equipped with magnetic stir bar was charged with

molecular iodine (51.0 mg, 0.200 mmol, 1.00 equiv), AgOTf (51.0 mg, 0.200 mmol, 1.00 equiv), nitrobenzene (**S8**) (20.5 µL, 24.7 mg, 0.200 mmol, 1.00 equiv), and DCM (1.0 mL, c = 0.20 M). The vial was capped, and the reaction mixture was stirred at 23 °C for 24 h. The reaction mixture was diluted with EtOAc (5 mL), and the resulting mixture was filtered through a short pad of silica gel using EtOAc (5 mL) as eluent. The filtrate was collected and concentrated by rotary evaporation. No evidence of product was observed in ^{1}H NMR.

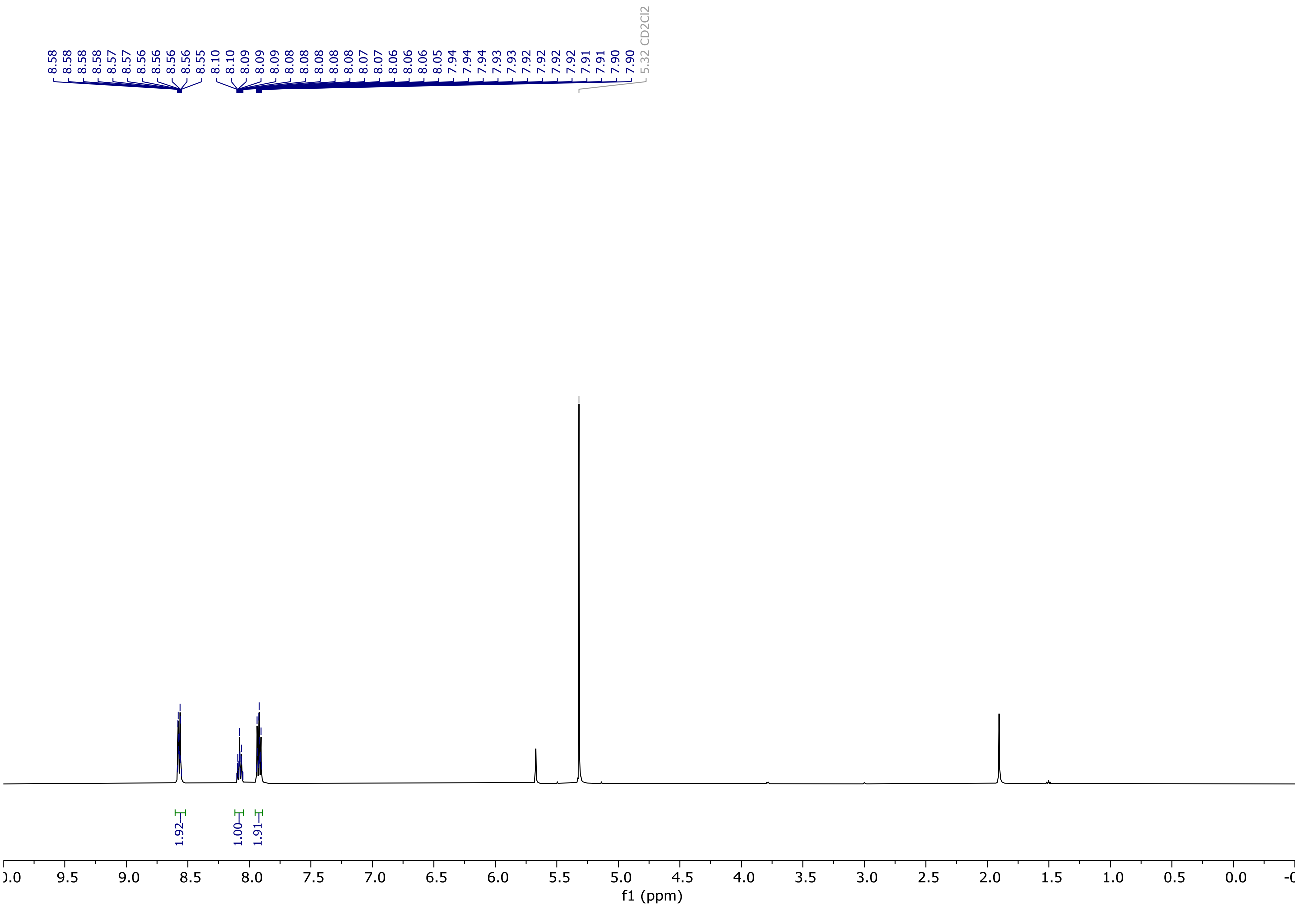

4-Iodoaniline (9)

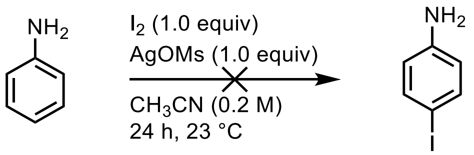

S9 **9,** 0%

Under an ambient atmosphere, a 4 mL borosilicate vial equipped with magnetic stir bar was charged with molecular iodine (51.0 mg, 0.200 mmol, 1.00 equiv), AgOMs (40.6 mg, 0.200 mmol, 1.00 equiv), aniline (**S9**) (18.3 µL, 18.6 mg, 0.200 mmol, 1.00 equiv), and MeCN (1.0 mL, c = 0.20 M). The vial was capped, and the reaction mixture was stirred at 23 °C for 24 h. The reaction mixture was diluted with EtOAc (5 mL), and the resulting mixture was filtered through a short pad of silica gel using EtOAc (5 mL) as eluent. The filtrate was collected and concentrated by rotary evaporation. No evidence of product was observed in ^{1}H NMR.

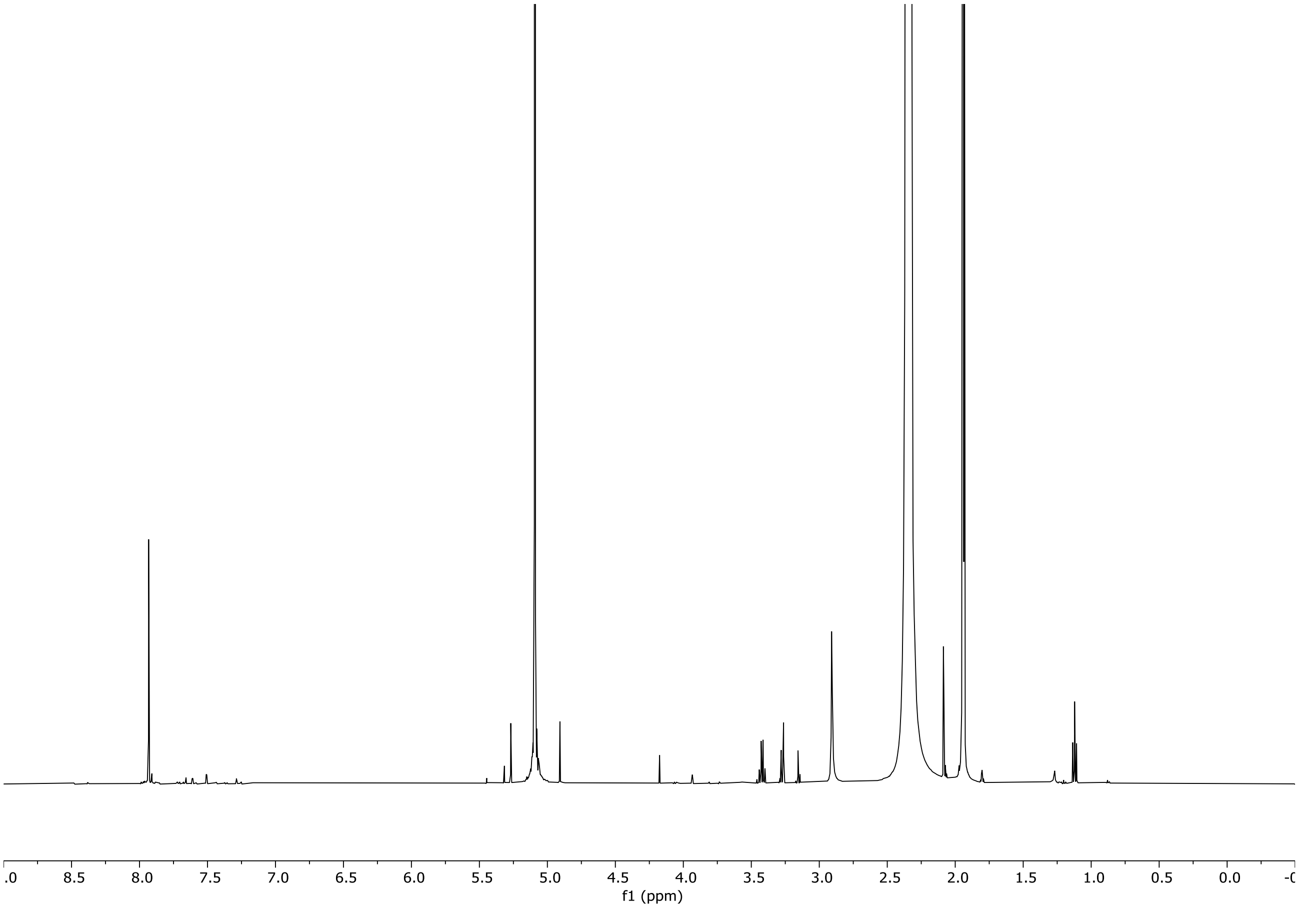

2-Acetyl-4-iodo-1-methylpyrrole (10)

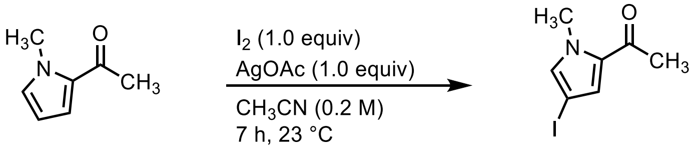

S10 **10,** 83%

Under an ambient atmosphere, a 4 mL borosilicate vial equipped with magnetic stir bar was charged with molecular iodine (50.8 mg, 0.200 mmol, 1.00 equiv), AgOAc (33.4 mg, 0.200 mmol, 1.00 equiv), 2-acetyl-1-methylpyrrole (**S10**) (23.7 µL, 24.6 mg, 0.200 mmol, 1.00 equiv), and MeCN (1.0 mL, c = 0.20 M). The vial was capped, and the reaction mixture was stirred at 23 °C for 7 h. The reaction mixture was diluted with EtOAc (5 mL), and the resulting mixture was filtered through a short pad of silica gel using EtOAc (5 mL) as eluent. The filtrate was collected and concentrated by rotary evaporation. The residue was purified by column chromatography on silica gel eluting with a solvent mixture of ethyl acetate:pentane (05:95 (v:v)) to afford 41.5 mg of **10** as a colorless solid (83% yield).

R_f (ethyl acetate:pentane, 10:90 (v:v)) = 0.29.

NMR Spectroscopy:

^{1}H NMR (500 MHz, $CDCl_3$, 25 ºC, δ): 7.00 (d, *J* = 1.8 Hz, 1H), 6.82 (d, *J* = 1.8 Hz, 1H), 3.91 (s, 3H), 2.39

(s, 3H).

^{13}C NMR (126 MHz, $CDCl_3$, 25 ºC, δ): 187.9, 135.1, 132.8, 126.1, 58.8, 37.8, 27.3.

HRMS-EI- Ion trap (m/z) calc'd for C_7H_8NOI $[M]^+$, 248.9647; found, 248.9645; deviation: –0.7 ppm.

3-Iodo-2,6-dimethoxypyridine (11)

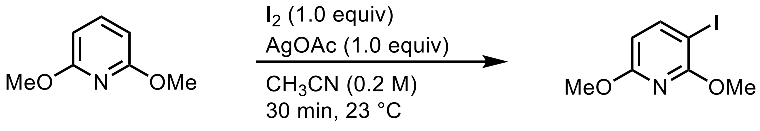

S11 **11,** 80%

Under an ambient atmosphere, a 4 mL borosilicate vial equipped with magnetic stir bar was charged with molecular iodine (50.8 mg, 0.200 mmol, 1.00 equiv), AgOAc (33.4 mg, 0.200 mmol, 1.00 equiv), 2,6-dimethoxypyridine (**S11**) (26.4 µL, 27.8 mg, 0.200 mmol, 1.00 equiv), and MeCN (1.0 mL, c = 0.20 M). The vial was capped, and the reaction mixture was stirred at 23 °C for 30 min. The reaction mixture was diluted with EtOAc (5 mL), and the resulting mixture was filtered through a short pad of silica gel using EtOAc (5 mL) as eluent. The filtrate was collected and concentrated by rotary evaporation. The residue was purified by column chromatography on silica gel eluting with a solvent mixture of ethyl acetate:pentane (01:99 (v:v)) to afford 43 mg of **11** as a colorless solid (80% yield).

R_f (pentane, 100 (v)) = 0.53.

NMR Spectroscopy:

^{1}H NMR (500 MHz, $CDCl_3$, 25 ºC, δ): 7.81 (d, J = 8.2 Hz, 1H), 6.16 (d, J = 8.2 Hz, 1H), 3.97 (s, 3H), 3.90 (s, 3H).

^{13}C NMR (126 MHz, $CDCl_3$, 25 ºC, δ): 144.0, 131.6, 127.8, 121.3, 73.9, 41.4, 19.0, 14.0.

HRMS-EI- Ion trap (m/z) calc'd for $C_7H_8NO_2I$ $[M]^+$, 264.9598; found, 264.9594; deviation: –1.5 ppm.

3-Iodoazaindazole (12)

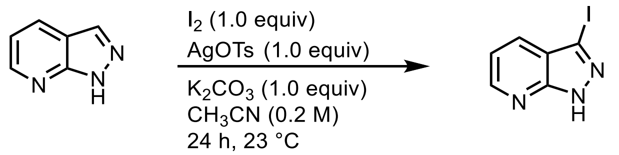

S12 **12,** 47%

Under an ambient atmosphere, a 4 mL borosilicate vial equipped with magnetic stir bar was charged with molecular iodine (50.80 mg, 0.200 mmol, 1.00 equiv), AgOTs (56.0 mg, 0.200 mmol, 1.00 equiv), azaindazole (**S12**) (23.90 mg, 0.200 mmol, 1.00 equiv), K_2CO_3 (27.60 mg, 0.200 mmol, 1.00 equiv), and MeCN (1.0 mL, c = 0.20 M). The vial was capped, and the reaction mixture was stirred at 23 °C for 24 h. The reaction mixture was diluted with EtOAc (5 mL), and the resulting mixture was filtered through a short pad of silica gel using EtOAc (5 mL) as eluent. The filtrate was transferred to a separatory funnel and 1N HCl (5 mL) was added. The

resulting mixture was extracted with EtOAc (3 × 15 mL). The combined organic layers were dried over $MgSO_4$, and concentrated under reduced pressure. The residue was purified by column chromatography on silica gel eluting with a solvent mixture of ethyl acetate:pentane (25:75 (v:v)) to afford 23 mg of **12** as a colorless solid (47% yield).

R_f (ethyl acetate:pentane, 30:70 (v:v)) = 0.33.

NMR Spectroscopy:

^{1}H NMR (600 MHz, DMSO, 25 ºC, δ): 14.08 (s, 1H), 8.57 (dd, *J* = 4.5, 1.5 Hz, 1H), 7.91 (ddd, *J* = 8.0, 1.6, 0.6 Hz, 1H), 7.25 (dd, *J* = 8.1, 4.5 Hz, 1H).

^{13}C NMR (151 MHz, DMSO, 25 ºC, δ): 151.7, 150.1, 129.9, 118.9, 117.7, 92.7.

HRMS-ESIpos- Ion trap (m/z) calc'd for $C_6H_5N_3I^+$ $[M+H]^+$, 245.9523; found, 245.9523; deviation: –0.2 ppm.

4-Iodopyrazole-3-carboxylate (13)

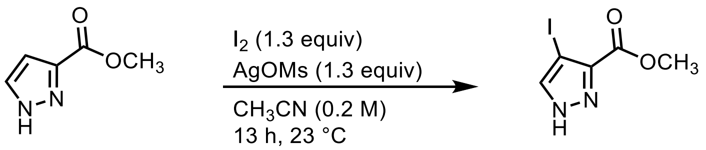

S13 **13,** 91%

Under an ambient atmosphere, a 4 mL borosilicate vial equipped with magnetic stir bar was charged with molecular iodine (66.0 mg, 0.260 mmol, 1.30 equiv), AgOMs (52.0 mg, 0.260 mmol, 1.30 equiv), 3-carboxylate pyrazole (**S13**) (25.20 mg, 0.200 mmol, 1.00 equiv), and MeCN (1.0 mL, c = 0.20 M). The vial was capped, and the reaction mixture was stirred at 23 °C for 13 h. The reaction mixture was diluted with EtOAc (5 mL), and the resulting mixture was filtered through a short pad of silica gel using EtOAc (5 mL) as eluent. The filtrate was collected and concentrated by rotary evaporation. The residue was purified by column chromatography on silica gel eluting with a solvent mixture of ethyl acetate:pentane (40:60 (v:v)) to afford 50.40 mg of **13** as a colorless solid (91% yield).

R_f (ethyl acetate:pentane, 40:60 (v:v)) = 0.33.

NMR Spectroscopy:

^{1}H NMR (500 MHz, CD_3CN, 25 ºC, δ): δ 7.76 (s, 1H), 3.86 (s, 3H).

^{13}C NMR (151 MHz, MeOD, 25 ºC, δ): 163.2, 144.0, 138.4, 60.8, 52.4.

HRMS-EI- Ion trap (m/z) calc'd for $C_5H_5N_2O_2I$ $[M]^+$ 251.9390; found, 251.9392; deviation: –0.8 ppm.

Note: Tautomeric forms of pyrazole were observed in the ^{13}C NMR spectrum.

2-Iodo-5-bromothiazole (14)

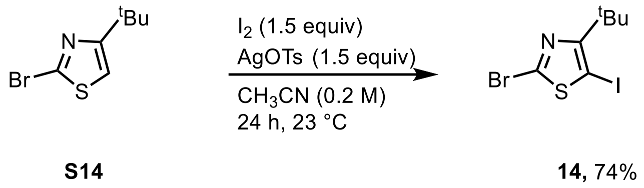

Under an ambient atmosphere, a 4 mL borosilicate vial equipped with magnetic stir bar was charged with molecular iodine (76.0 mg, 0.300 mmol, 1.50 equiv), AgOTs (61.0 mg, 0.300 mmol, 1.50 equiv), 5-bromothiazole (**S14**) (44.10 mg, 0.200 mmol, 1.00 equiv), and MeCN (1.0 mL, c = 0.20 M). The vial was capped, and the reaction mixture was stirred at 23 °C for 24 h. The reaction mixture was diluted with EtOAc (5 mL), and the resulting mixture was filtered through a short pad of silica gel using EtOAc (5 mL) as eluent. The filtrate was collected and concentrated under reduced pressure. The residue was purified by column chromatography on silica gel eluting with ethyl acetate:pentane (05:95 (v:v)) to afford 51 mg of **14** as a colorless liquid (74% yield).

R*f* (pentane) = 0.29.

NMR Spectroscopy:

^{1}H NMR (300 MHz, DMSO, 25 ºC, δ): 1.43 (s, 9H).

^{13}C NMR (75 MHz, DMSO, 25 ºC, δ): 164.5, 137.3, 68.9, 36.2, 30.0.

HRMS-EI- Ion trap (m/z) calc'd for C_7H_9NISBr $[M]^+$, 344.8682; found, 344.8678; deviation: –1.2 ppm.

6-Chloro-7-iododezapurine (15)

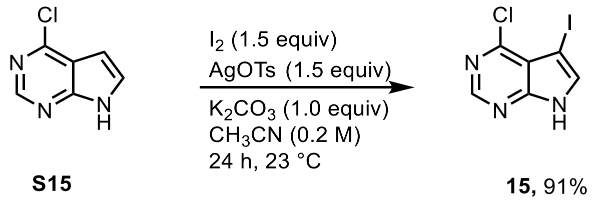

Under an ambient atmosphere, a 4 mL borosilicate vial equipped with magnetic stir bar was charged with molecular iodine (76.0 mg, 0.300 mmol, 1.50 equiv), AgOTs (61.0 mg, 0.300 mmol, 1.50 equiv), 6-chloro-dezapurine (**S15**) (44.0 mg, 0.200 mmol, 1.00 equiv), K_2CO_3 (27.60 mg, 0.200 mmol, 1.00 equiv), and MeCN (1.0 mL, c = 0.20 M). The vial was capped, and the reaction mixture was stirred at 23 °C for 24 h. The reaction mixture was diluted with EtOAc (5 mL), and the resulting mixture was filtered through a short pad of silica gel using EtOAc (5 mL) as eluent. The filtrate was transferred to a separatory funnel and 1N HCl (5 mL) was added. The resulting mixture was extracted with EtOAc (3 × 15 mL). The combined organic layers were dried over $MgSO_4$, and concentrated under reduced pressure. The filtrate was collected and concentrated under reduced pressure. The residue was purified by column chromatography on silica gel eluting with ethyl acetate:pentane (30:70 (v:v)) to afford 55.90 mg of **15** as a colorless solid (91% yield).

R$_f$ (ethyl acetate:pentane, 40:60 (v:v)) = 0.35.

NMR Spectroscopy:

^{1}H NMR (500 MHz, DMSO, 25 ºC, δ): 12.94 (s, 1H), 8.58 (s, 1H), 7.93 (s, 1H).

^{13}C NMR (126 MHz, DMSO, 25 ºC, δ): 151.5, 150.7, 150.5, 133.9, 115.8, 51.6.

HRMS-EI- Ion trap (m/z) calc'd for $C_6H_3N_3ClI$ [M]$^+$, 278.9058; found, 278.9055; deviation: –1.2 ppm.

2-(5-Iodothiophene)-benzothiazole (16)

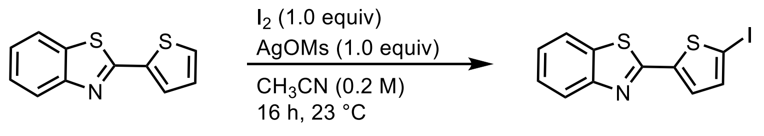

S16 **16,** 95%

Under an ambient atmosphere, a 4 mL borosilicate vial equipped with magnetic stir bar was charged with molecular iodine (50.8 mg, 0.200 mmol, 1.00 equiv), AgOMs (40.6 mg, 0.200 mmol, 1.00 equiv), 2-(2-thienyl)-benzothiazol (**S16**) (43.50 mg, 0.200 mmol, 1.00 equiv), and MeCN (1.0 mL, c = 0.20 M). The vial was capped, and the reaction mixture was stirred at 23° C for 16 h. The reaction mixture was diluted with EtOAc (5 mL), and the resulting mixture was filtered through a short pad of silica gel using EtOAc (5 mL) as eluent. The filtrate was collected and concentrated by rotary evaporation. The residue was purified by column chromatography on silica gel eluting with a solvent mixture of ethyl acetate:pentane (02:98 (v:v)) to afford 65 mg of **16** as a colorless solid (95% yield).

R$_f$ (ethyl acetate:pentane, 10:90 (v:v)) = 0.65.

NMR Spectroscopy:

^{1}H NMR (500 MHz, $CDCl_3$, 25 ºC, δ): 8.01 (dt, J = 8.2, 0.9 Hz, 1H), 7.84 (dt, J = 8.1, 1.0 Hz, 1H), 7.48 (ddd, J = 8.3, 7.2, 1.3 Hz, 1H), 7.37 (ddd, J = 8.2, 7.2, 1.2 Hz, 1H), 7.30 – 7.27 (m, 2H).

^{13}C NMR (126 MHz, $CDCl_3$, 25 ºC, δ): 160.1, 153.7, 143.3, 138.0, 134.7, 129.6, 126.7, 125.6, 123.2, 121.6, 79.0.

HRMS-EI- Ion trap (m/z) calc'd for $C_{11}H_6NS_2I$ [M]$^+$, 342.8987; found, 342.8981; deviation: –1.8 ppm.

4-Iodopyrazole-1-benzoic acid (17)

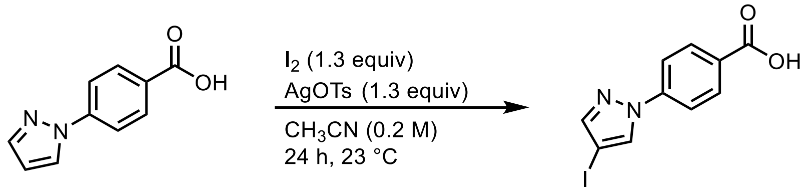

S17 **17,** 81%

Under an ambient atmosphere, a 4 mL borosilicate vial equipped with magnetic stir bar was charged with

molecular iodine (66.0 mg, 0.260 mmol, 1.30 equiv), AgOTs (72.6 mg, 0.260 mmol, 1.30 equiv), 4-(1H-Pyrazol-1-yl)-benzoic acid (**S17**) (37.70 mg, 0.200 mmol, 1.00 equiv), and MeCN (1.0 mL, c = 0.20 M). The vial was capped, and the reaction mixture was stirred at 23 °C for 24 h. The reaction mixture was diluted with EtOAc (5 mL), and the resulting mixture was filtered through a short pad of silica gel using EtOAc (5 mL) as eluent. The filtrate was collected and concentrated by rotary evaporation. The residue was purified by column chromatography on silica gel eluting with a solvent mixture of ethyl acetate:pentane (10:90 (v:v)) to afford 51 mg of **17** as a colorless solid (81% yield).

R_f (ethyl acetate:pentane, 40:60 (v:v)) = 0.13.

NMR Spectroscopy:

^{1}H NMR (300 MHz, DMSO, 25 ºC, δ): 13.07 (s, 1H), 8.83 (s, 1H), 8.10 – 8.01 (m, 2H), 7.99 – 7.89 (m, 3H).

^{13}C NMR (75 MHz, DMSO, 25 ºC, δ): 166.6, 146.3, 141.9, 132.5, 130.9, 128.9, 117.9, 61.7.

HRMS-EI- Ion trap (m/z) calc'd for $C_{10}H_6N_2O_2I$ $[M]^+$, 312.9483; found, 312.9480; deviation: –1.1 ppm.

Iodinated-ipriflavone (18)

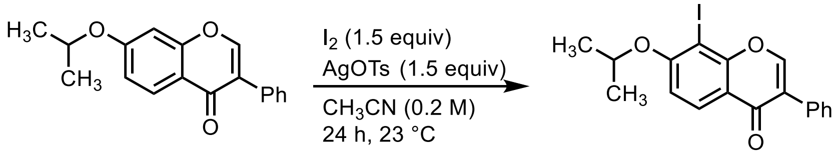

S18 **18,** 89%

Under an ambient atmosphere, a 4 mL borosilicate vial equipped with magnetic stir bar was charged with molecular iodine (76.0 mg, 0.300 mmol, 1.50 equiv), AgOTs (61.0 mg, 0.300 mmol, 1.50 equiv), ipriflavone (**S18**) (56.10 mg, 0.200 mmol, 1.00 equiv), and MeCN (1.0 mL, c = 0.20 M). The vial was capped, and the reaction mixture was stirred at 23 °C for 24 h. The reaction mixture was diluted with EtOAc (5 mL), and the resulting mixture was filtered through a short pad of silica gel using EtOAc (5 mL) as eluent. The filtrate was collected and concentrated by rotary evaporation. The residue was purified by column chromatography on silica gel eluting with a solvent mixture of ethyl acetate:pentane (40:60 (v:v)) to afford 72 mg of **18** as a colorless solid (89% yield).

R_f (ethyl acetate:pentane, 30:70 (v:v)) = 0.60.

NMR Spectroscopy:

^{1}H NMR (500 MHz, $CDCl_3$, 25 ºC, δ): 8.26 (d, *J* = 8.9 Hz, 1H), 8.06 (s, 1H), 7.63 – 7.52 (m, 2H), 7.47 – 7.42 (m, 2H), 7.41 – 7.37 (m, 1H), 6.95 (d, *J* = 9.0 Hz, 1H), 4.79 (hept, *J* = 6.1 Hz, 1H), 1.47 (d, *J* = 6.1 Hz, 6H).

^{13}C NMR (126 MHz, $CDCl_3$, 25 ºC, δ): 175.7, 162.0, 157.0, 153.2, 131.8, 129.1, 128.5, 128.4, 128.2, 125.1, 119.38, 73.1, 22.2.

HRMS-ESIpos- Ion trap (m/z) calc'd for $C_{18}H_{16}O_3INa^+$ $[M+Na]^+$, 407.0138; found, 407.0139; deviation: 0.2

ppm.

Iodinated-coumarin1 (19)

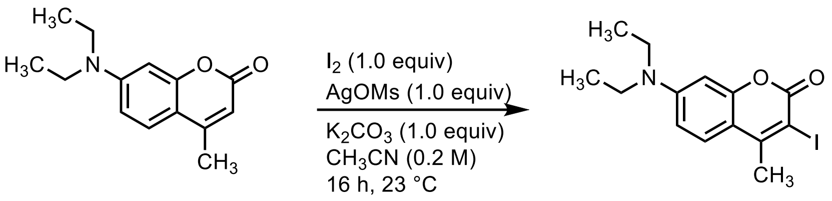

S19 **19,** 95%

Under an ambient atmosphere, a 4 mL borosilicate vial equipped with magnetic stir bar was charged with molecular iodine (51.0 mg, 0.200 mmol, 1.00 equiv), AgOMs (40.60 mg, 0.200 mmol, 1.00 equiv), coumarin1 (**S19**) (46.30 mg, 0.200 mmol, 1.00 equiv), K_2CO_3 (27.60 mg, 0.200 mmol, 1.00 equiv), and MeCN (1.0 mL, c = 0.20 M). The vial was capped, and the reaction mixture was stirred at 23 °C for 16 h. The reaction mixture was diluted with EtOAc (5 mL), and the resulting mixture was filtered through a short pad of silica gel using EtOAc (5 mL) as eluent. The filtrate was transferred to a separatory funnel and 1N HCl (5 mL) was added. The resulting mixture was extracted with EtOAc (3 × 15 mL). The combined organic layers were dried over $MgSO_4$, and concentrated under reduced pressure. The residue was purified by column chromatography on silica gel eluting with a solvent mixture of ethyl acetate:pentane (10:90 (v:v)) to afford 68 mg of **19** as a pale yellow solid (95% yield).

R_f (ethyl acetate:pentane, 20:80 (v:v)) = 0.43.

NMR Spectroscopy:

^{1}H NMR (500 MHz, $CDCl_3$, 25 ºC, δ): 7.44 (d, *J* = 9.1 Hz, 1H), 6.56 (dd, *J* = 9.1, 2.6 Hz, 1H), 6.48 (d, *J* = 2.7 Hz, 1H), 3.41 (q, *J* = 7.1 Hz, 4H), 2.58 (s, 3H), 1.20 (t, *J* = 7.1 Hz, 6H).

^{13}C NMR (126 MHz, $CDCl_3$, 25 ºC, δ): 158.9, 156.8, 155.2, 150.9, 126.4, 108.9, 97.2, 83.8, 44.9, 25.0, 12.6.

HRMS-ESIpos- Ion trap (m/z) calc'd for $C_{14}H_{16}NO_2INa^+$ $[M+Na]^+$, 380.0118; found, 380.0115; deviation: 0.7 ppm.

Gram-scale synthesis: Iodinated-coumarin1 (19)

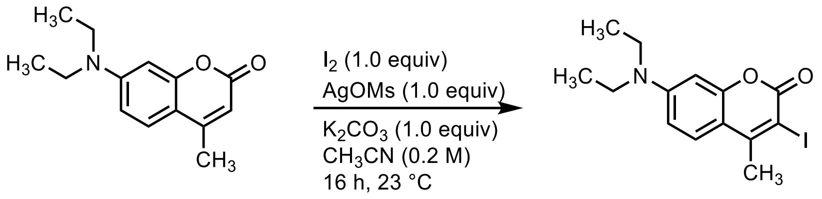

S19 **19,** 90%

Under an ambient atmosphere, a 50 mL round-bottom flask with a magnetic stir bar was charged with molecular iodine (1.09 g, 4.32 mmol, 1.00 equiv), AgOMs (0.880 g, 4.32 mmol, 1.00 equiv), coumarin1 (**S19**) (1.00 g, 4.32

mmol, 1.00 equiv), and K_2CO_3 (0.600 g, 4.32 mmol, 1.00 equiv), and MeCN (21.6 mL, c = 0.200 M). The round bottom flask was capped with a rubber septum, and the reaction mixture was stirred at 23° C for 24 h. The reaction mixture was diluted with EtOAc (20 mL), and the resulting mixture was filtered through a short pad of silica gel using EtOAc (20 mL) as eluent. The filtrate was transferred to a separatory funnel and 1N HCl (20 mL) was added. The layers were separated and the aqueous layer was extracted with EtOAc (3 × 30 mL). The combined organic layers were washed with brine (15 mL), dried over $MgSO_4$, and concentrated under reduced pressure to afford 1.4 g of **19** as a pale brown solid (90% yield).

R_f (ethyl acetate: pentane, 20:80 (v:v)) = 0.43.

NMR Spectroscopy:

^{1}H NMR (500 MHz, $CDCl_3$, 25 ºC, δ): 7.44 (d, *J* = 9.1 Hz, 1H), 6.56 (dd, *J* = 9.1, 2.6 Hz, 1H), 6.48 (d, *J* = 2.7 Hz, 1H), 3.41 (q, *J* = 7.1 Hz, 4H), 2.58 (s, 3H), 1.20 (t, *J* = 7.1 Hz, 6H).

^{13}C NMR (126 MHz, $CDCl_3$, 25 ºC, δ): 158.9, 156.8, 155.2, 150.9, 126.4, 108.9, 97.2, 83.8, 44.9, 25.0, 12.6.

HRMS-EI- Ion trap (m/z) calc'd for $C_{14}H_{16}NIO_2^+$ [M]$^+$, 357.0220; found, 357.0229; deviation: –2.5 ppm.

Iodinated-procymidone (20)

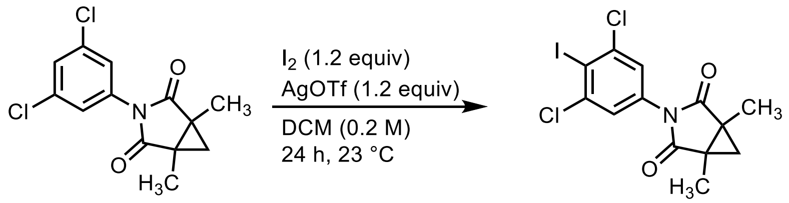

S20 **20,** 82%

Under an ambient atmosphere, a 4 mL borosilicate vial equipped with magnetic stir bar was charged with molecular iodine (61.0 mg, 0.240 mmol, 1.20 equiv), AgOTf (62.0 mg, 0.240 mmol, 1.20 equiv), procymidone (**S20**) (57.80 mg, 0.200 mmol, 1.00 equiv), and DCM (1.0 mL, c = 0.20 M). The vial was capped, and the reaction mixture was stirred at 23 °C for 24 h. The reaction mixture was diluted with EtOAc (5 mL), and the resulting mixture was filtered through a short pad of silica gel using EtOAc (5 mL) as eluent. H_2O (10 mL) was added and the resulting mixture was transferred to a separation funnel. The layers were separated and the aqueous layer was extracted with EtOAc (3 × 15 mL). The combined organic layers were washed with brine (10 mL), dried over Na_2SO_4, and concentrated under reduced pressure. The residue was purified by column chromatography on silica gel eluting with a solvent mixture of ethyl acetate:pentane (10:90 (v:v)) to afford 51 mg of **20** as a colorless liquid (82% yield).

R_f (ethyl acetate:pentane, 10:90 (v:v)) = 0.41.

NMR Spectroscopy:

^{1}H NMR (500 MHz, $CDCl_3$, 25 ºC, δ): 7.35 (s, 2H), 1.73 (d, *J* = 4.7 Hz, 1H), 1.49 (s, 6H), 1.20 (d, *J* = 4.7

Hz, 1H).

^{13}C NMR (126 MHz, $CDCl_3$, 25 ºC, δ): 175.7, 140.9, 133.3, 124.7, 103.1, 32.9, 30.3, 10.1.

HRMS-EI- Ion trap (m/z) calc'd for $C_{13}H_0NCl_2ONaI$ $[M+Na]^+$, 431.9057; found, 431.9025; deviation: –0.3 ppm.

Iodinated-boscalid triflate salt (21)

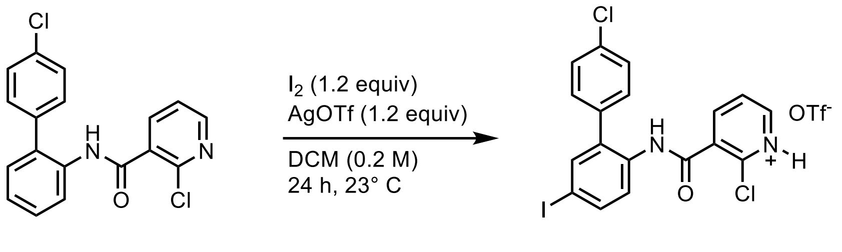

S21 **21,** 96%

Under an ambient atmosphere, a 4 mL borosilicate vial equipped with magnetic stir bar was charged with molecular iodine (61.0 mg, 0.240 mmol, 1.00 equiv), AgOTf (62.0 mg, 0.240 mmol, 1.20 equiv), boscalid (**S21**) (68.70 mg, 0.200 mmol, 1.00 equiv), and DCM (1.0 mL, c = 0.20 M). The vial was capped, and the reaction mixture was stirred at 23 °C for 24 h. The reaction mixture was diluted with EtOAc (5 mL), and the resulting mixture was filtered through a short pad of silica gel using EtOAc (5 mL) as eluent. The residue was purified by column chromatography on silica gel eluting with a solvent mixture of ethyl acetate:pentane (20:80 (v:v)) to afford 119 mg of **21** as a colorless triflate salt (96% yield).

R_f (ethyl acetate:pentane, 30:70 (v:v)) = 0.20.

NMR Spectroscopy:

^{1}H NMR (500 MHz, DMSO, 25 ºC, δ): 10.17 (s, 1H), 8.47 (dd, J = 4.8, 1.9 Hz, 1H), 7.88 (dd, J = 7.6, 2.0 Hz, 1H), 7.81 (dd, J = 8.4, 2.1 Hz, 1H), 7.72 (d, J = 2.1 Hz, 1H), 7.53 – 7.42 (m, 6H).

^{19}F NMR (471 MHz, DMSO, 25° C): –77.73.

^{13}C NMR (151 MHz, DMSO, 25 ºC, δ): 164.1, 150.4, 146.4, 138.4, 138.3, 137.9, 137.0, 136.2, 133.9, 132.8, 132.6, 130.7, 129.2, 128.4, 123.0, 120.7 (q, J = 322.3 Hz), 91.9.

HRMS-ESIpos- Ion trap (m/z) calc'd for $C_{18}H_{12}N_2IO_1Cl_2Na^+$ $[M+Na]^+$, 468.9366; found, 468.9367; deviation: –0.2 ppm.

Iodinated-diclofenac (22)

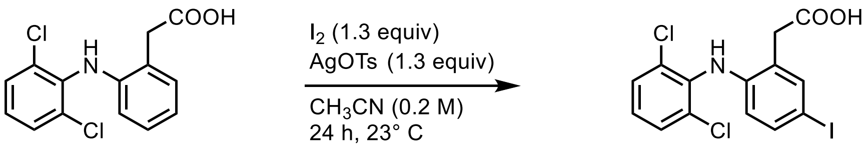

S22 **22,** 96%

Under an ambient atmosphere, a 4 mL borosilicate vial equipped with magnetic stir bar was charged with molecular iodine (66.0 mg, 0.260 mmol, 1.00 equiv), AgOTs (67.0 mg, 0.260 mmol, 1.00 equiv), diclofenac (**S22**) (59.0 mg, 0.200 mmol, 1.00 equiv), and CH_3CN (1.0 mL, c = 0.20 M). The vial was capped, and the reaction mixture was stirred at 23 °C for 24 h. The reaction mixture was diluted with EtOAc (10 mL), and the resulting mixture was filtered through a short pad of silica gel using EtOAc (10 mL) as eluent. The residue was purified by column chromatography on silica gel eluting with a solvent mixture of ethyl acetate:pentane (30:70 (v:v)) to afford 101 mg of **22** as a pale yellow solid (96% yield).

R_f (ethyl acetate:pentane, 40:60 (v:v)) = 0.56.

NMR Spectroscopy:

^{1}H NMR (300 MHz, DMSO, 25 ºC, δ): 12.67 (s, 1H), 7.59 – 7.47 (m, 3H), 7.35 (dd, *J* = 8.5, 2.2 Hz, 1H), 7.29 (s, 1H), 7.22 (dd, *J* = 8.5, 7.7 Hz, 1H), 6.04 (d, *J* = 8.5 Hz, 1H), 3.69 (s, 2H).

^{13}C NMR (75 MHz, DMSO, 25 ºC, δ): 172.7, 142.9, 138.9, 136.4, 135.9, 131.0, 129.2, 126.4, 126.0, 117.5, 82.5, 36.8.

HRMS-ESIneg- Ion trap (m/z) calc'd for $C_{14}H_9O_2Cl_2NI$ $[M–H]^-$, 419.9065; found, 419.9060; deviation: –1.0 ppm.

Iodinated-napropamid (23)

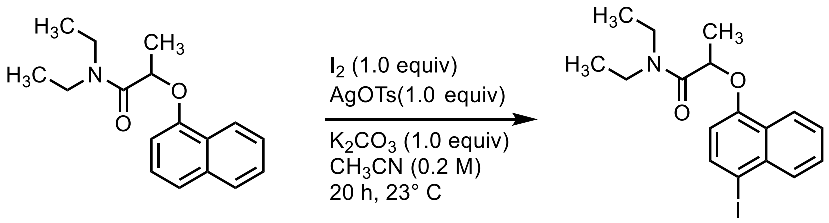

S23 (±) **23 (±),** 99%

Under an ambient atmosphere, a 4 mL borosilicate vial equipped with magnetic stir bar was charged with molecular iodine (51.0 mg, 0.200 mmol, 1.00 equiv), AgOTs (56.0 mg, 0.200 mmol, 1.00 equiv), napropamid (**S23**) (54.30 mg, 0.200 mmol, 1.00 equiv), K_2CO_3 (27.70 mg, 0.200 mmol, 1.00 equiv), and CH_3CN (1.0 mL, c = 0.20 M). The vial was capped, and the reaction mixture was stirred at 23 °C for 20 h. The reaction mixture was diluted with EtOAc (5 mL), and the resulting mixture was filtered through a short pad of silica gel using EtOAc (5 mL) as eluent. The filtrate was transferred to a separatory funnel and 1N HCl (5 mL) was added. The layers were separated and the aqueous layer was extracted with EtOAc (3 × 15 mL). The combined organic layers were washed with brine (10 mL), dried over $MgSO_4$, and concentrated under reduced pressure to afford 79 mg of **23** as a colorless solid (99% yield).

R_f (ethyl acetate:pentane, 40:60 (v:v)) = 0.35.

NMR Spectroscopy:

^{1}H NMR (500 MHz, CD_3CN, 25 ºC, δ): 8.30 – 8.27 (m, 1H), 7.98 (dt, *J* = 8.6, 0.9 Hz, 1H), 7.94 (d, *J* = 8.2

Hz, 1H), 7.63 (ddd, J = 8.4, 6.8, 1.4 Hz, 1H), 7.56 (ddd, J = 8.2, 6.9, 1.2 Hz, 1H), 6.55 (d, J = 8.2 Hz, 1H), 5.19 (q, J = 6.5 Hz, 1H), 3.43 (qd, J = 7.2, 2.5 Hz, 2H), 3.32 (ddt, J = 16.8, 13.6, 6.7 Hz, 2H), 1.61 (d, J = 6.5 Hz, 3H), 1.11 (t, J = 7.1 Hz, 3H), 1.04 (t, J = 7.1 Hz, 3H).

^{13}C NMR (126 MHz, CD_3CN, 25 ºC, δ): 170.2, 155.3, 138.1, 135.6, 132.4, 129.4, 127.6, 127.3, 123.6, 108.7, 88.6, 73.2, 42.1, 40.9, 18.5, 14.6, 13.0.

HRMS-ESIpos- Ion trap (m/z) calc'd for $C_{17}H_{20}NIO_2Na^+$ $[M+Na]^+$, 420.0429; found, 420.0430; deviation: 0.4 ppm.

Iodinated-fenobirinacid (24)

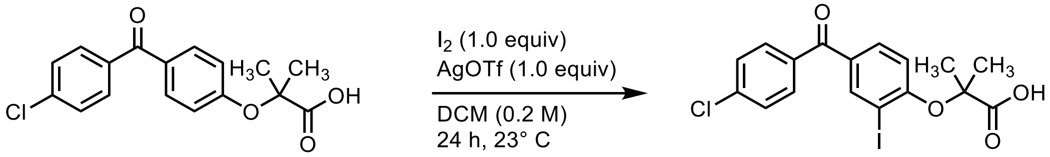

S24 **24,** 98%

Under an ambient atmosphere, a 4 mL borosilicate vial equipped with magnetic stir bar was charged with molecular iodine (51.0 mg, 0.200 mmol, 1.00 equiv), AgOTf (51.0 mg, 0.200 mmol, 1.00 equiv), fenobirinacid (**24**) (63.7 mg, 0.200 mmol, 1.00 equiv), and DCM (1.0 mL, c = 0.20 M). The vial was capped, and the reaction mixture was stirred at 23 °C for 24 h. The reaction mixture was diluted with EtOAc (5 mL), and the resulting mixture was filtered through a short pad of silica gel using EtOAc (5 mL) as eluent. H_2O (10 mL) was added and the resulting mixture was transferred to a separation funnel. The layers were separated and the aqueous layer was extracted with EtOAc (3 × 15 mL). The combined organic layers were washed with brine (10 mL), dried over Na_2SO_4, and concentrated under reduced pressure. The residue was purified by column chromatography on silica gel eluting with a solvent mixture of ethyl acetate:pentane (30:70 (v:v)) to afford 61 mg of **24** as a colorless solid (98% yield).

R_f (ethyl acetate:pentane, 40:60 (v:v)) = 0.54.

NMR Spectroscopy:

^{1}H NMR (500 MHz, CD_2Cl_2, 25 ºC, δ): 8.25 (d, J = 2.1 Hz, 1H), 7.70 (d, J = 8.5 Hz, 3H), 7.49 (d, J = 8.5 Hz, 2H), 6.89 (d, J = 8.6 Hz, 1H), 1.76 (s, 6H).

^{13}C NMR (126 MHz, CD_2Cl_2, 25 ºC, δ): 193.1, 176.5, 158.4, 142.1, 139.14, 136.1, 133.1, 131.9, 131.6, 129.1, 116.5, 90.1, 81.7, 25.5.

HRMS-ESIpos- Ion trap (m/z) calc'd for $C_{17}H_{15}O_4ClI^+$ $[M+H]^+$, 444.9698; found, 444.9698; deviation: –0.1 ppm.

Iodinated-strychnine triflate salt (25)

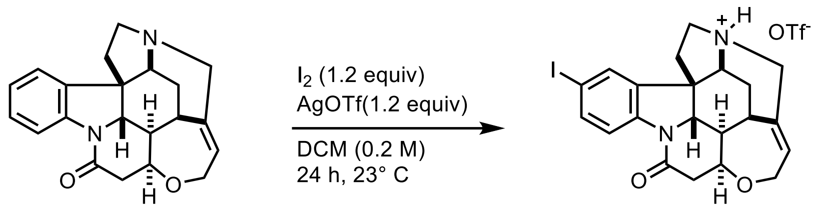

S25 **25,** 96%

Under an ambient atmosphere, a 4 mL borosilicate vial equipped with magnetic stir bar was charged with molecular iodine (61.0 mg, 0.240 mmol, 1.20 equiv), AgOTf (62.0 mg, 0.240 mmol, 1.20 equiv), strychnine (**S25**) (67.0 mg, 0.200 mmol, 1.00 equiv, and DCM (1.0 mL, c = 0.20 M). The vial was capped, and the reaction mixture was stirred at 23 °C for 24 h. The reaction mixture was diluted with EtOAc (5 mL), and the resulting mixture was filtered through a short pad of silica gel using EtOAc (5 mL) as eluent. The filtrate was collected and concentrated under reduced pressure. The product was dissolved in 5 mL DCM and precipitated with 10 mL Et_2O. The suspension was decanted, and the solid was dried in vacuo to afford 117 mg of **25** as an off-white solid (96% yield).

NMR Spectroscopy:

^{1}H NMR (600 MHz, DMSO, 25 ºC, δ): 10.32 (t, *J* = 6.3 Hz, 1H), 7.85 (d, *J* = 1.8 Hz, 1H), 7.65 (dd, *J* = 8.4, 1.8 Hz, 1H), 6.33 (td, *J* = 6.6, 5.8, 3.3 Hz, 1H), 4.35 (dt, *J* = 8.3, 3.5 Hz, 1H), 4.08 (d, *J* = 10.9 Hz, 1H), 3.77 – 3.69 (m, 1H), 3.52 (d, *J* = 13.7 Hz, 1H), 3.32 (s, 1H), 3.14 (tt, *J* = 12.8, 6.3 Hz, 1H), 2.96 (dd, *J* = 17.1, 8.3 Hz, 1H), 2.65 – 2.59 (m, 1H), 2.48 (t, *J* = 4.2 Hz, 1H), 2.18 (dd, *J* = 13.1, 6.0 Hz, 1H), 1.95 (td, *J* = 13.2, 7.8 Hz, 1H), 1.63 (dt, *J* = 15.4, 2.2 Hz, 1H), 1.47 (dt, *J* = 10.9, 3.2 Hz, 1H).

^{19}F NMR (471 MHz, DMSO, 25° C): –77.73.

^{13}C NMR (151 MHz, DMSO, 25 ºC, δ): 169.0, 141.7, 137.9, 134.9, 132.7, 132.5, 131.7, 120.7 (q, *J* = 322.3 Hz), 117.4, 87.3, 75.8, 63.1, 61.3, 58.8, 51.7, 51.2, 50.9, 45.9, 41.1, 40.2, 29.5, 24.3.

HRMS-ESIpos- Ion trap (m/z) calc'd for $C_{21}H_{22}N_2IO_2^+$ $[M+H]^+$, 461.0720; found, 461.0718; deviation: 0.5 ppm.

Formation of acetyl hypoiodite

AgOAc + I_2 → IOAc + AgI (23 °C, CD_3CN)

1 equiv. 1 equiv.

Under an ambient atmosphere, a 4 mL borosilicate vial equipped with magnetic stir bar was charged with molecular iodine (25.0 mg, 0.100 mmol, 1.00 equiv), AgOAc (16.7 mg, 0.100 mmol, 1.00 equiv), and CD_3CN (0.5 mL, c = 0.20 M). The vial was capped, and the reaction mixture was stirred at 23 °C for 10 minutes. The reaction mixture was then filtered through a short pad of silica gel using CD_3CN (0.5 mL) as eluent. The filtrate was collected directly in the NMR tube. A ^{1}H-NMR spectrum of the crude reaction mixture shows signal of IOAc, matches with the reported literature.[2]

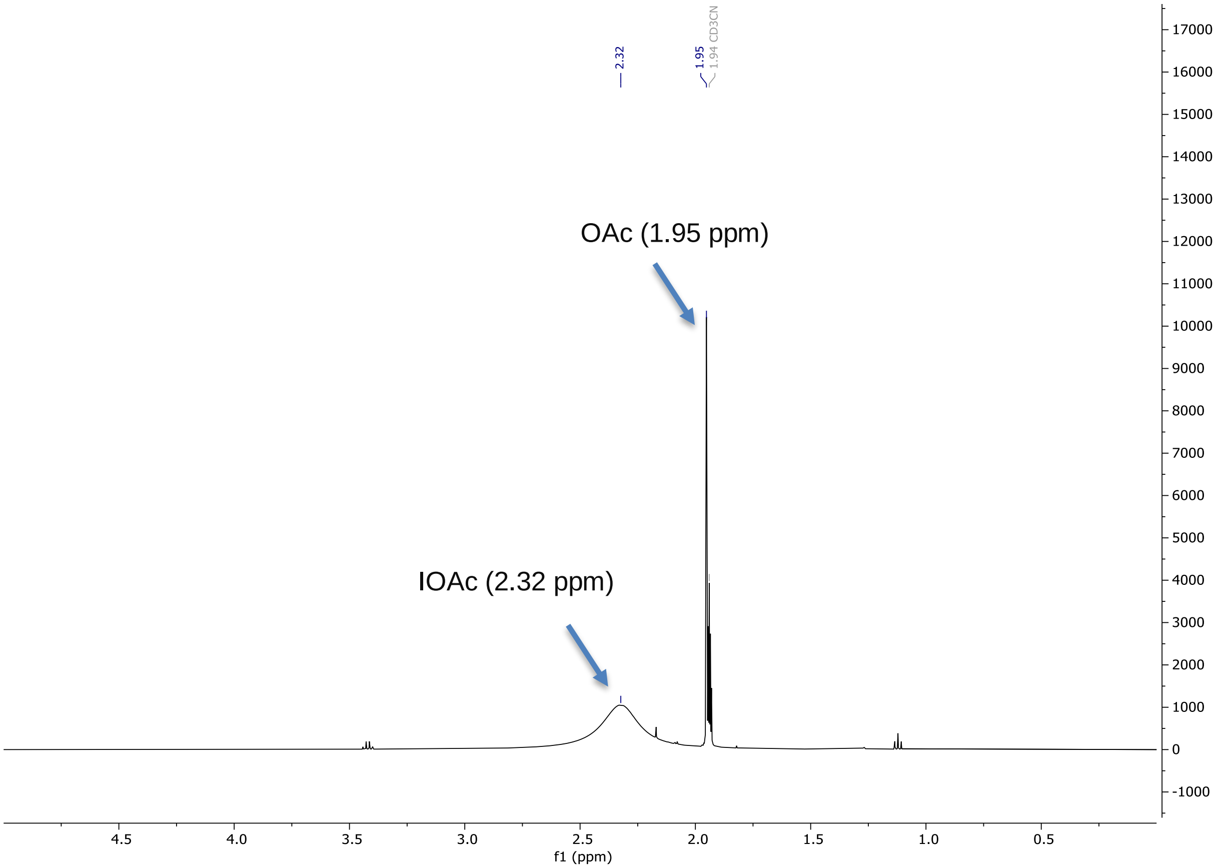

Figure S1. ^{1}H NMR spectra of iodine in presence of AgOAc in CD_3CN.

Comparison to NIS method

In comparison to modern C–H iodination methods, that use NIS as an iodinating reagent; our method is applicable to a broader electronic scope of aromatic (hetero) arenes and avoids double iodination (Table S1).

Table S1. Comparison of hypoiodites method with NIS.

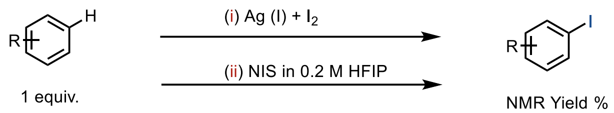

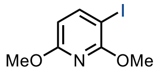	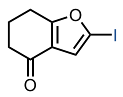	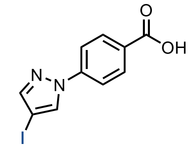	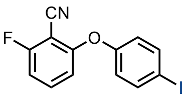
89%[i]<br>78% & 20% DI[ii]	80%[i]<br>54%[ii]	81%[i]<br>traces[ii]	92%[i]<br>0%[ii]
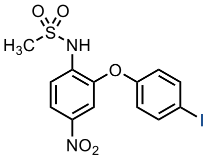<br>from *nimesulide*	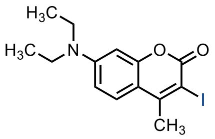<br>from *coumarin 1*	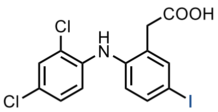<br>from *diclofenac*	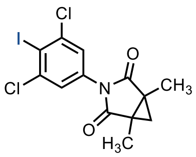<br>from *procymidone*
92%[i]<br>0%[ii]	98%[i]<br>58%[ii]	99%[i]<br>0%[ii]	82%[i]<br>0%[ii]
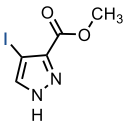	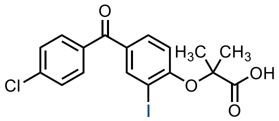<br>from *fenobirinacid*	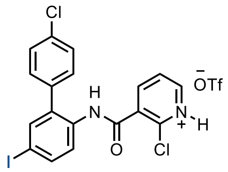<br>from *boscalid*	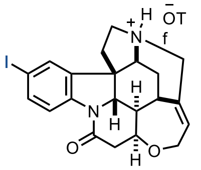<br>from *strychnine*
91%[i]<br>66%[ii]	98%[i]<br>traces[ii]	96%[i]<br>0%[ii]	98%[i]<br>0%[ii]

Electrochemical data

Cyclic Voltammograms

Cyclic voltammograms were recorded using an Autolab PGSTAT204 potentiostat and a Pt working electrode, a Ag/AgCl reference electrode and a Pt sheet auxiliary electrode. The voltammograms were recorded at a Ag/AgCl reference electrode and a Pt sheet auxiliary electrode. The voltammograms were recorded at room temperature in 0.1 M tetrabutylammonium hexafluorophosphate in MeCN (3 mL) containing Ag(**I**) salts and molecular iodine (I_2). Ag**I** was filtered off before measuring the redox potentional of the corresponding hypoiodites. The scan rate was 100 mV s–1.

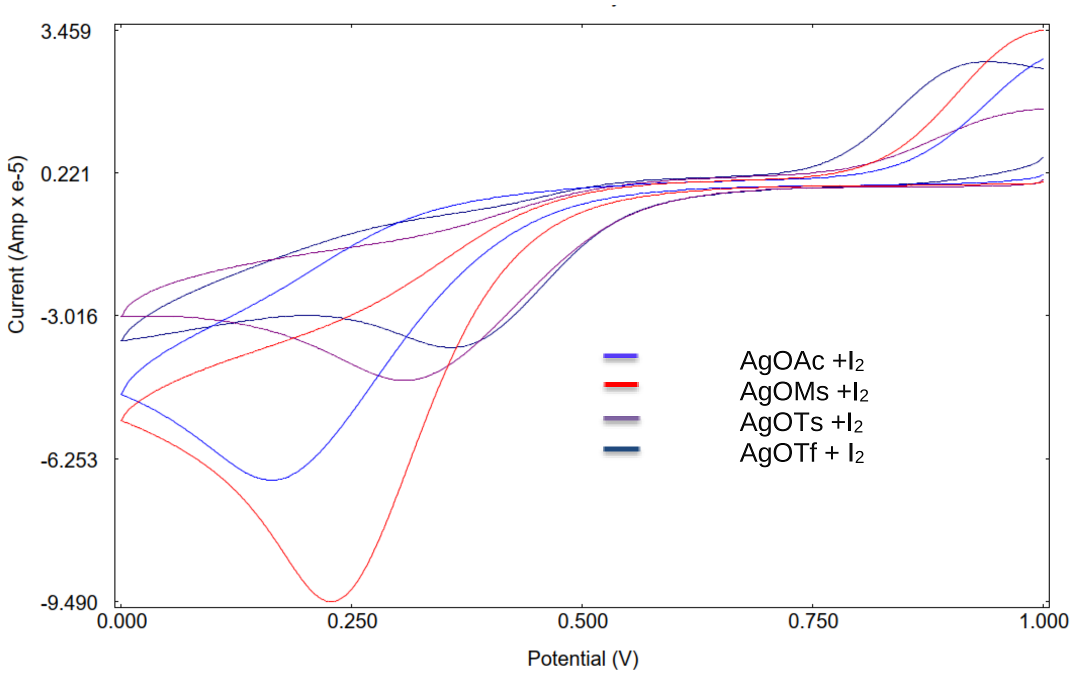

Figure S2. Cyclic voltammetry of hypoiodites in MeCN.

Note: CV of AgOTf and I_2 was measured in DCM.

Table S2. Value of E^{red} vs Ag/AgCl in MeCN.

Entry	Hypoiodites	E^{red} (V vs Ag/AgCl)
1	AgOAc + I_2	0.16
2	AgOMs + I_2	0.23
3	AgOTs + I_2	0.31
4	AgOTf + I_2	0.36

Table S3. Modulation of sulfonyl based hypoiodites.

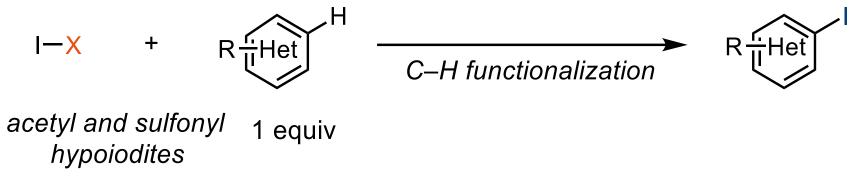

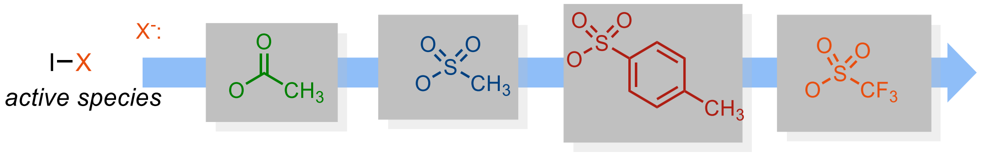

pKa–HX	5	–1	–3	–14
Leaving group	AcO^-	MsO^-	TsO^-	TfO^-
E^+ (V vs Ag/AgCl)	0.17	0.23	0.31	0.36

SPECTROSCOPIC DATA

^{1}H NMR of 2-Iodo-tetrahydrobenzofuranone (2)

$CDCl_3$, 500 MHz, 25 ºC

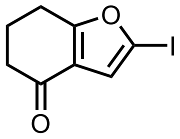

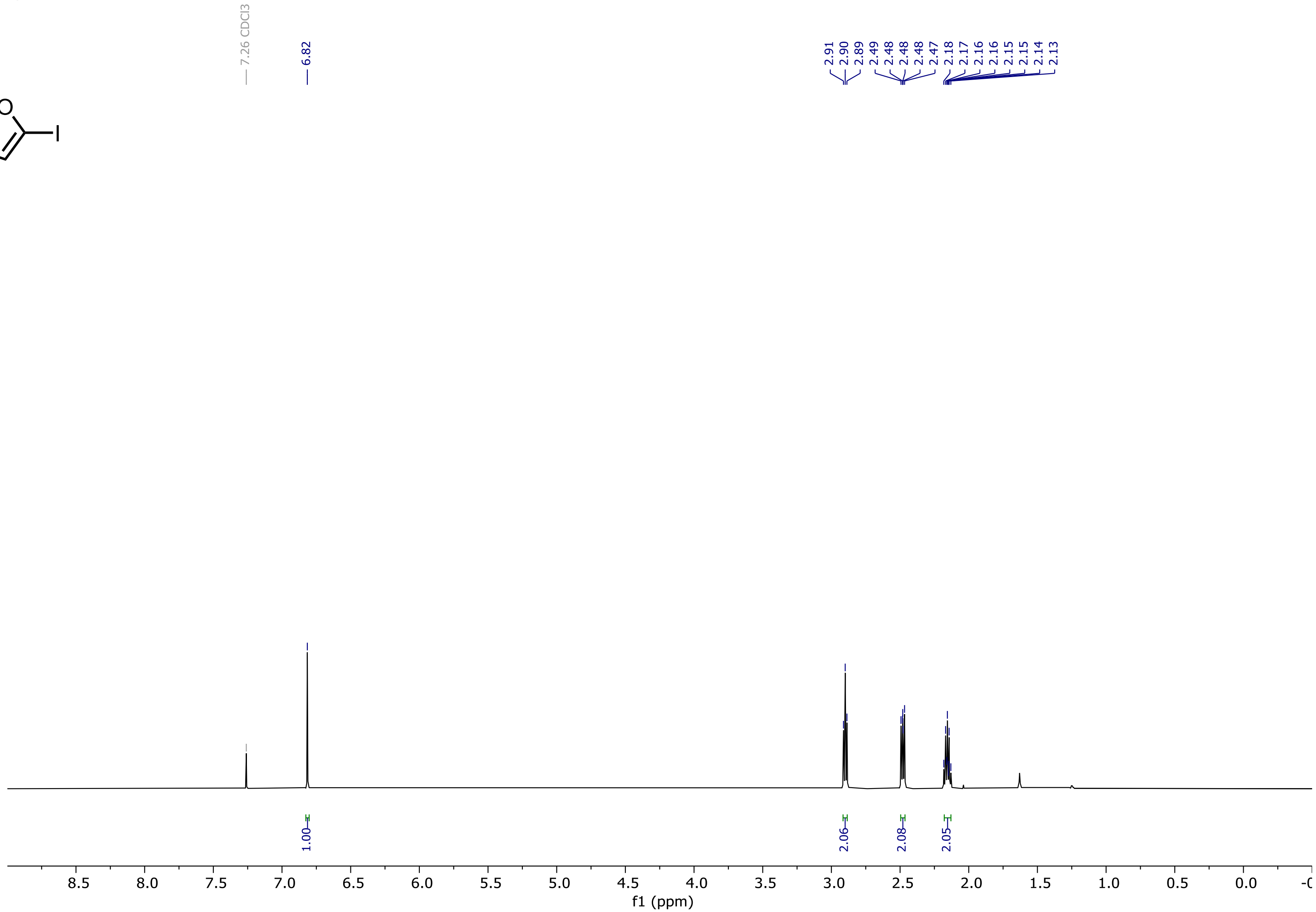

^{13}C NMR of 2-Iodo-tetrahydrobenzofuranone (2)

$CDCl_3$, 126 MHz, 23 ºC

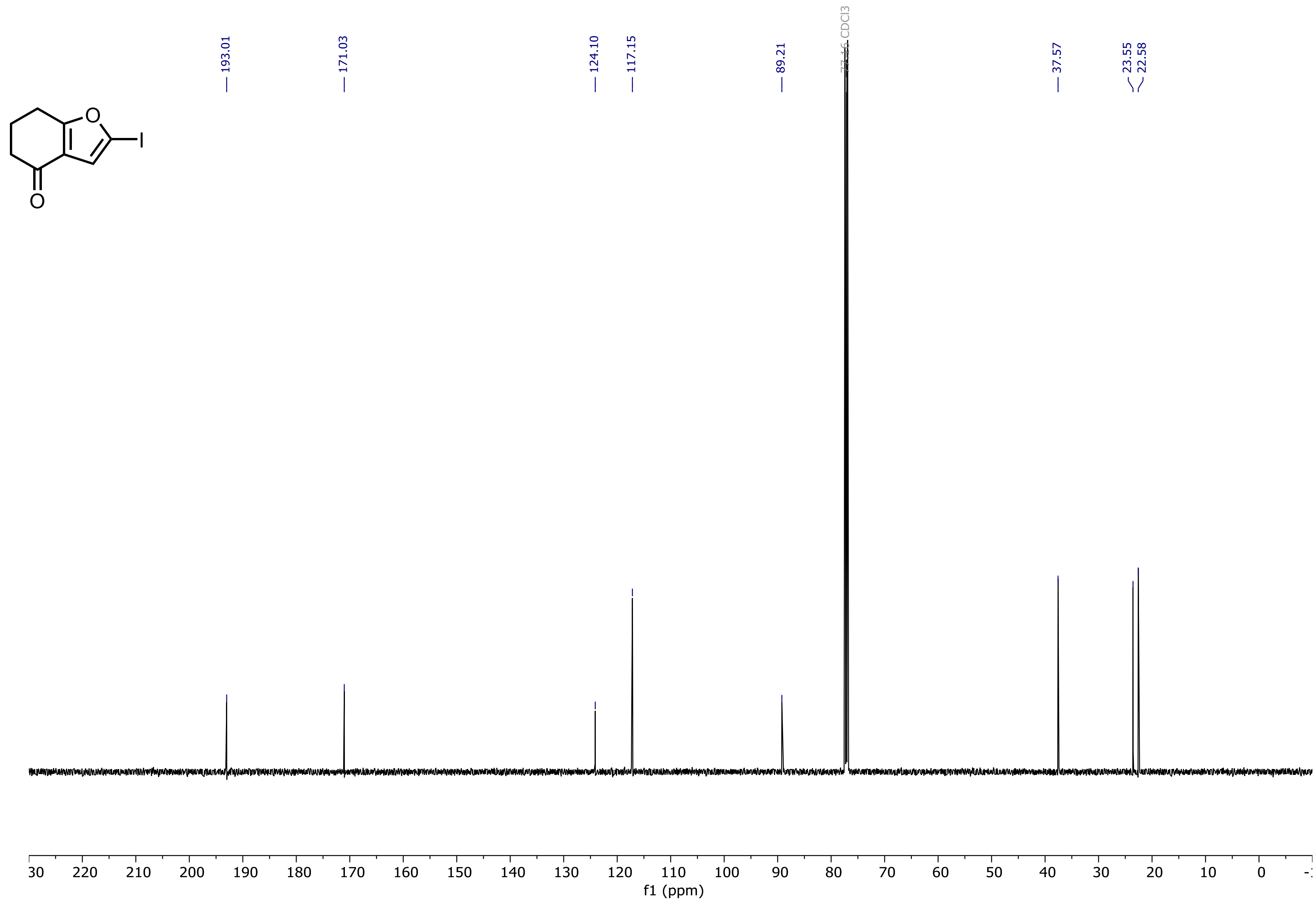

^{1}H NMR of Iodinated-nimesulide (3)

$(CD_3)_2SO$, 500 MHz, 25 ºC

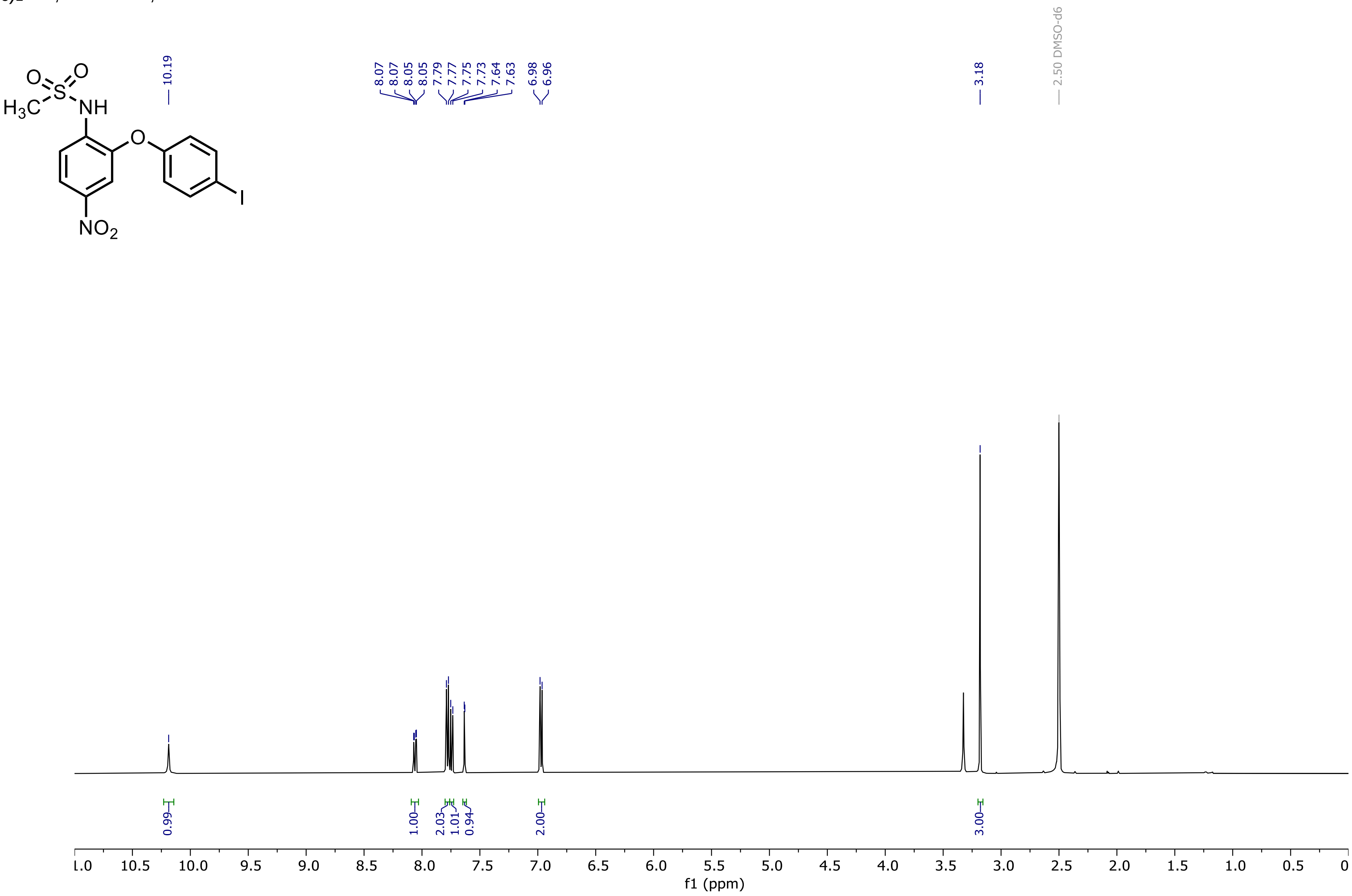

^{13}C NMR of Iodinated-nimesulide (3)

$(CD_3)_2SO$, 126 MHz, 25 ºC

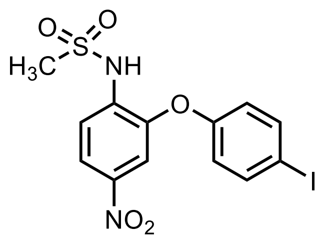

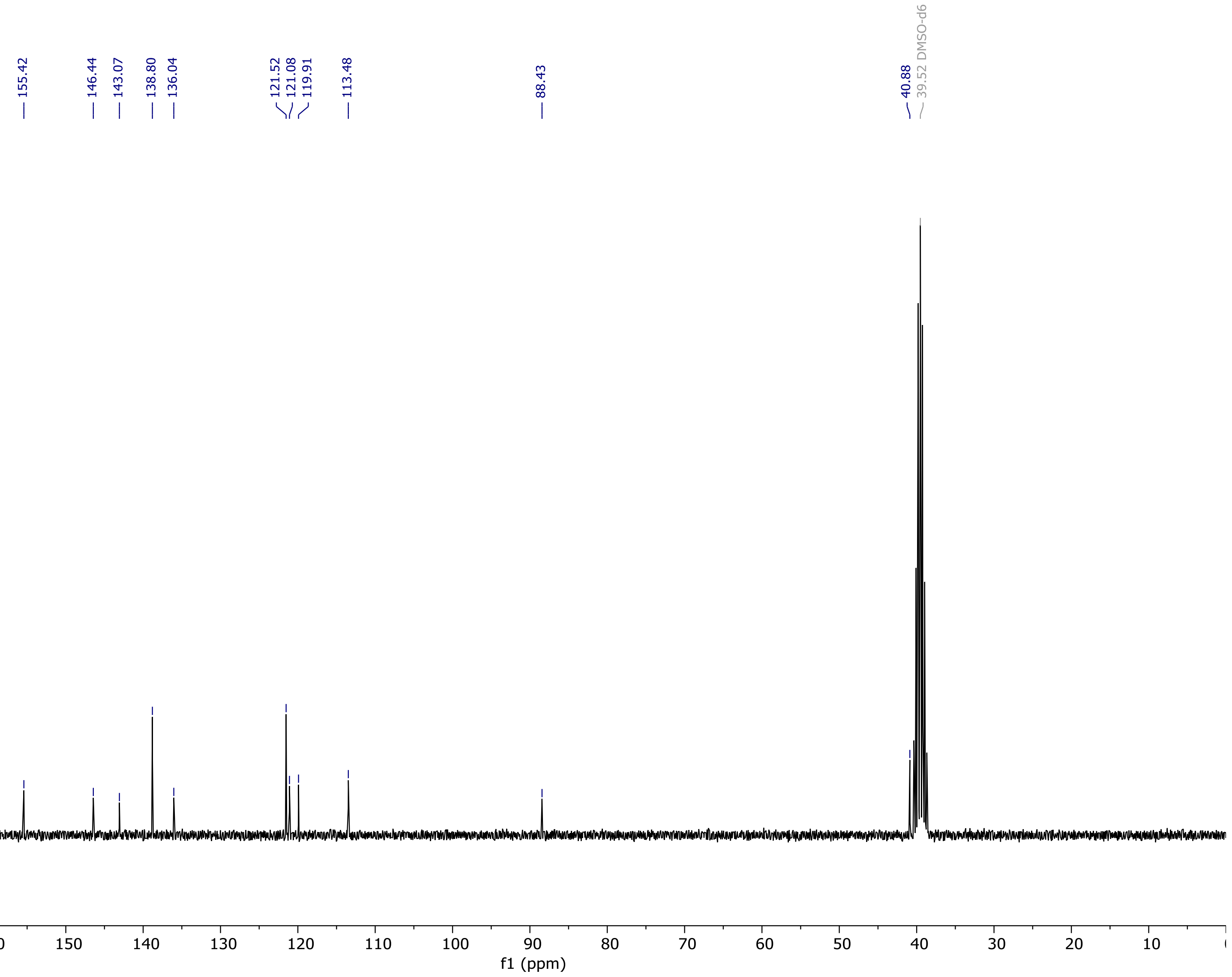

^{1}H NMR of 4-Iodoanisole (4)

$CDCl_3$, 500 MHz, 25 ºC

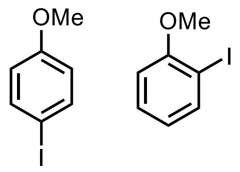

45:1

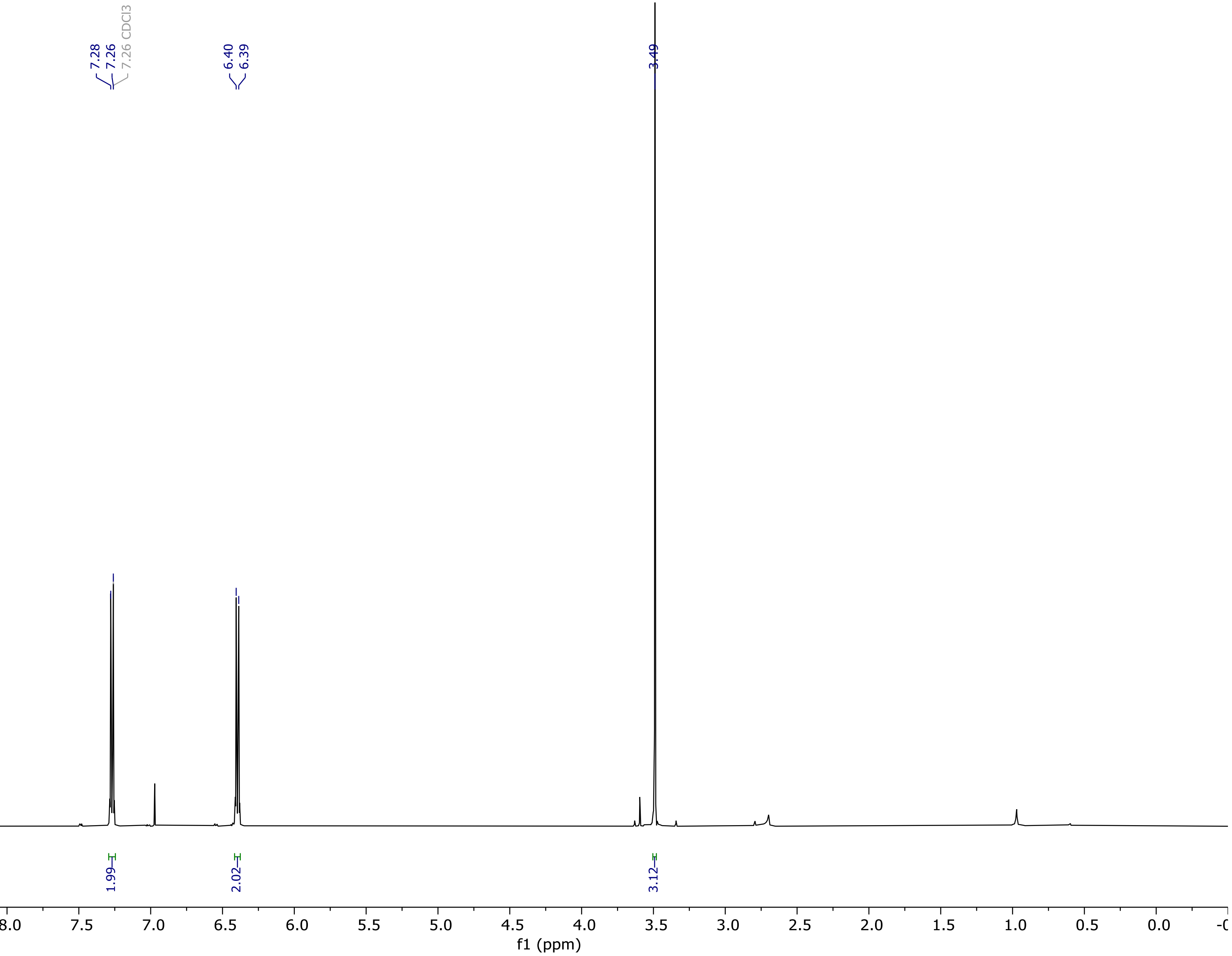

^{13}C NMR of 4-Iodoanisole (4)

$CDCl_3$, 126 MHz, 25 ºC

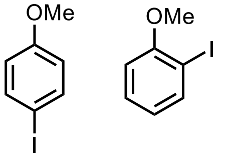

45:1

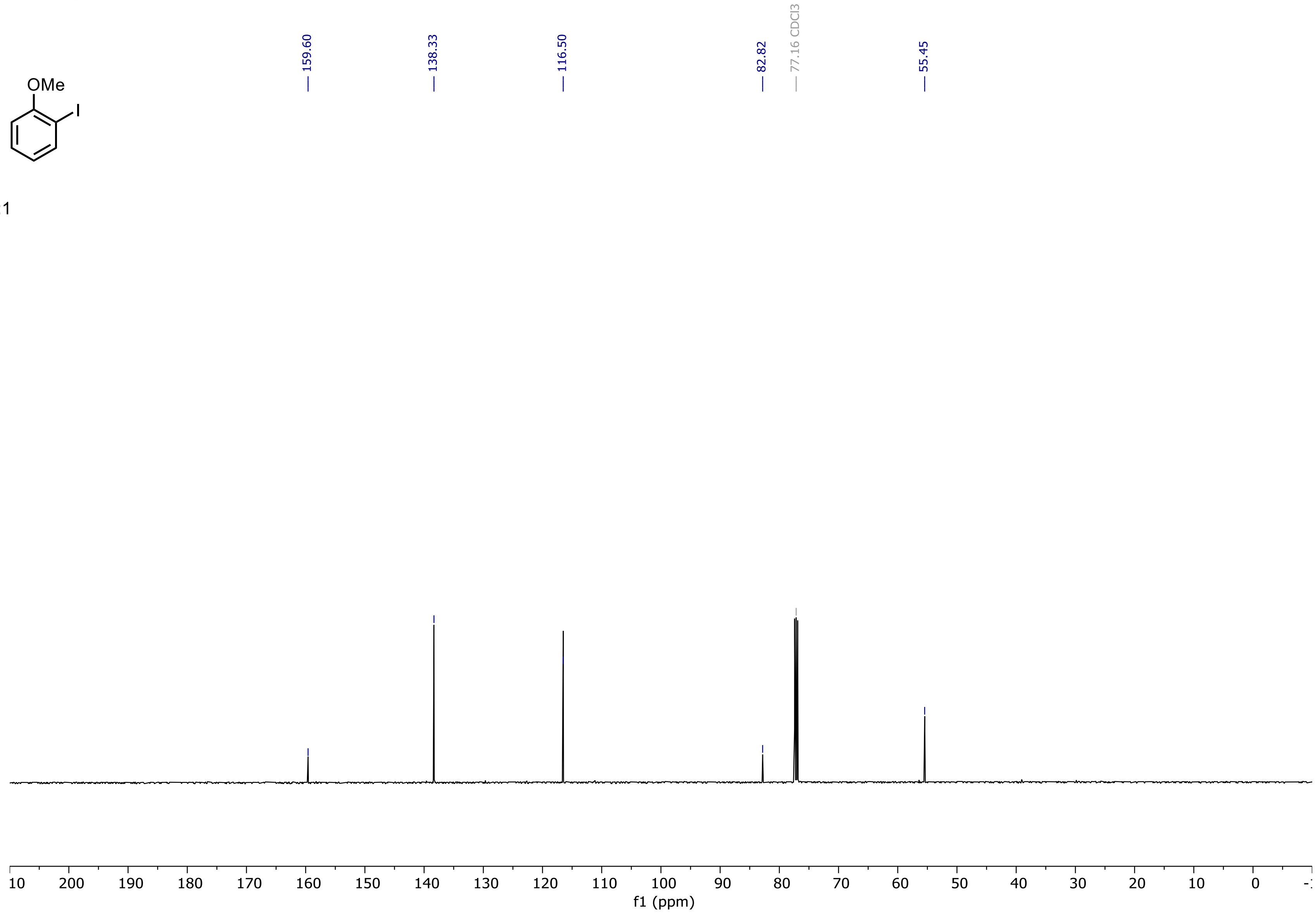

^{1}H NMR of 4-Iodophenol (5)

$CDCl_3$, 500 MHz, 25 ºC

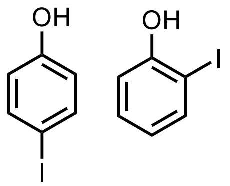

11:1

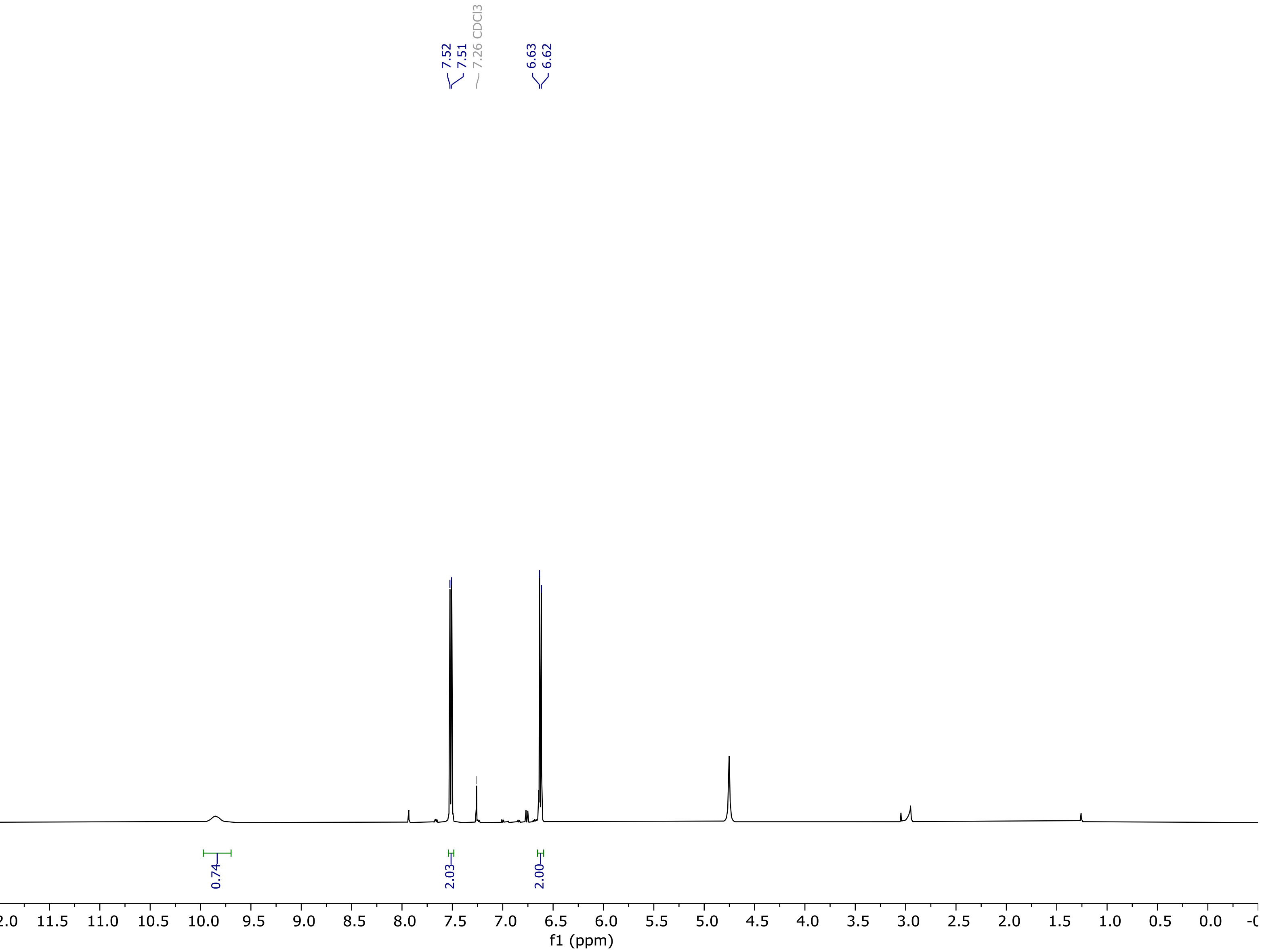

^{13}C NMR of 4-Iodophenol (5)

$CDCl_3$, 126 MHz, 25 ºC

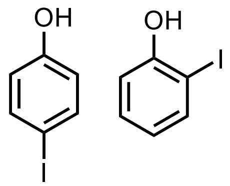

11:1

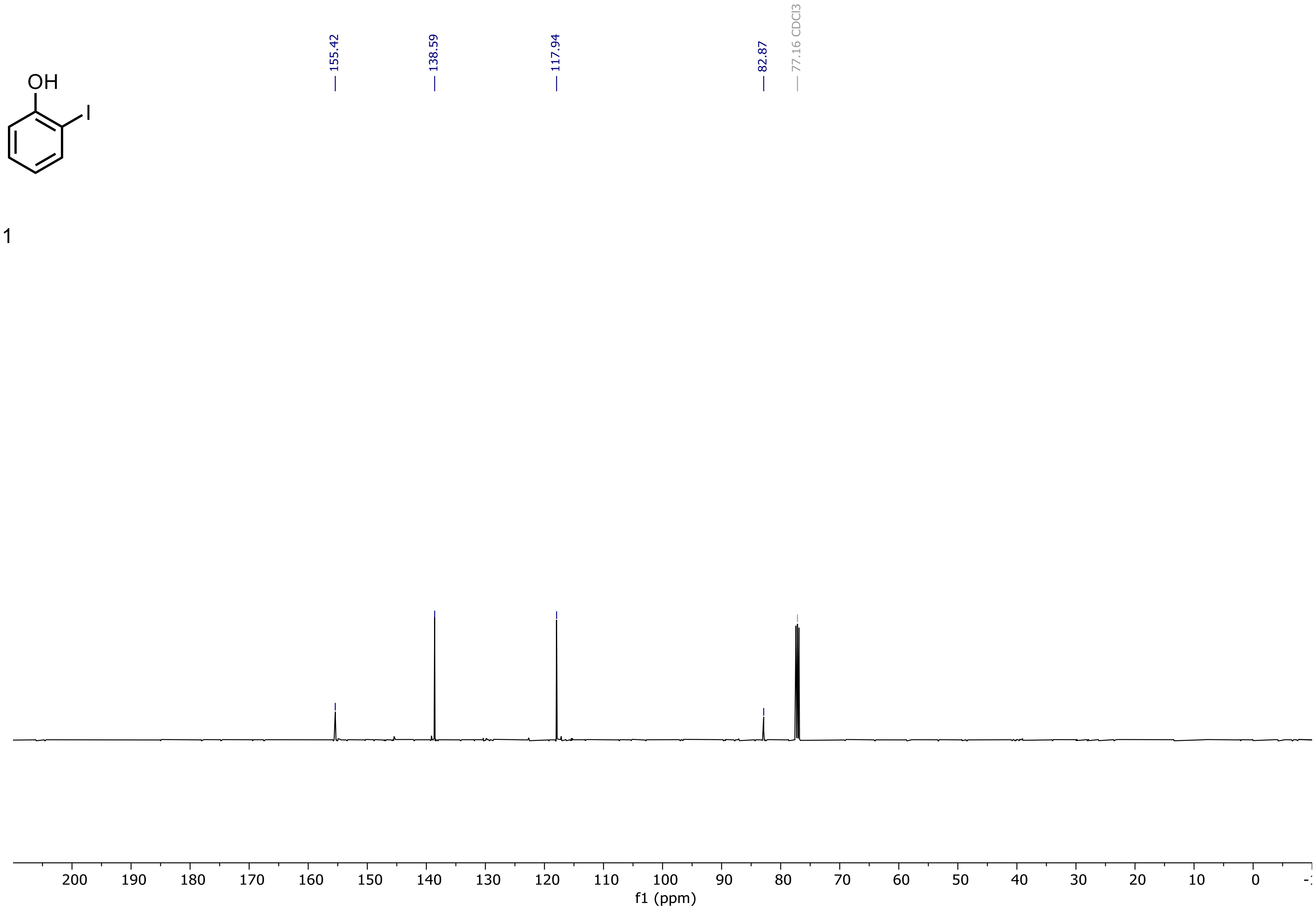

^{1}H NMR of 4-Iodoacetanilide (6)

$CDCl_3$, 300 MHz, 25 ºC

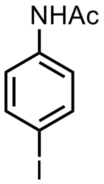

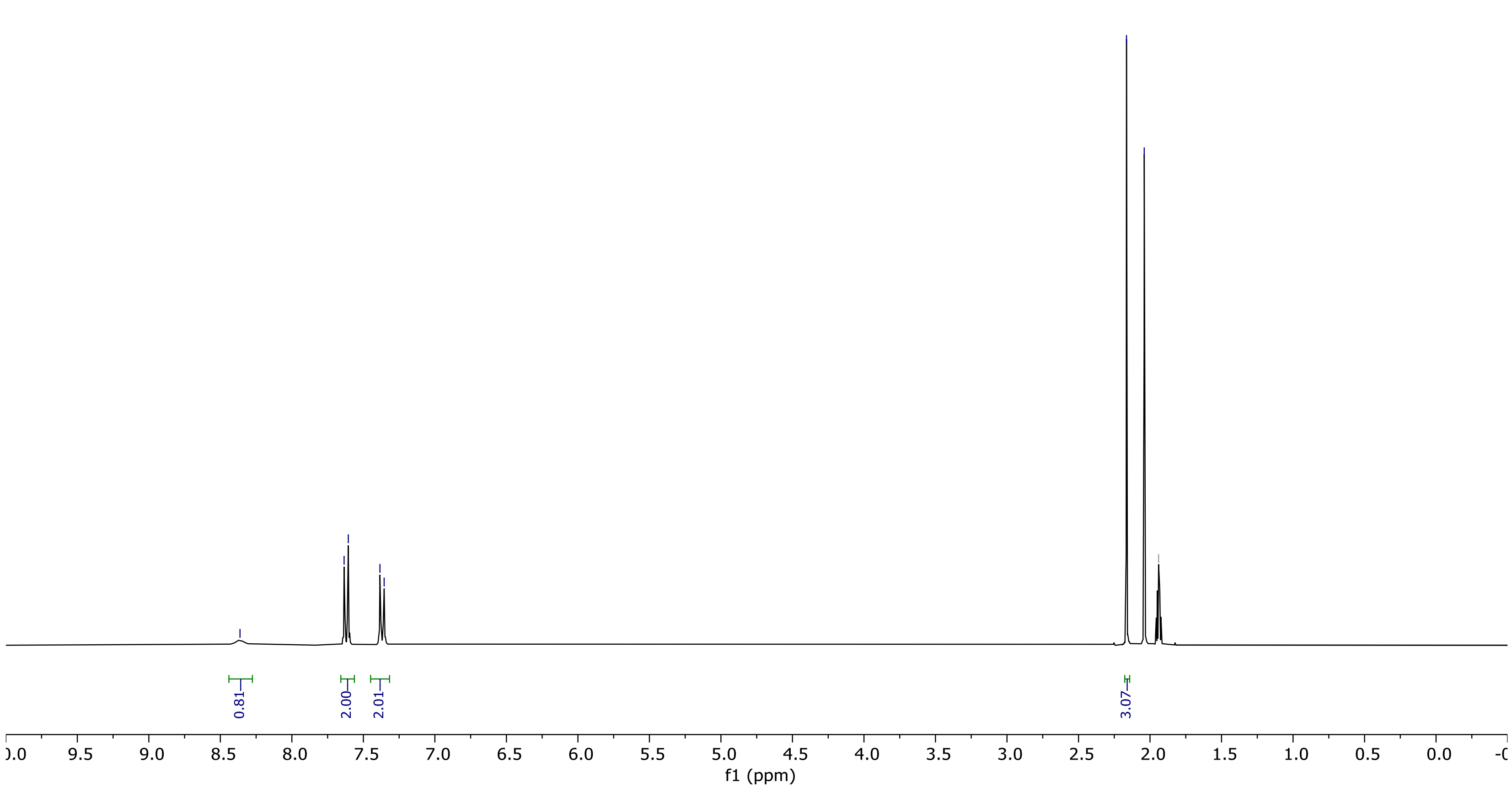

^{13}C NMR of 4-Iodoacetanilide (6)

$CDCl_3$, 75 MHz, 25 ºC

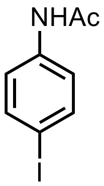

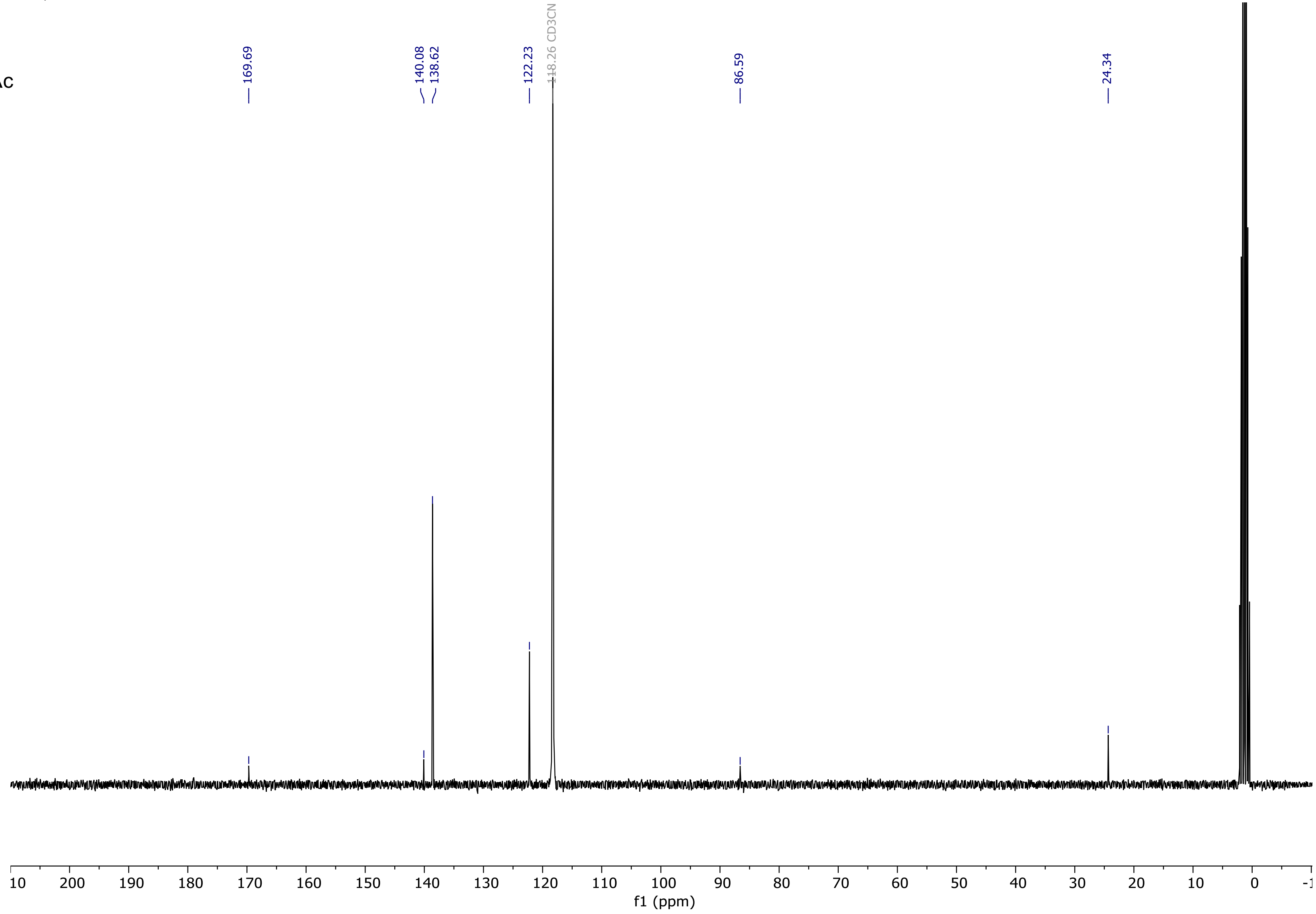

^{1}H NMR of 4-Iodofluorobenzene (7)

CD_2Cl_2, 500 MHz, 25 ºC

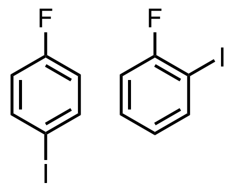

p/o:12/1

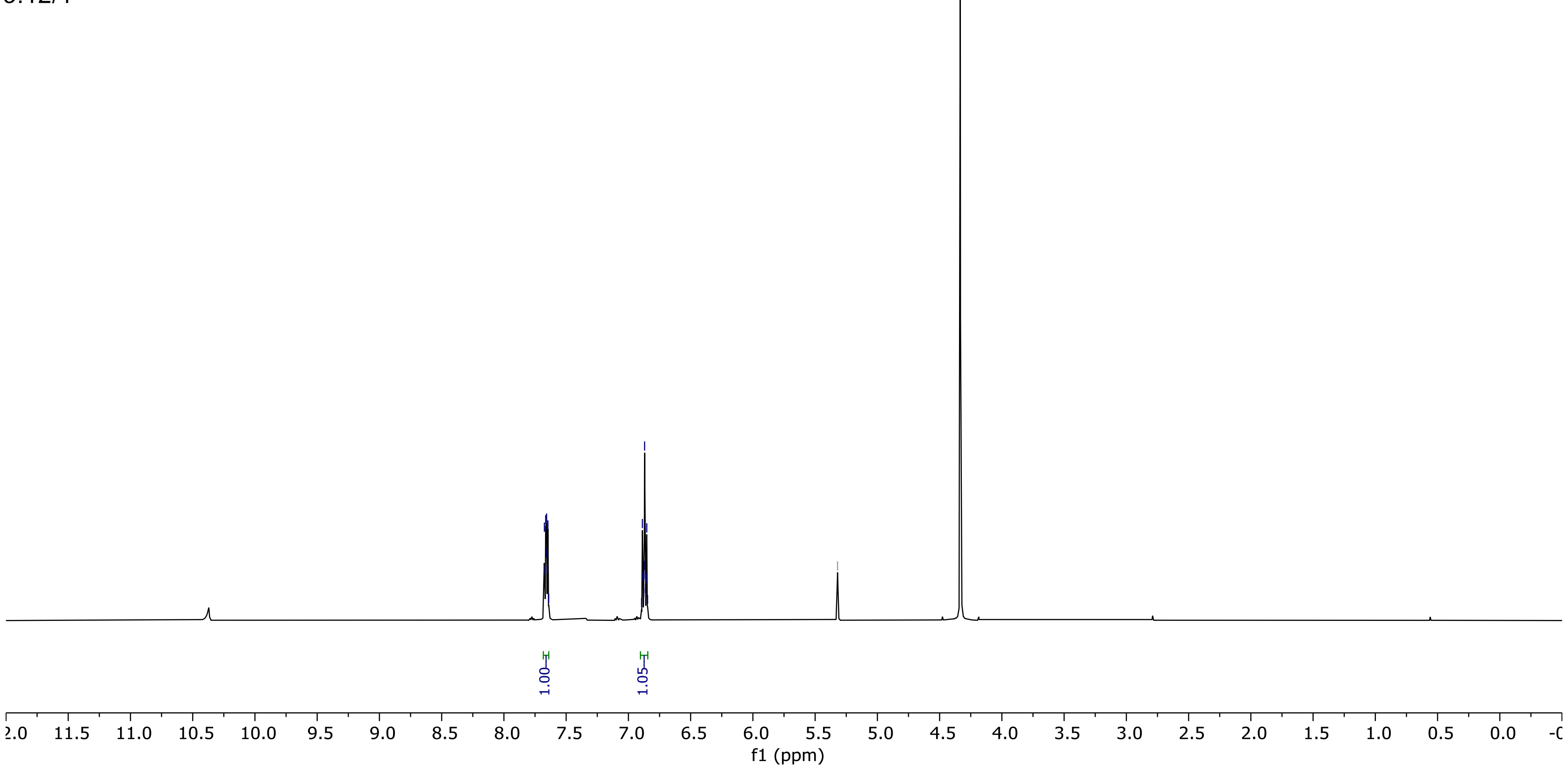

^{19}F NMR of 4-Iodofluorobenzene (7)

$CDCl_3$, 471 MHz, 25 ºC

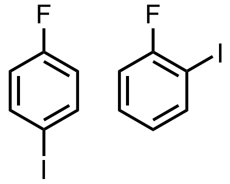

p/o:12/1

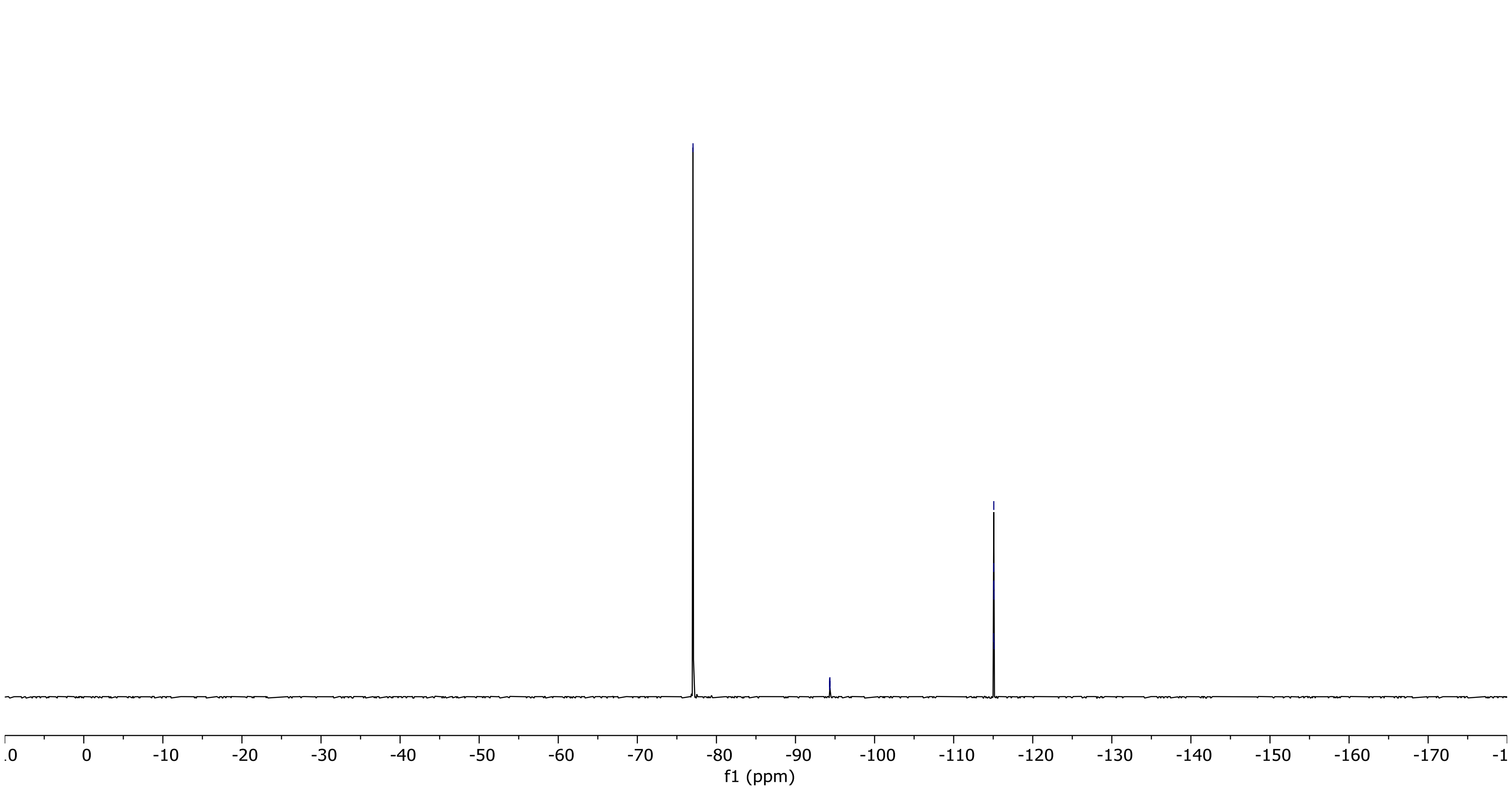

^{13}C NMR of 4-Iodofluorobenzene (7)

CD_3CN, 126 MHz, 25 ºC

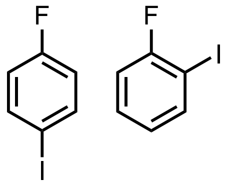

p/o:12/1

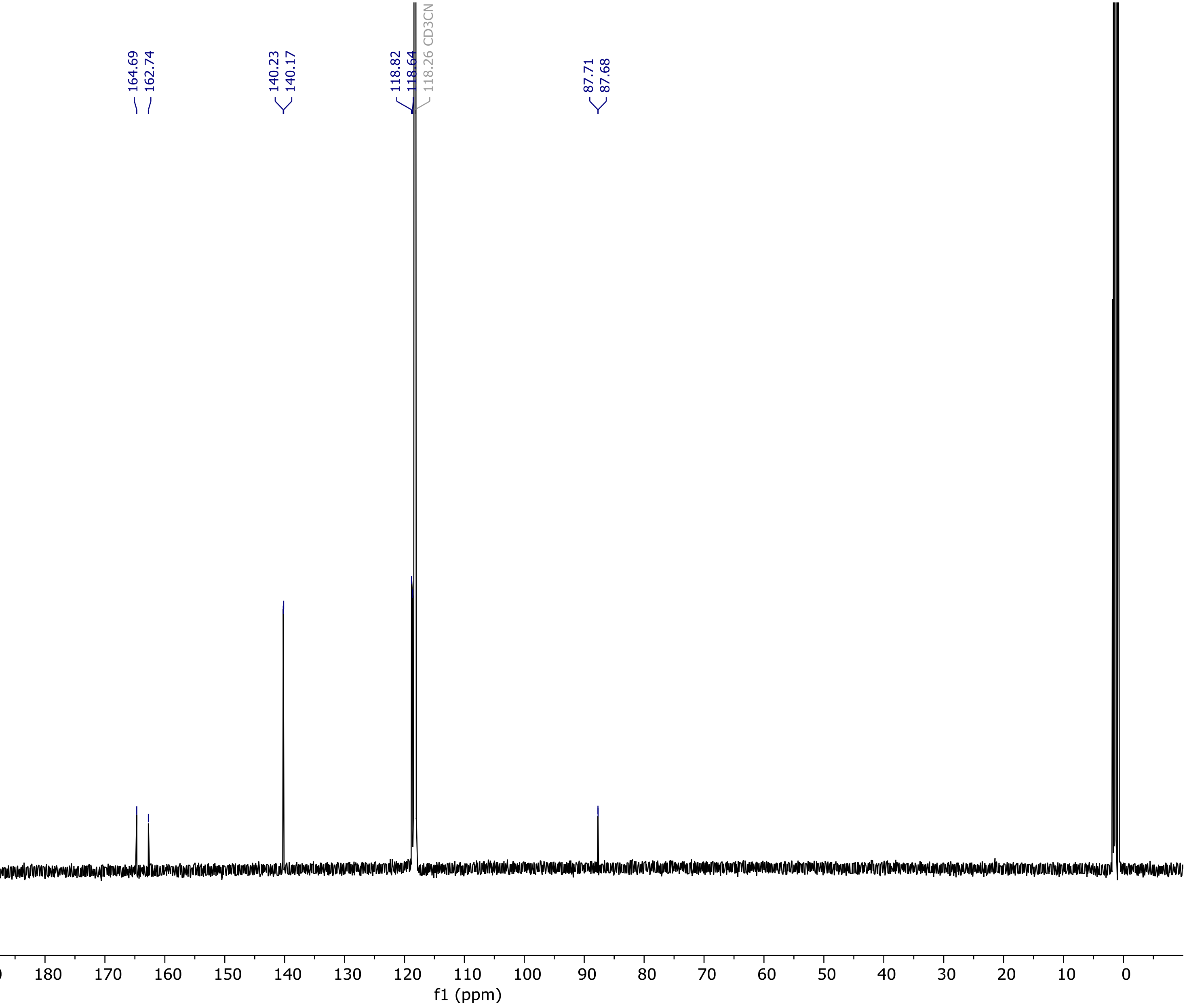

^{1}H NMR of 2-Acetyl-4-iodo-1-methylpyrrole (10)

$CDCl_3$, 500 MHz, 25 ºC

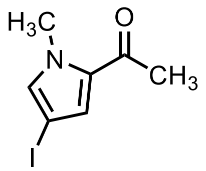

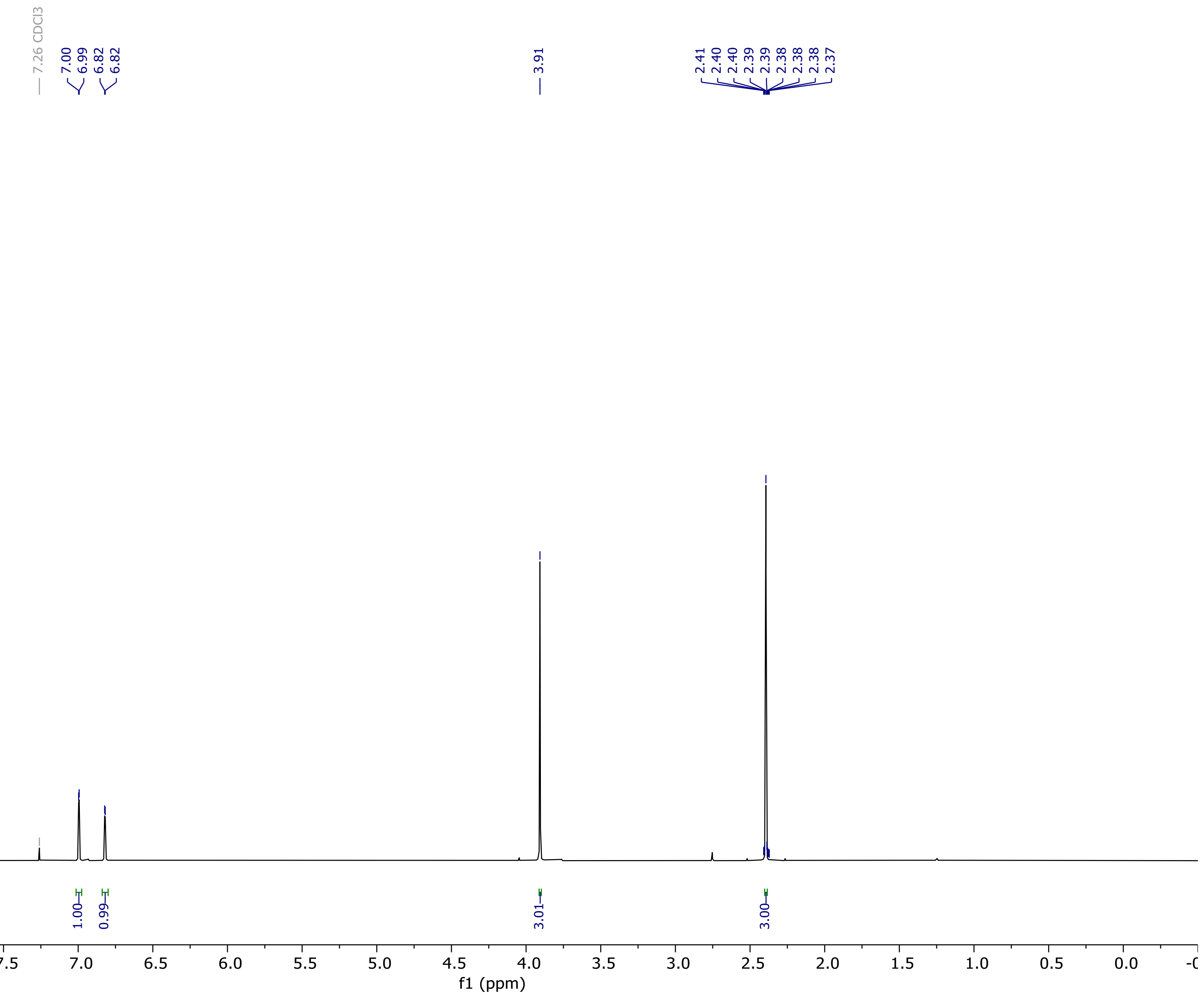

^{13}C NMR of 2-Acetyl-4-iodo-1-methylpyrrole (10)

$CDCl_3$, 126 MHz, 23 ºC

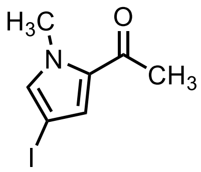

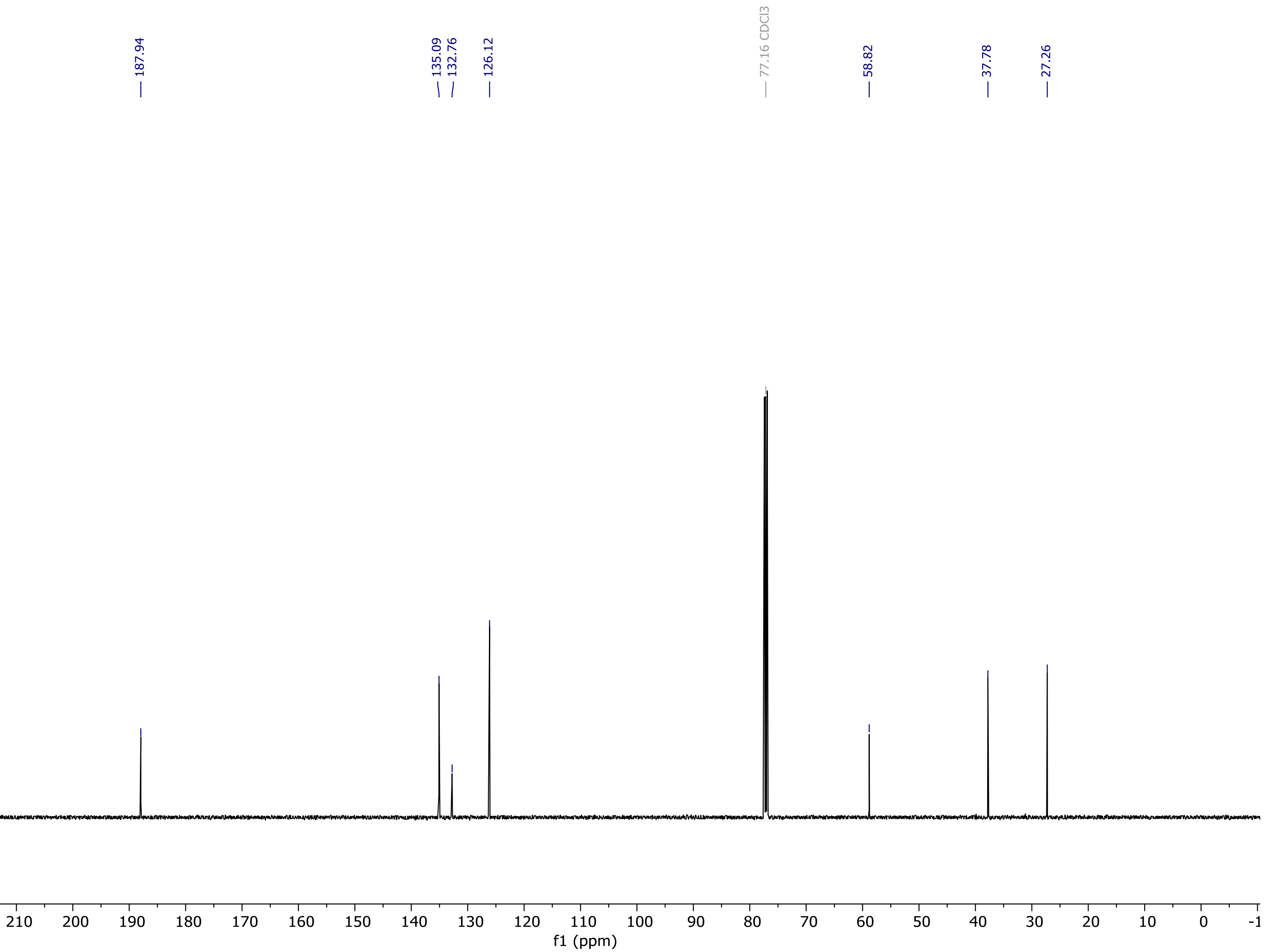

^{1}H NMR of 3-Iodo-2,6-dimethoxypyridine (11)

$CDCl_3$, 500 MHz, 25 ºC

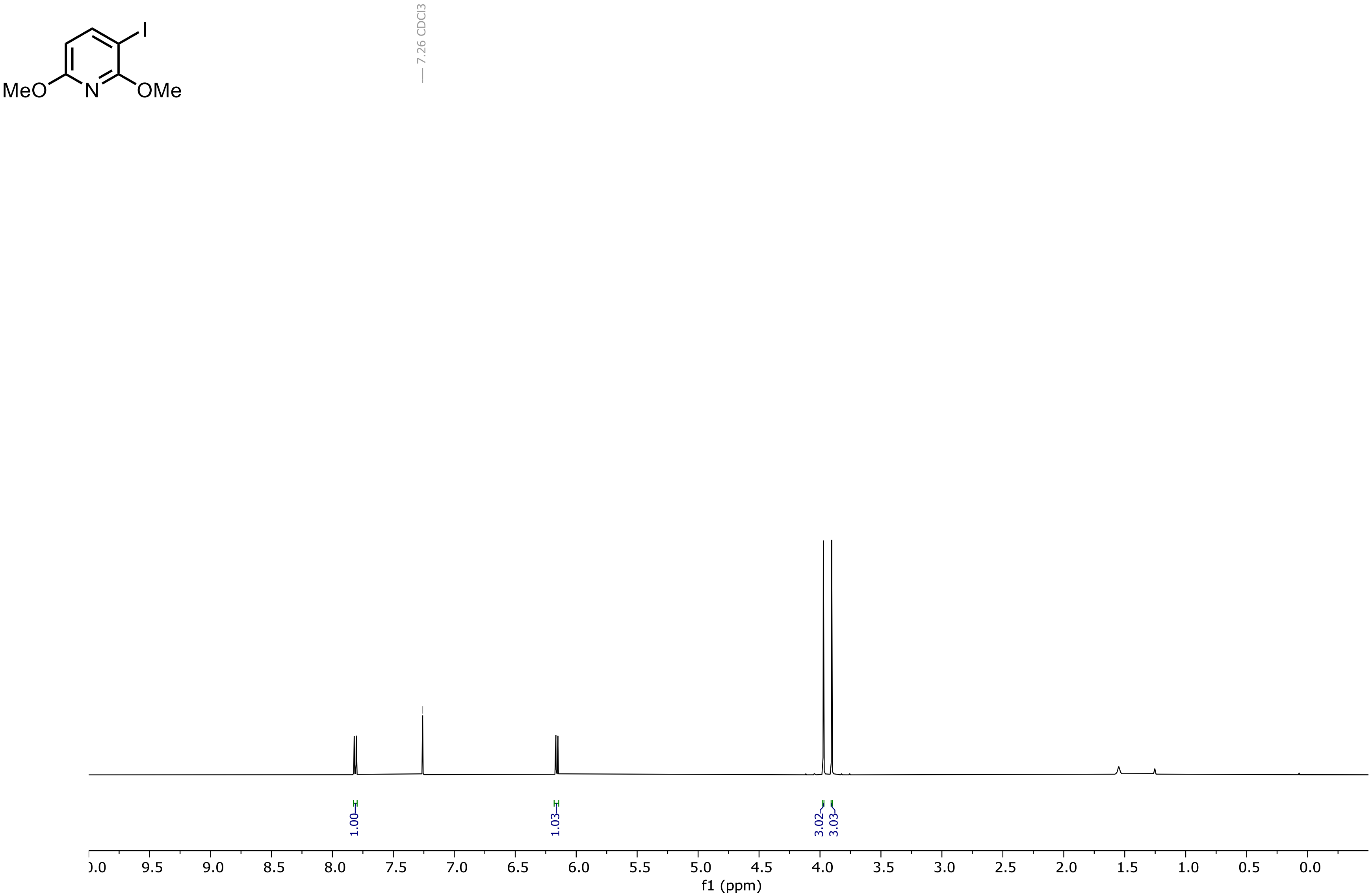

^{13}C NMR of 3-Iodo-2,6-dimethoxypyridine (11)

$CDCl_3$, 126 MHz, 23 ºC

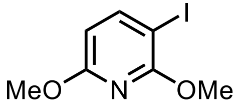

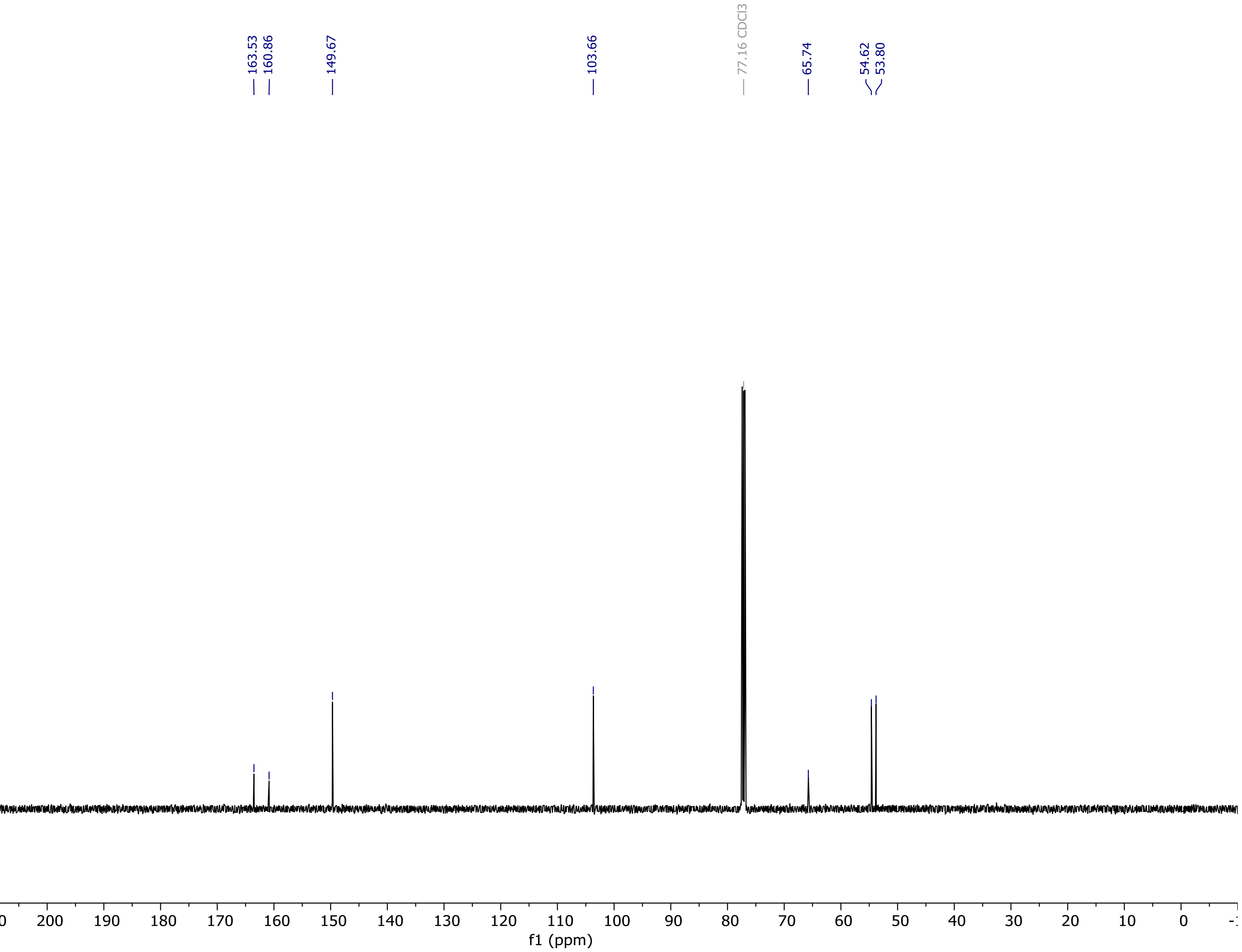

^{1}H NMR of 3-Iodoazaindazole (12)

$(CD_3)_2SO$, 600 MHz, 25 ºC

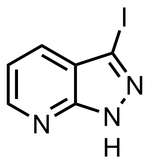

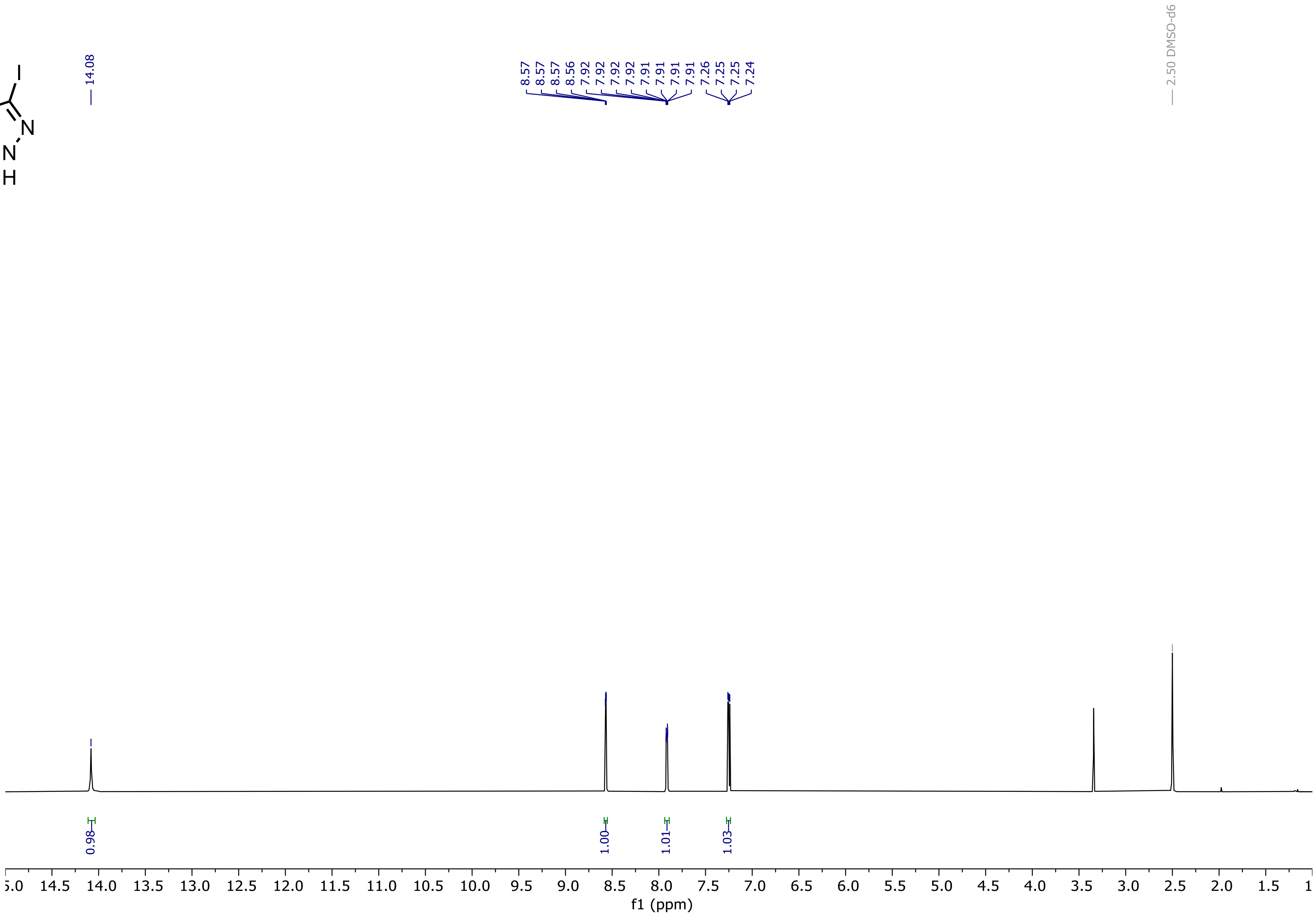

^{13}C NMR of 3-Iodoazaindazole (12)

$(CD_3)_2SO$, 151 MHz, 25 ºC

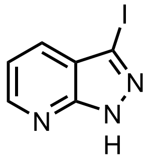

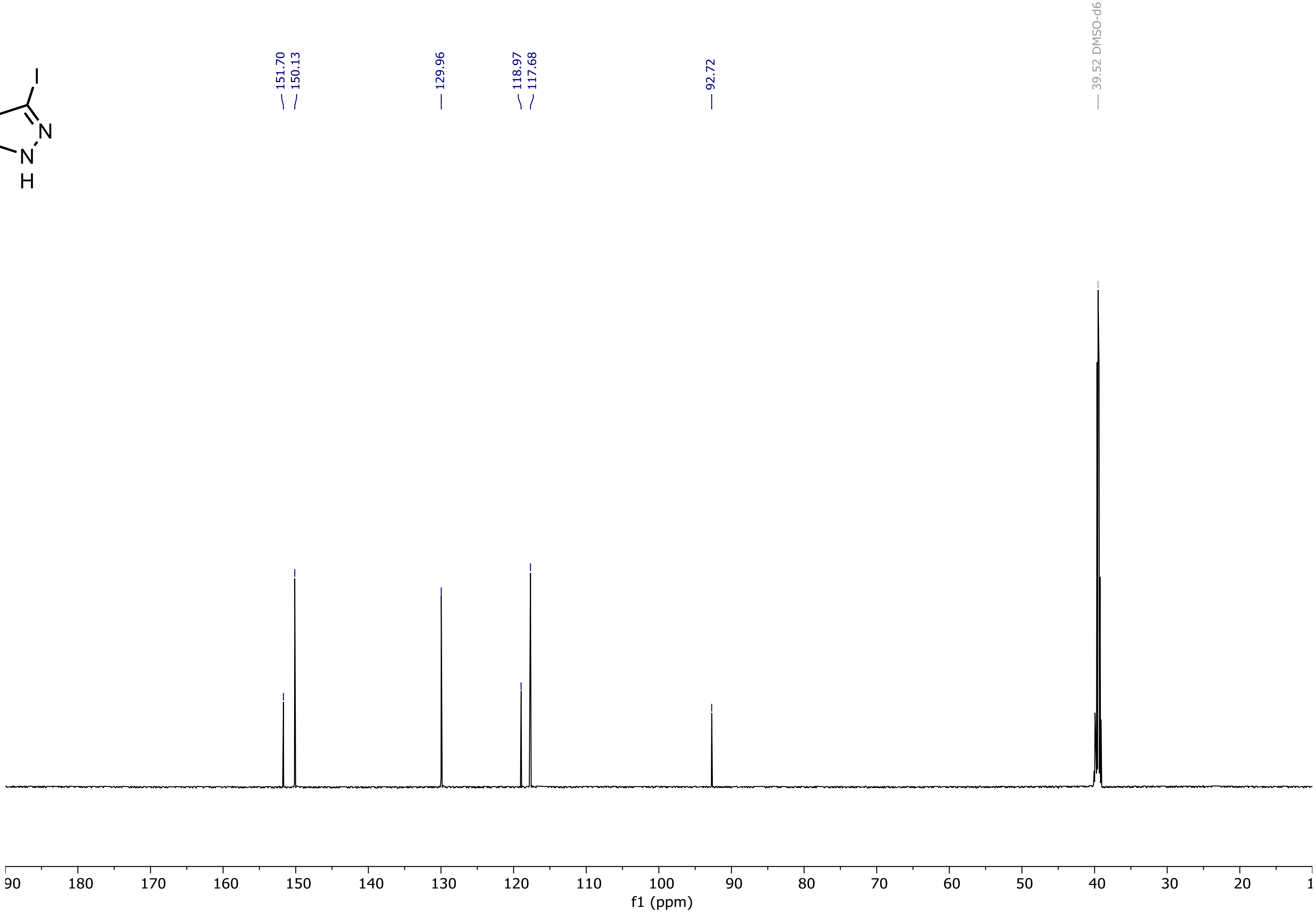

^{1}H NMR of 4-Iodopyrazole-3-carboxylate (13)

CD_3CN, 500 MHz, 25 ºC

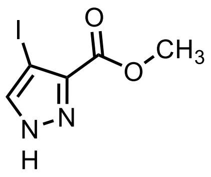

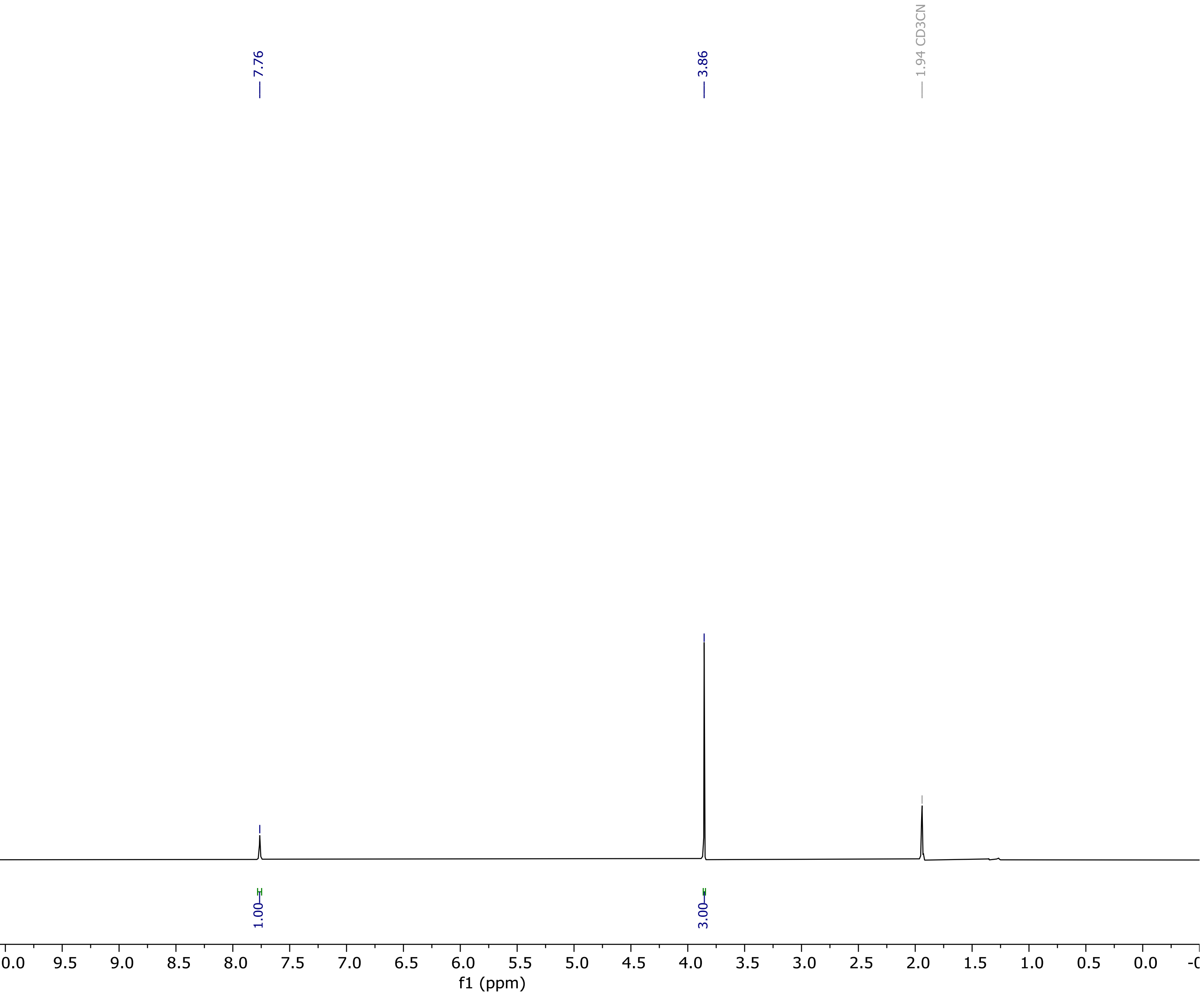

^{13}C NMR of 4-Iodopyrazole-3-carboxylate (13)

CD_3OD, 151 MHz, 25 ºC

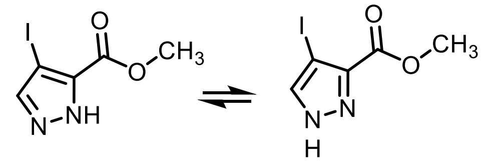

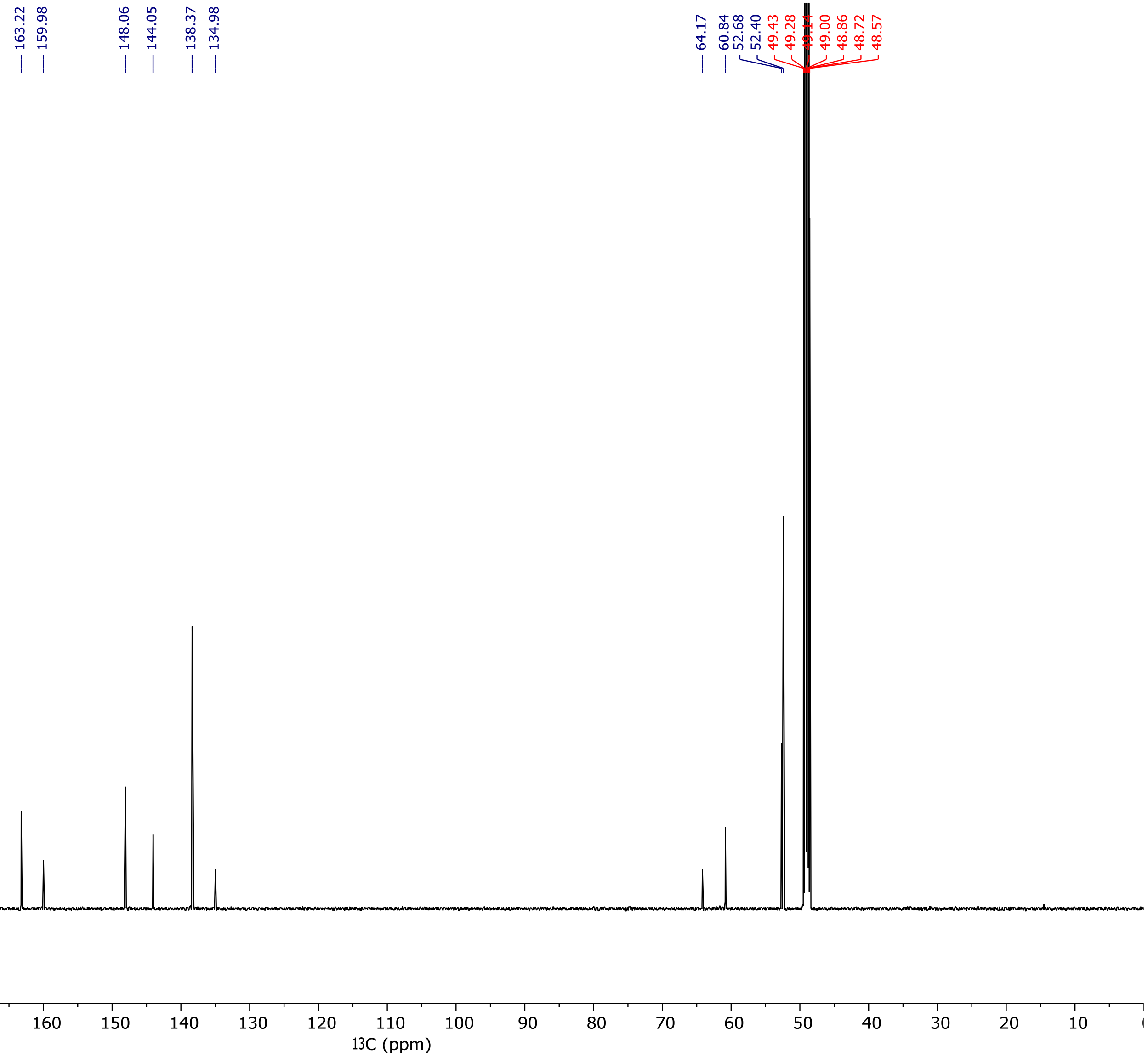

^{1}H NMR of 2-Iodo-5-bromothiazole (14)

$(CD_3)_2SO$, 300 MHz, 25 ºC

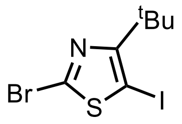

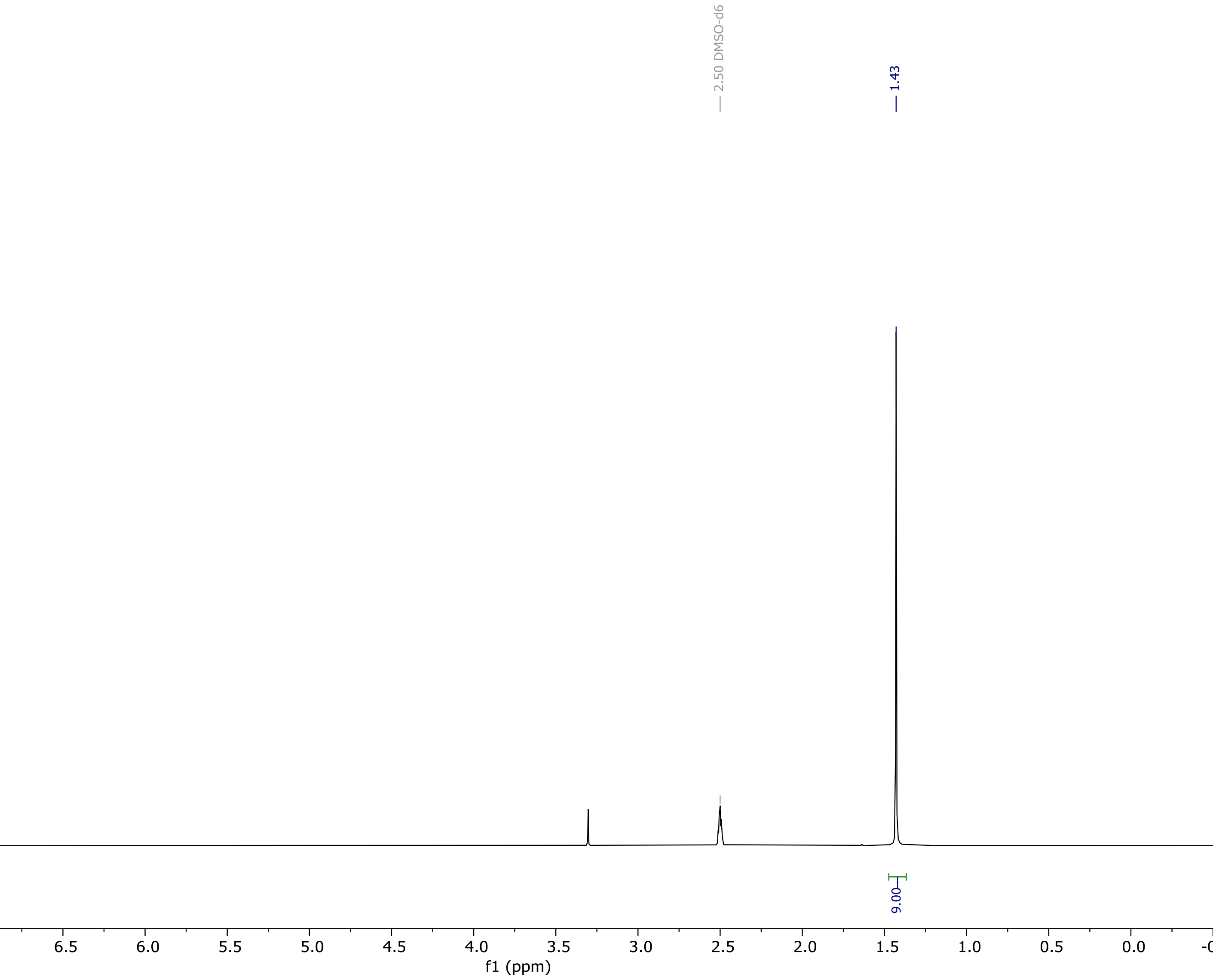

^{13}C NMR of 2-Iodo-5-bromothiazole (14)

$(CD_3)_2SO$, 75 MHz, 25 ºC

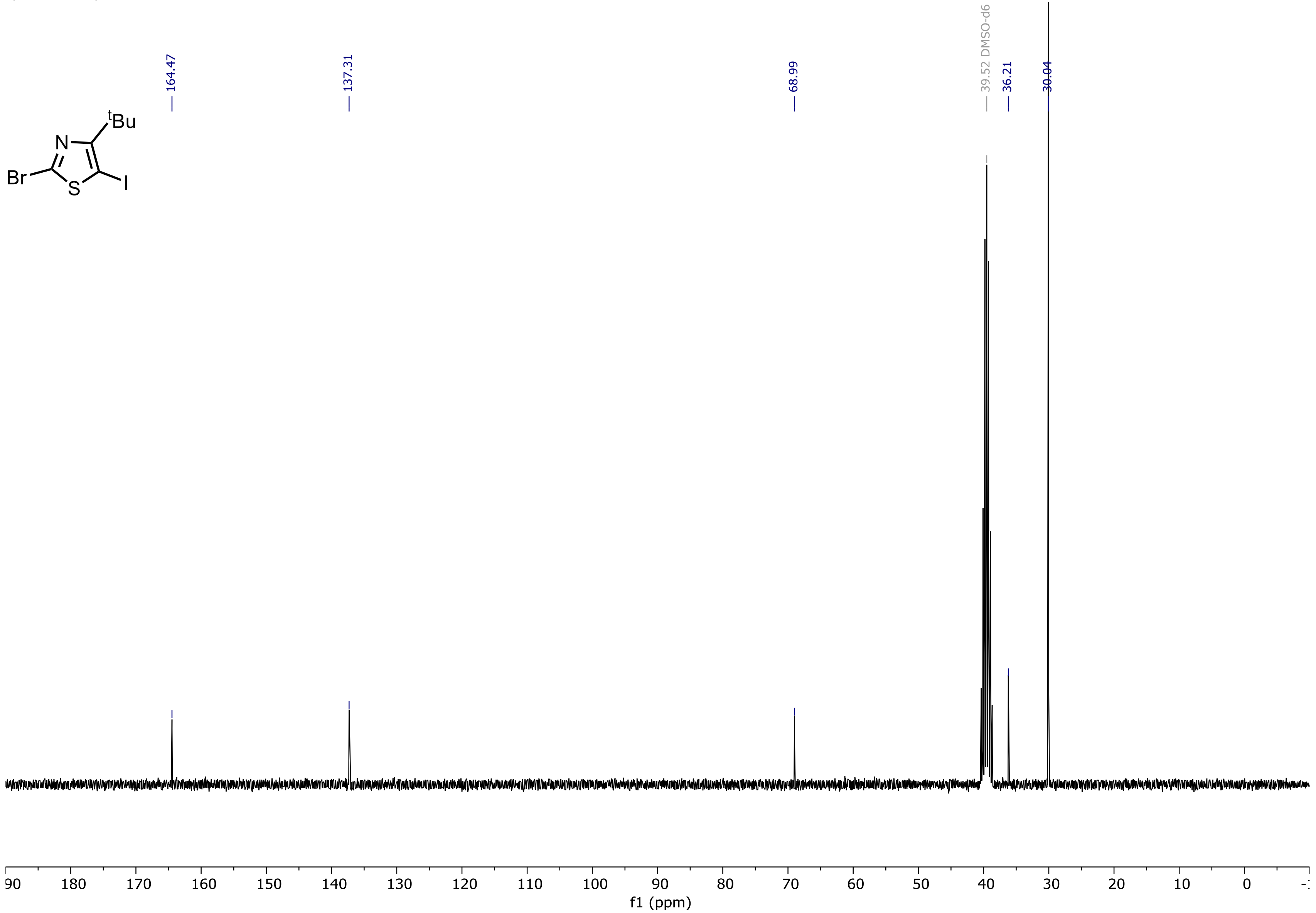

^{1}H NMR of 6-Chloro-7-iododezapurine (15)

$(CD_3)_2SO$, 500 MHz, 25 ºC

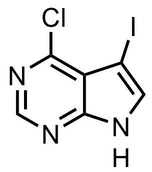

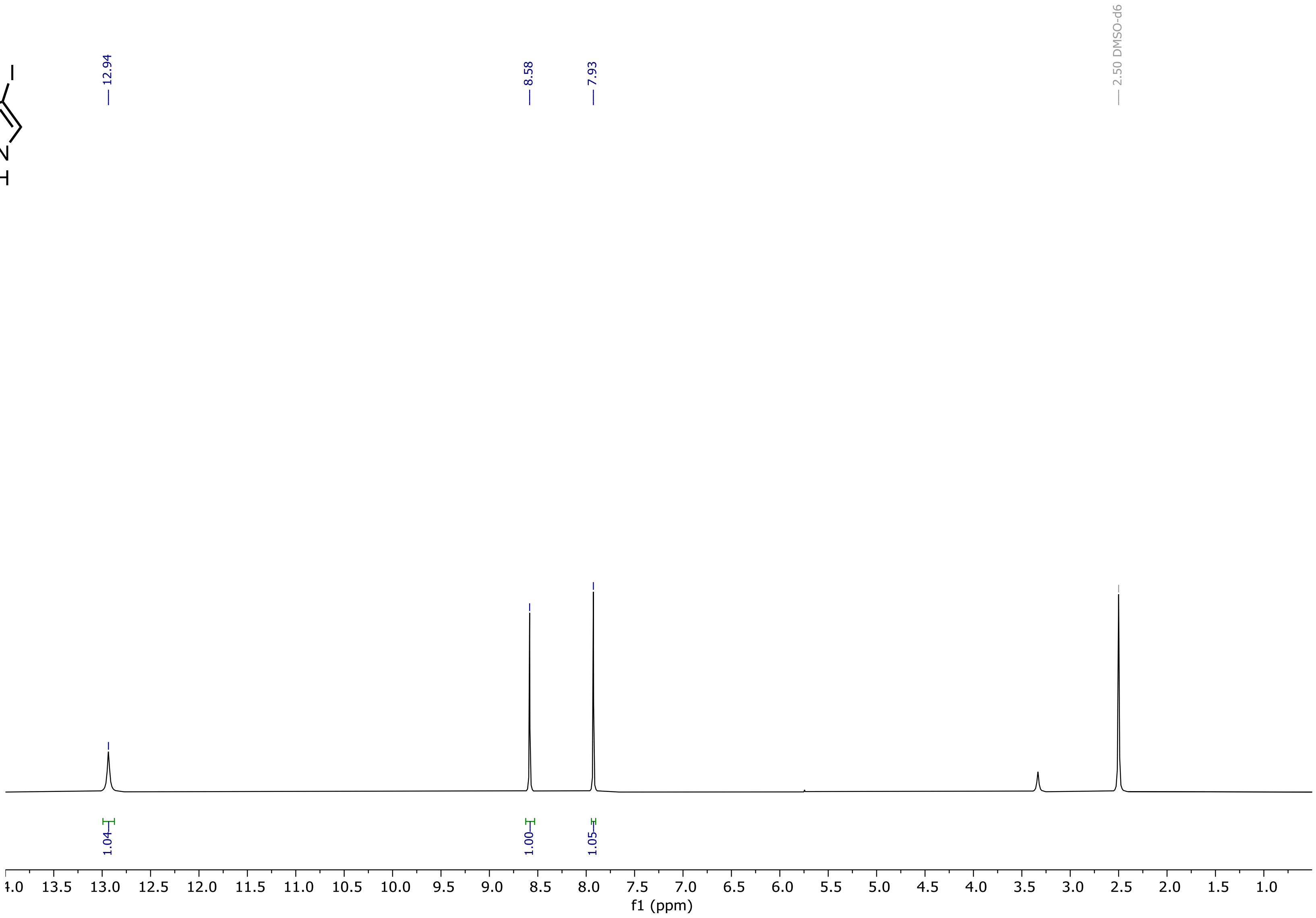

^{13}C NMR of 6-Chloro-7-iododezapurine (15)

$(CD_3)_2SO$, 126 MHz, 25 ºC

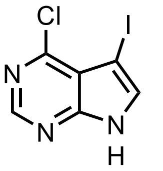

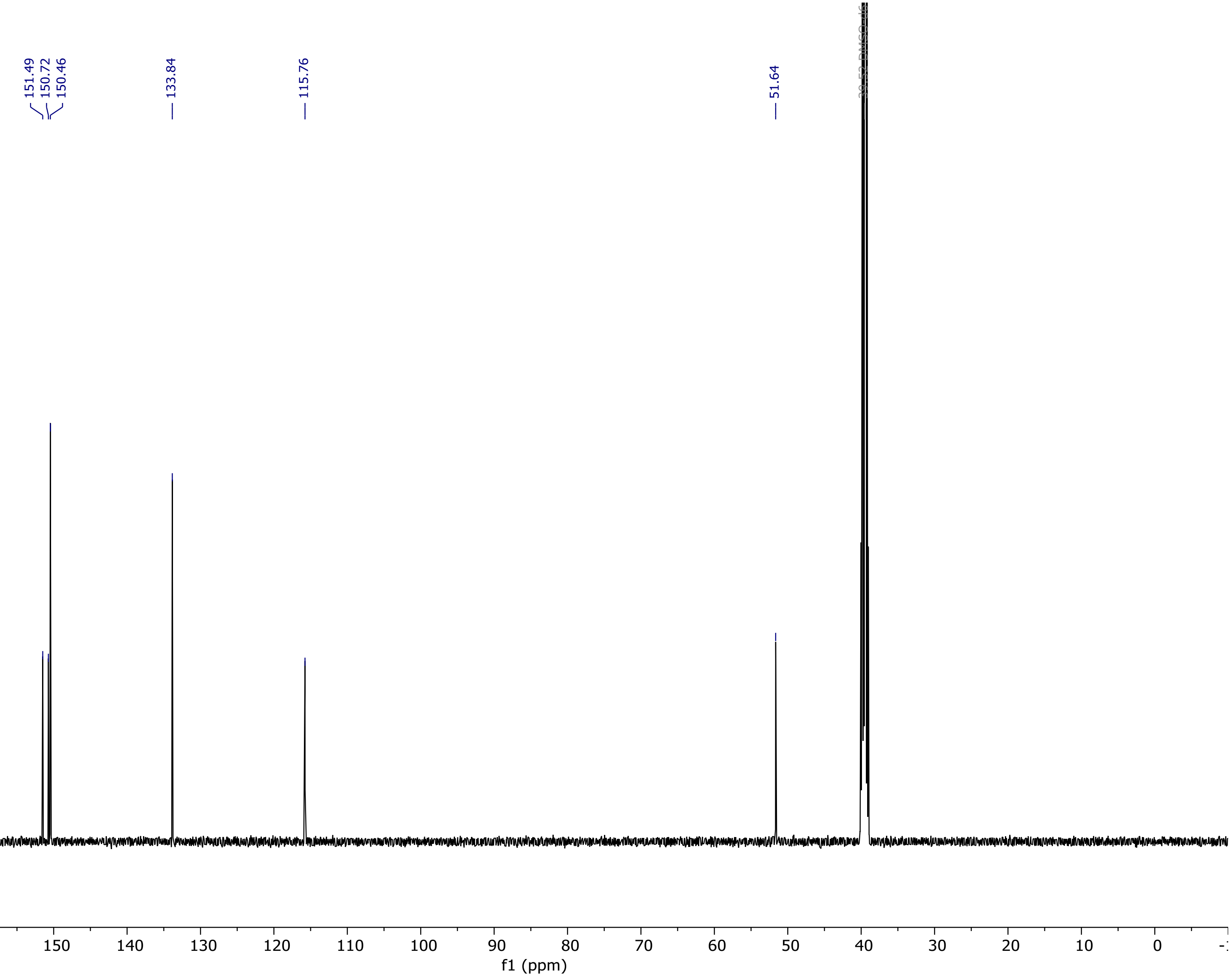

^{1}H NMR of 2-(5-Iodothiophene)-benzothiazole (16)

$CDCl_3$, 500 MHz, 25 ºC

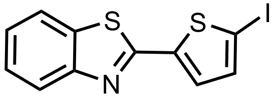

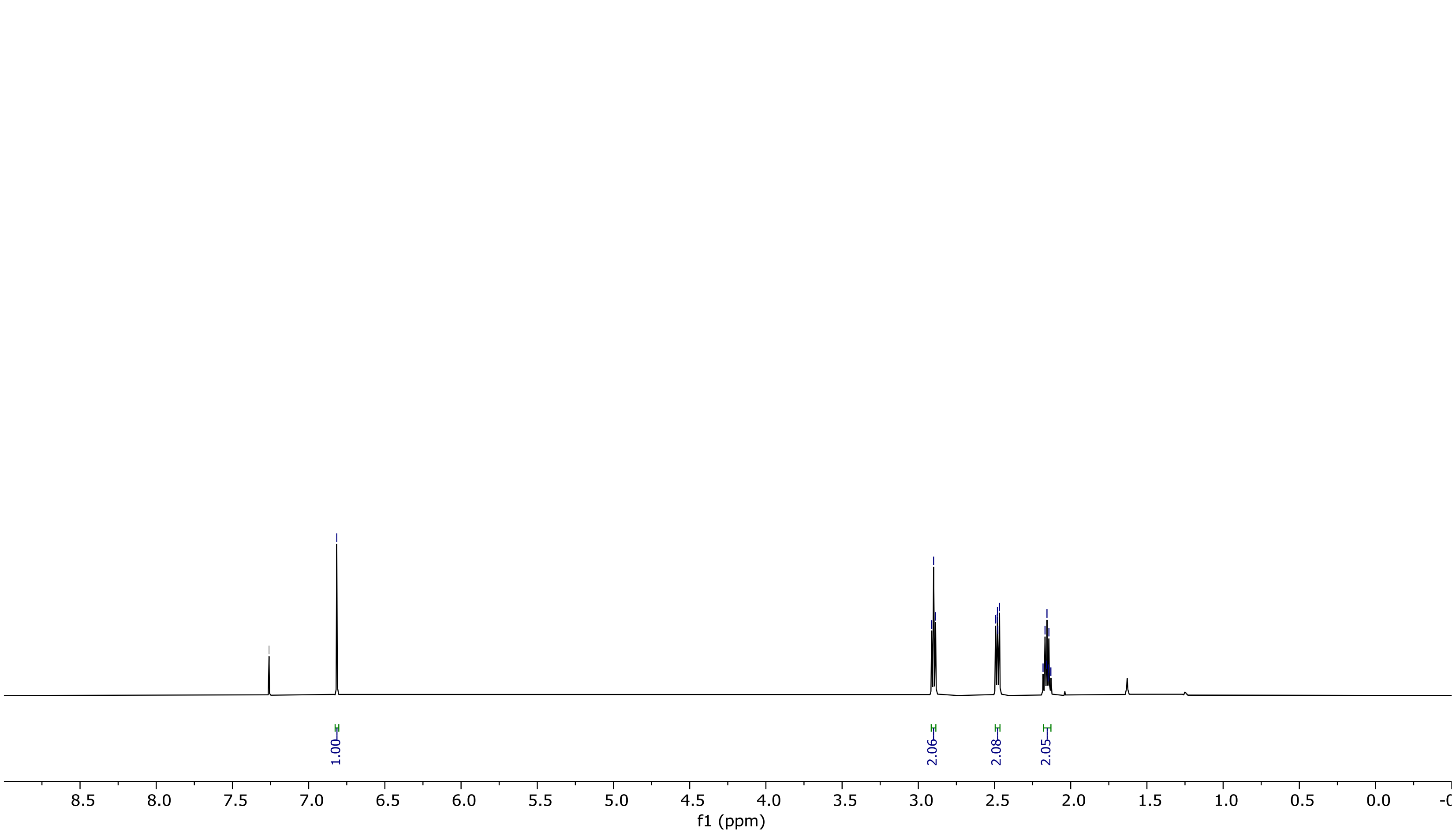

^{13}C NMR of 2-(5-Iodothiophene)-benzothiazole (16)

$CDCl_3$, 126 MHz, 25 ºC

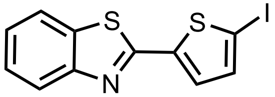

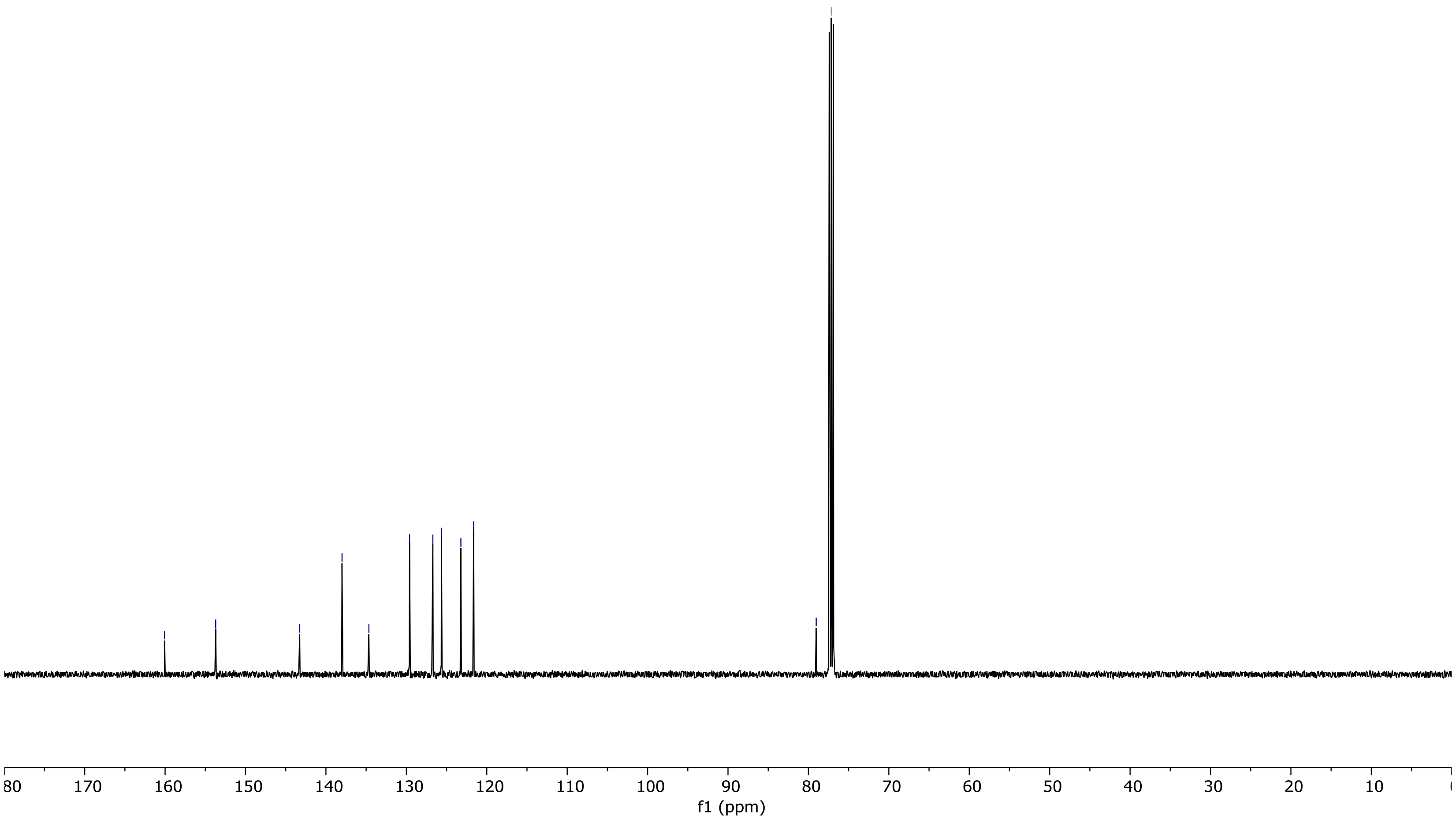

^{1}H NMR of 4-Iodopyrazol-1-benzoic acid (17)

$(CD_3)_2SO$, 300 MHz, 25 ºC

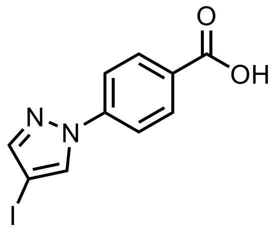

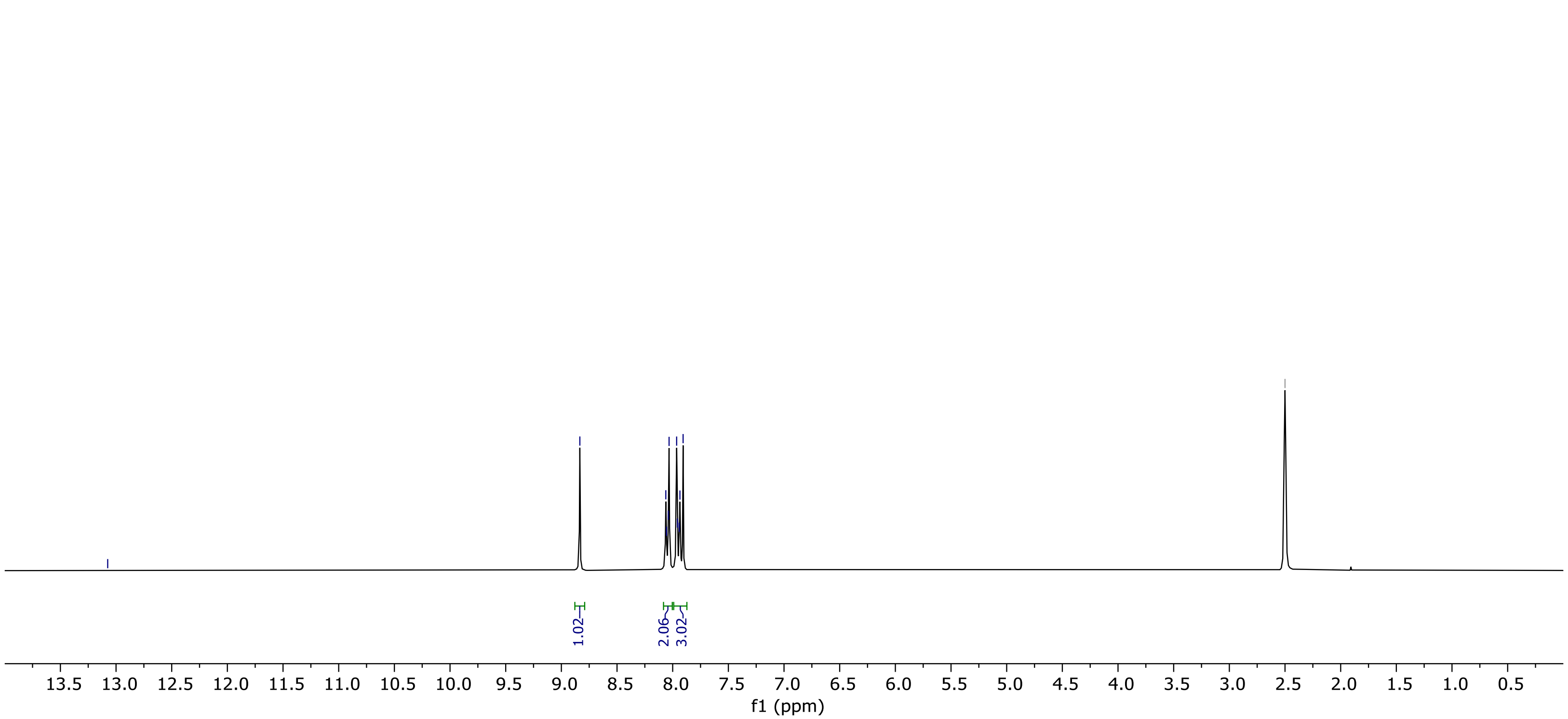

^{13}C NMR of 4-Iodopyrazol-1-benzoic acid (17)

$(CD_3)_2SO$, 75 MHz, 25 ºC

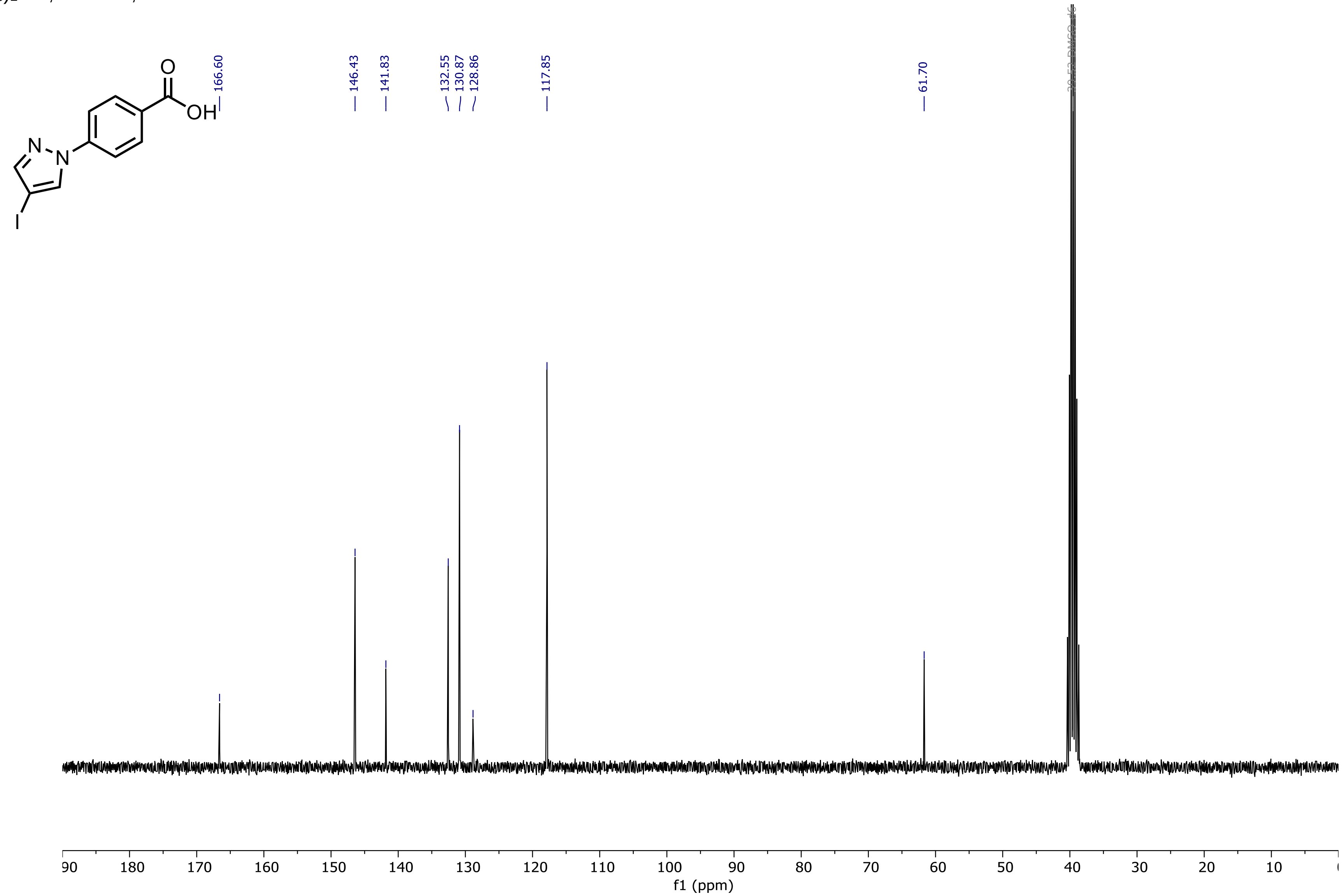

^{1}H NMR of Iodinated-ipriflavone (18)

$CDCl_3$, 500 MHz, 25 ºC

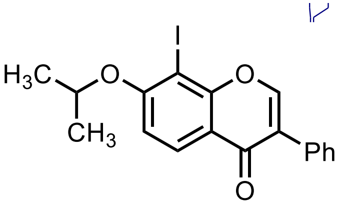

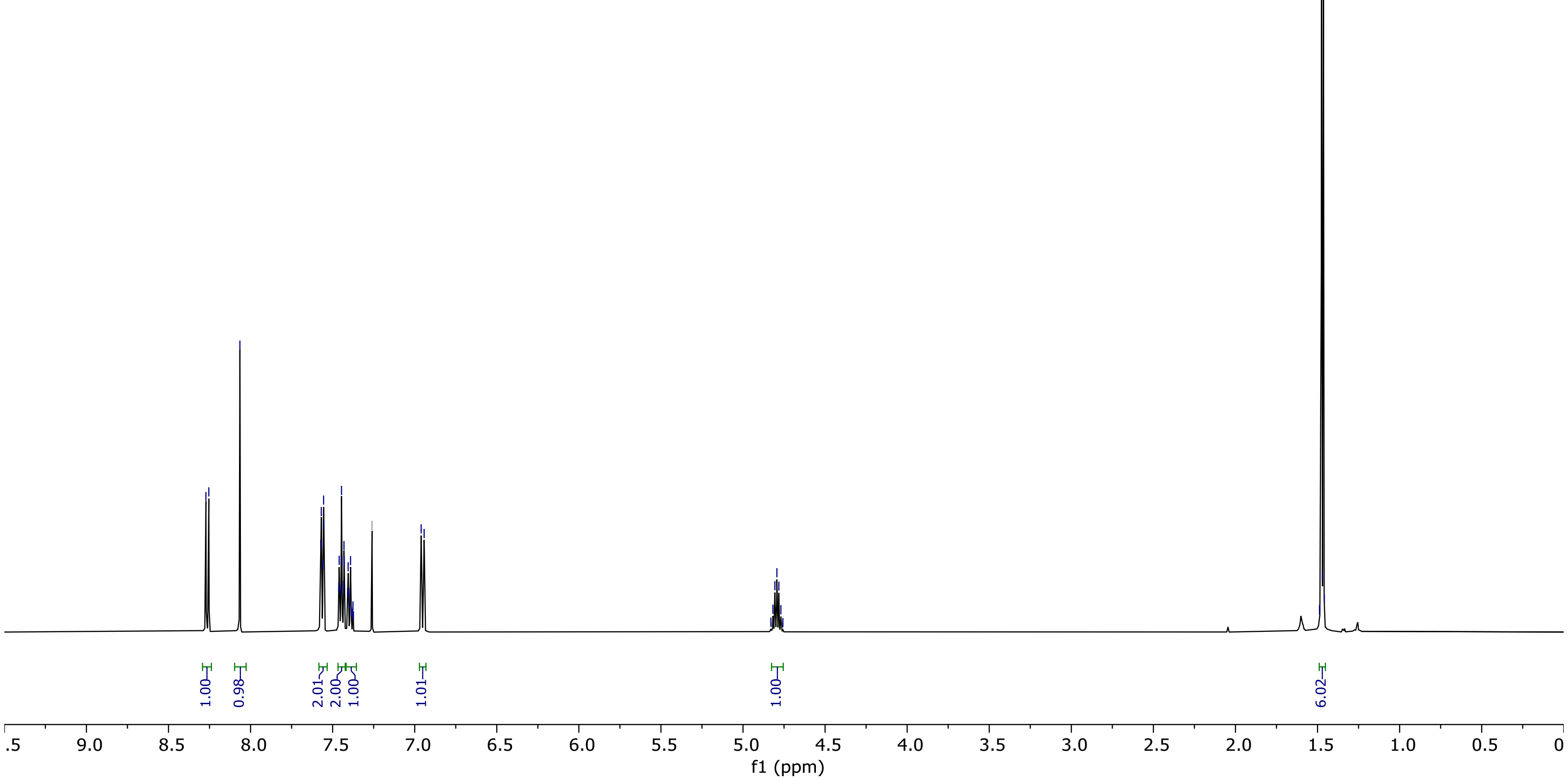

^{13}C NMR of Iodinated-ipriflavone (18)

$CDCl_3$, 126 MHz, 25 ºC

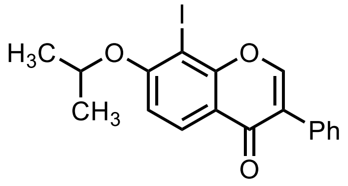

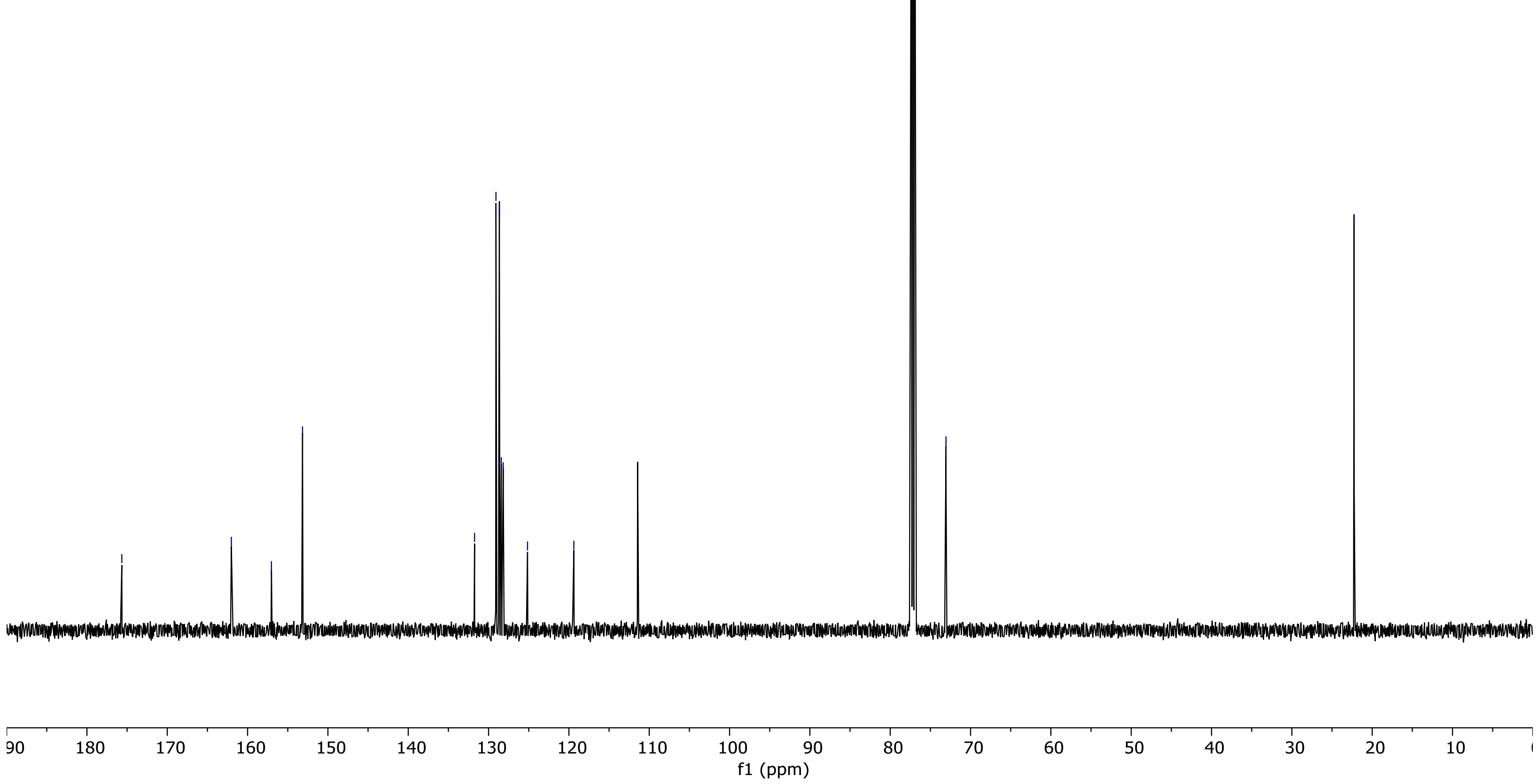

^{1}H NMR of Iodinated-coumarin1 (19)

CDCl$_3$, 500 MHz, 25 ºC

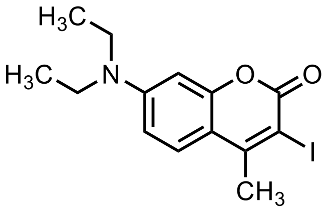

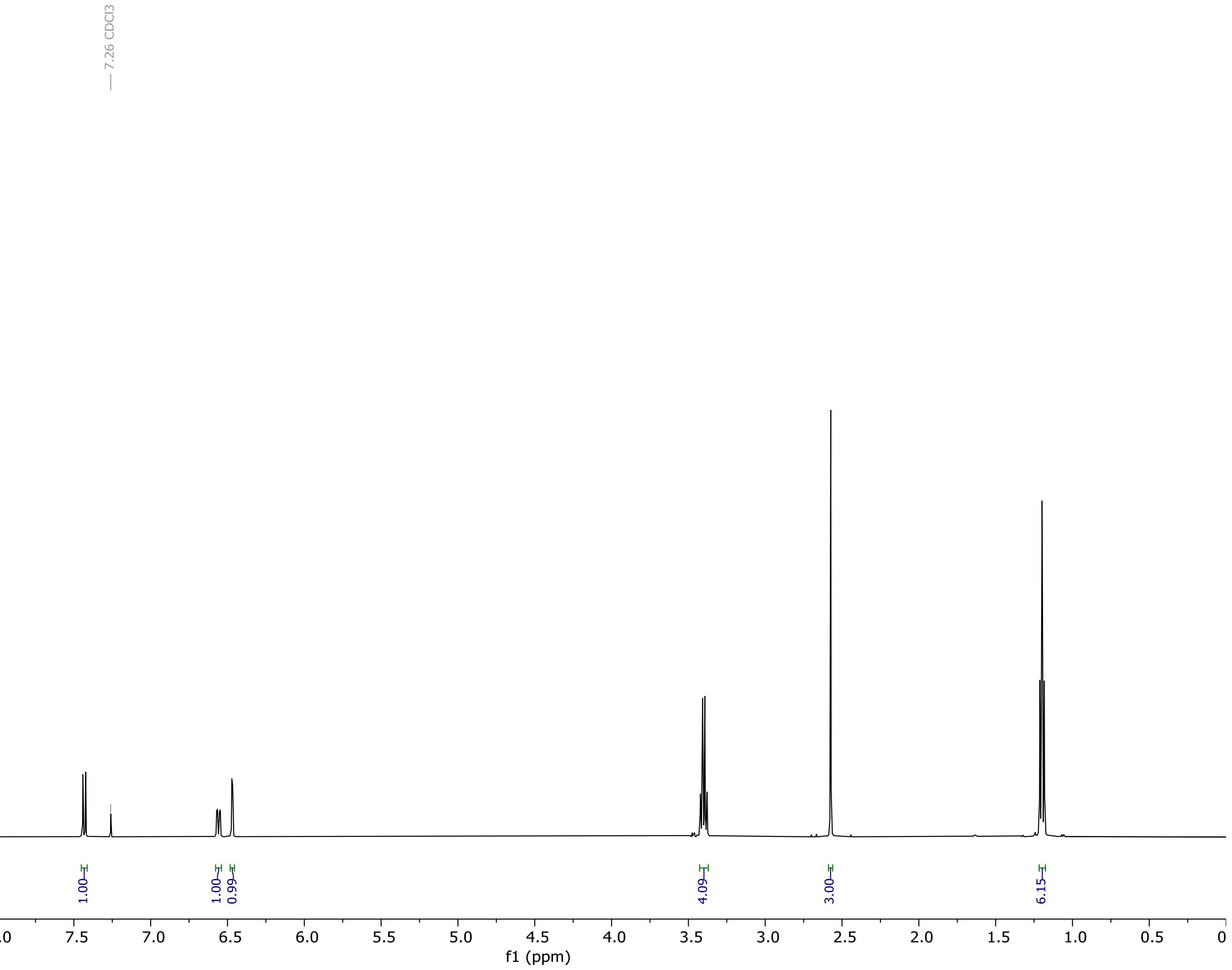

^{13}C NMR of Iodinated-coumarin1 (19)

$CDCl_3$, 126 MHz, 25 ºC

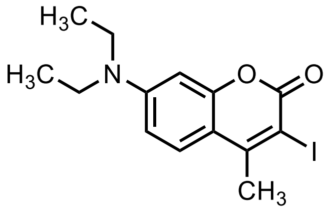

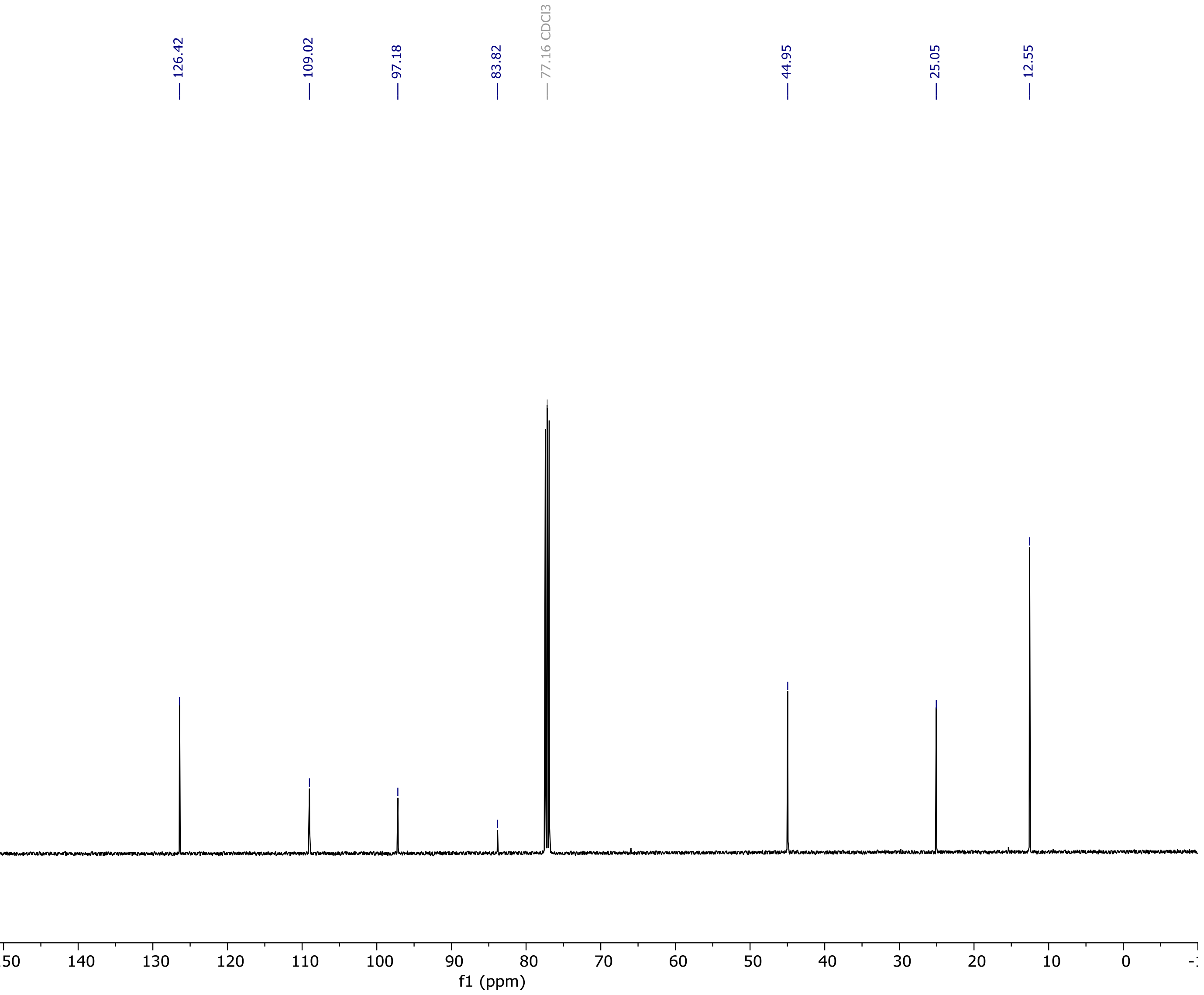

^{1}H NMR of Iodinated-procymidone (20)

$CDCl_3$, 500 MHz, 25 ºC

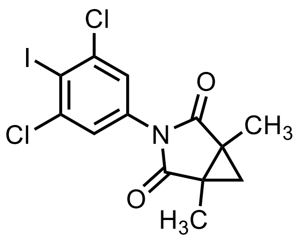

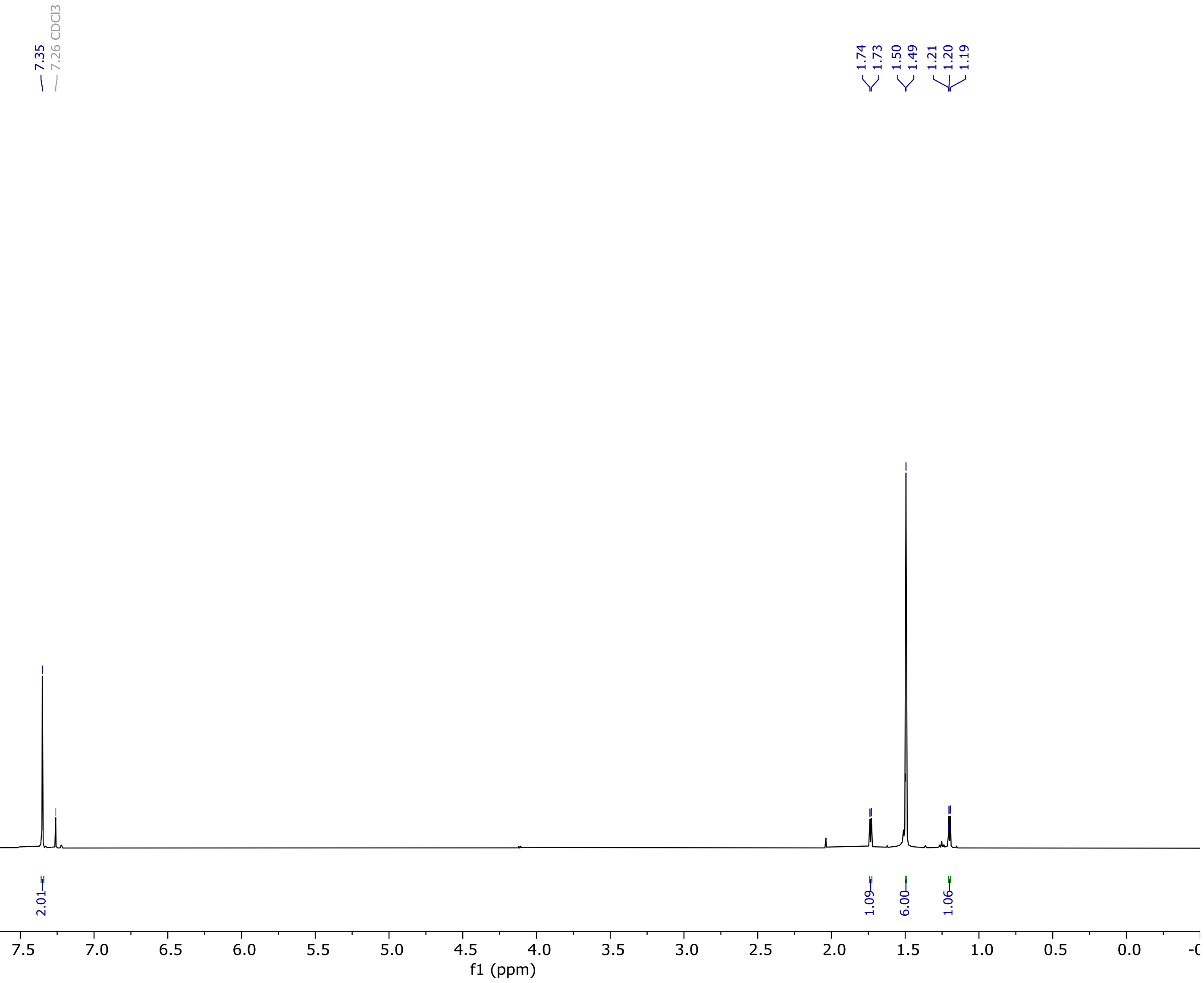

^{13}C NMR of Iodinated-procymidone (20)

$CDCl_3$, 126 MHz, 25 ºC

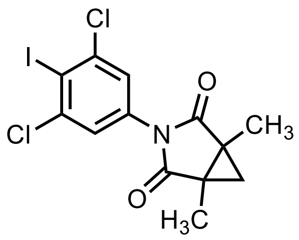

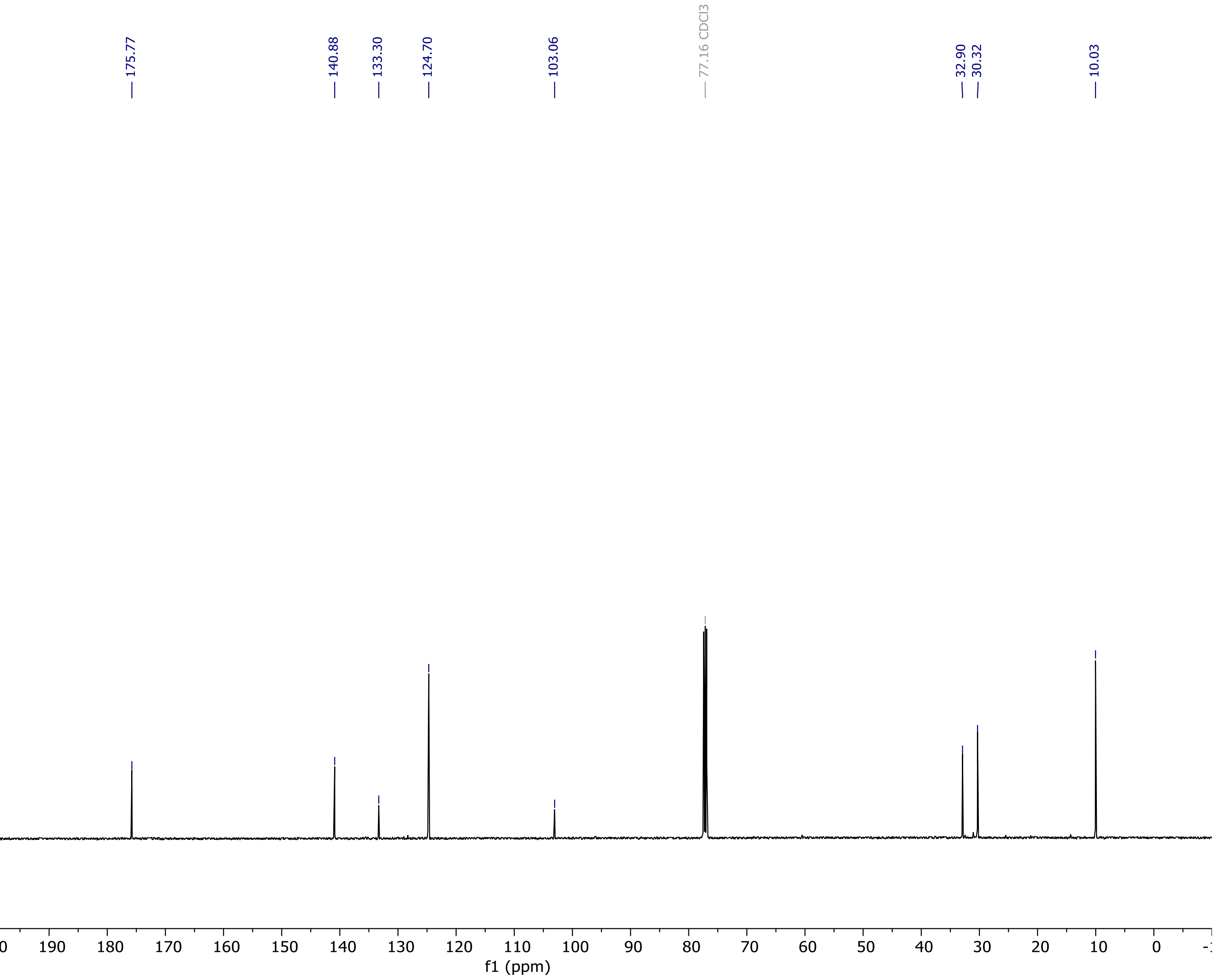

^{1}H NMR of Iodinated-boscalid triflate salt (21)

$(CD_3)_2SO$, 500 MHz, 25 ºC

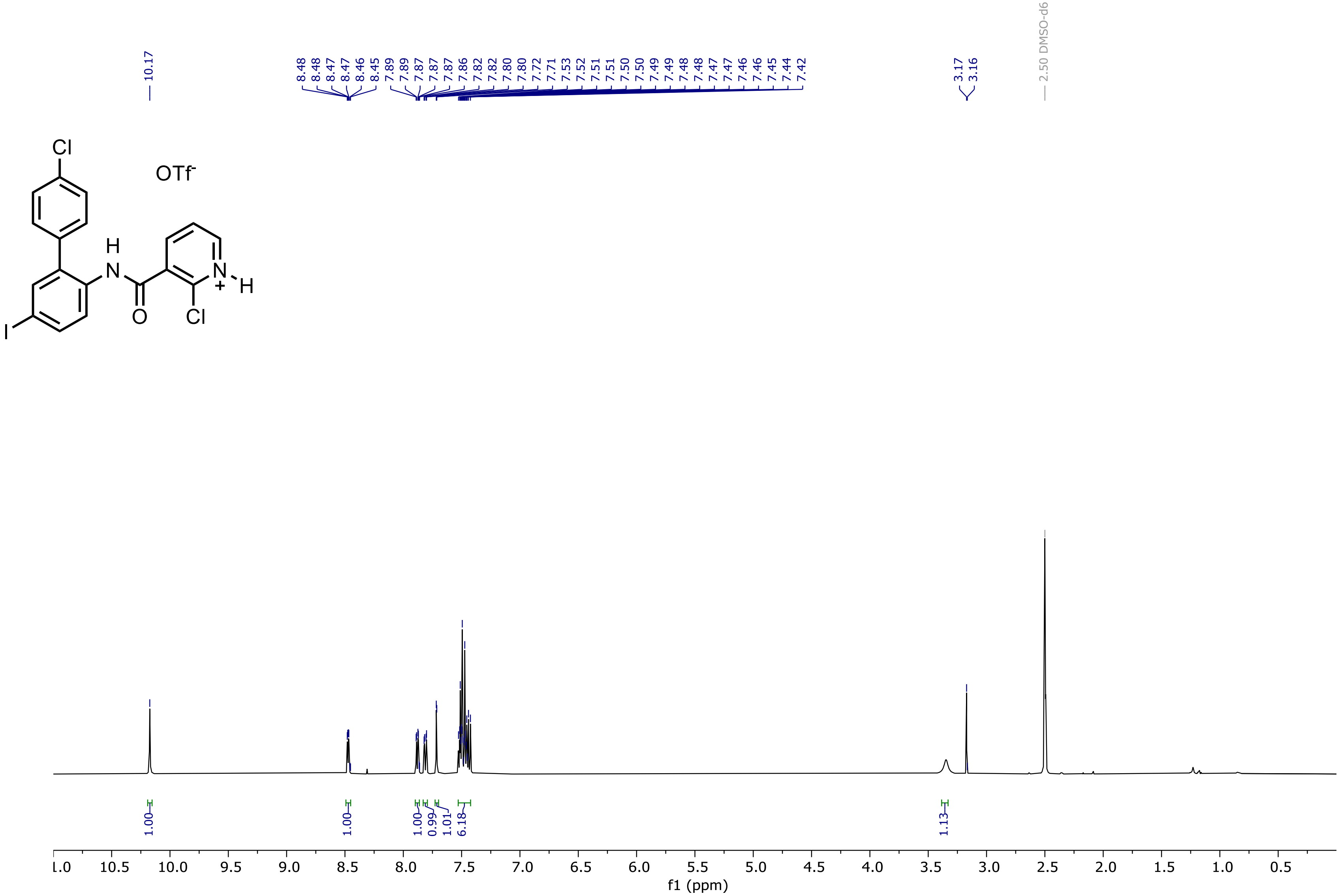

^{13}C NMR of Iodinated-boscalid triflate salt (21)

$(CD_3)_2SO$, 151 MHz, 25 ºC

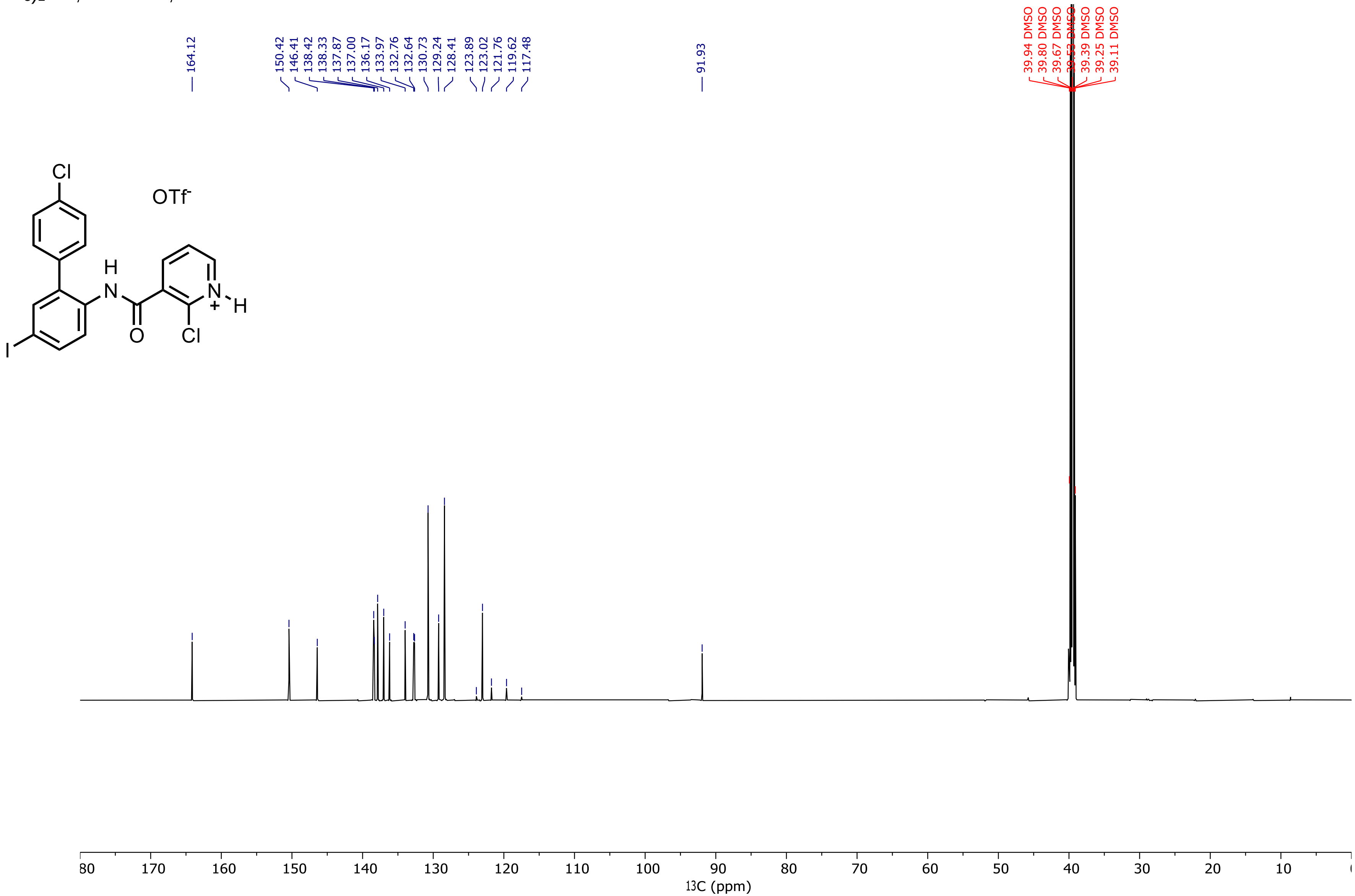

^{19}F NMR of Iodinated-boscalid triflate salt (21)

$(CD_3)_2SO$, 471 MHz, 25 ºC

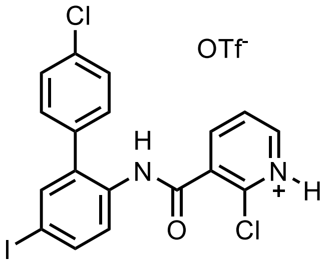

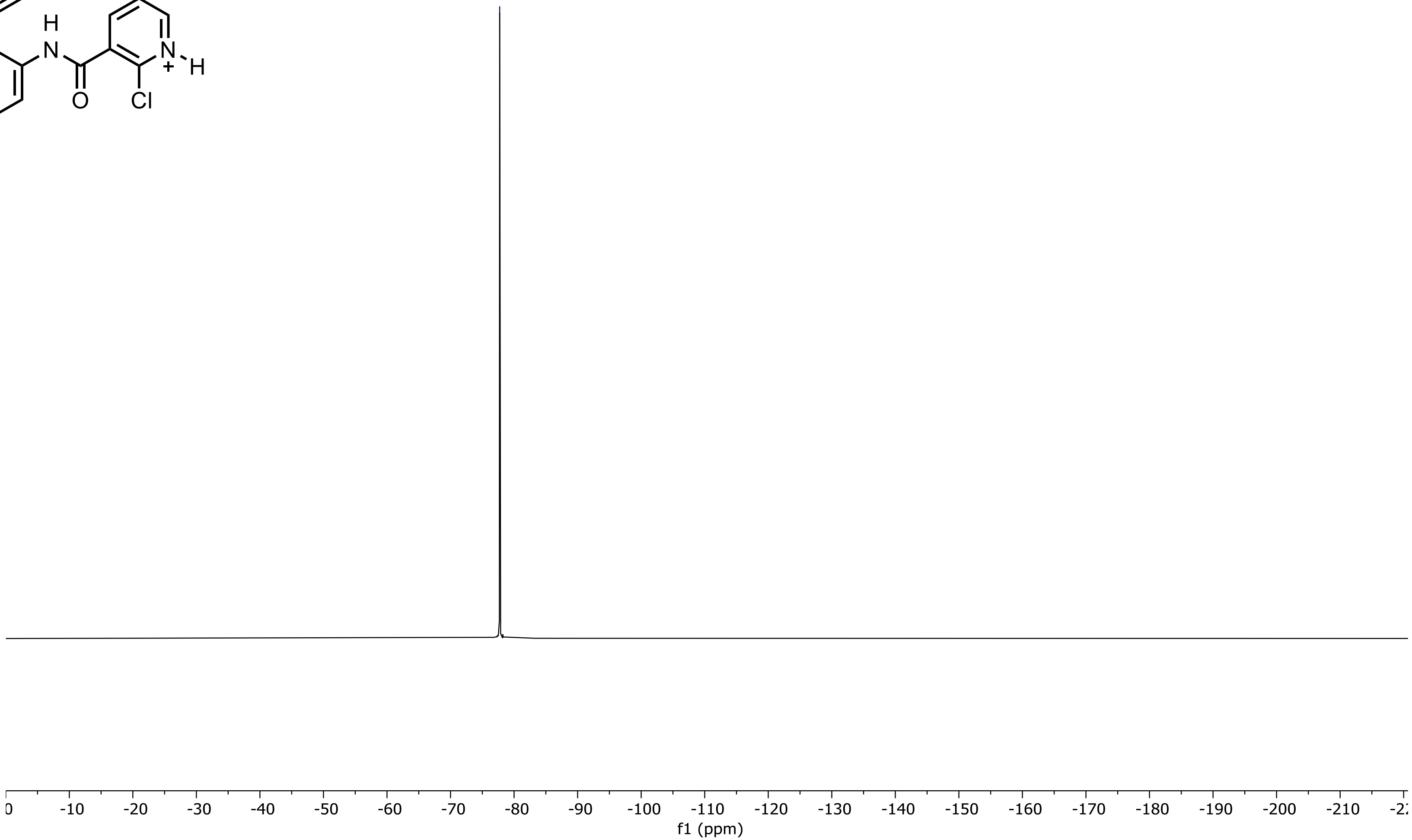

^{1}H NMR of Iodinated-diclofenac (22)

$(CD_3)_2SO$, 300 MHz, 25 ºC

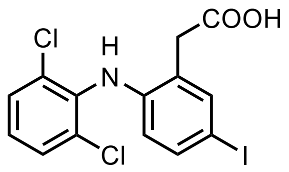

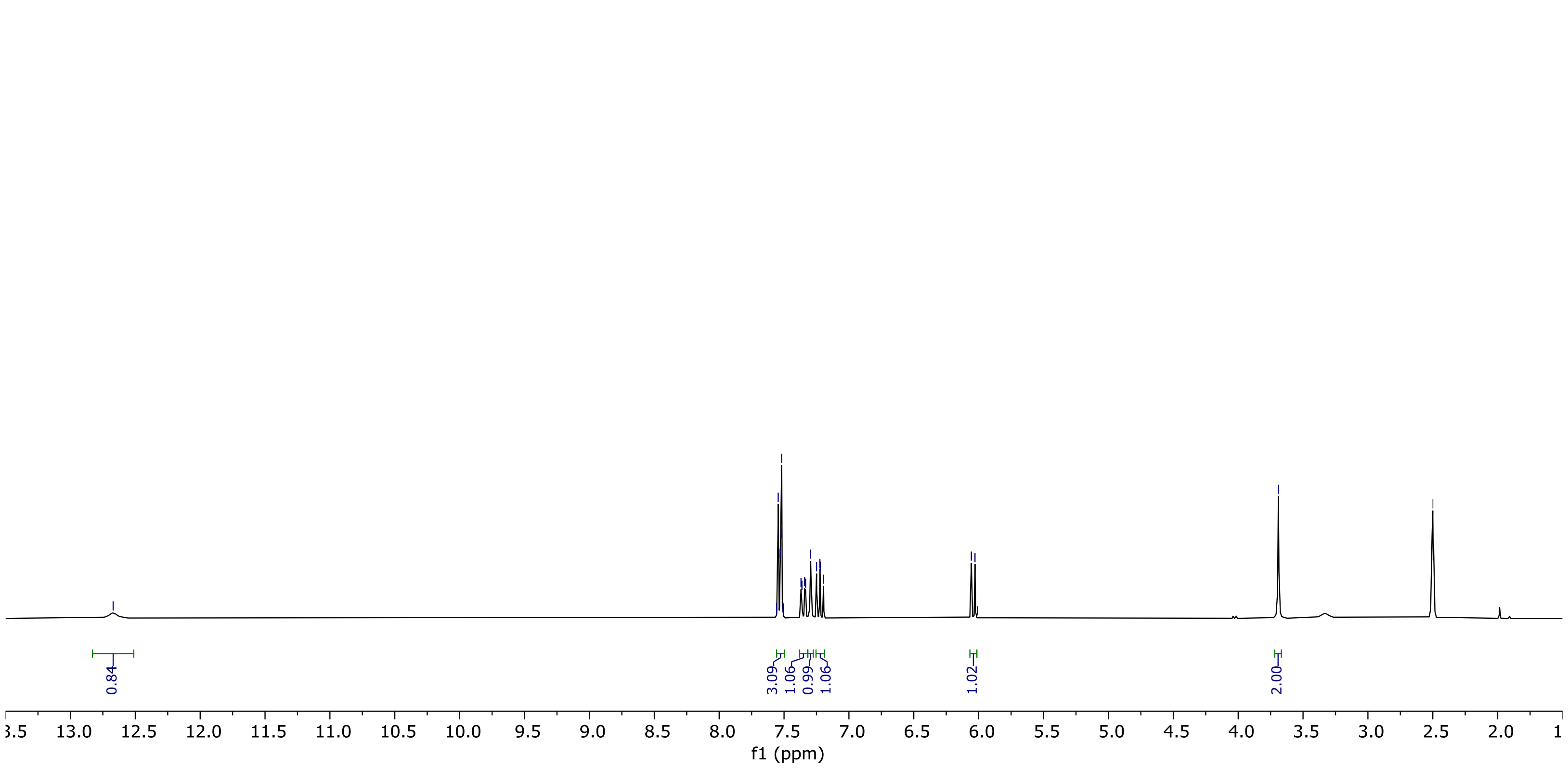

^{13}C NMR of Iodinated-diclofenac (22)

$(CD_3)_2SO$, 75 MHz, 25 ºC

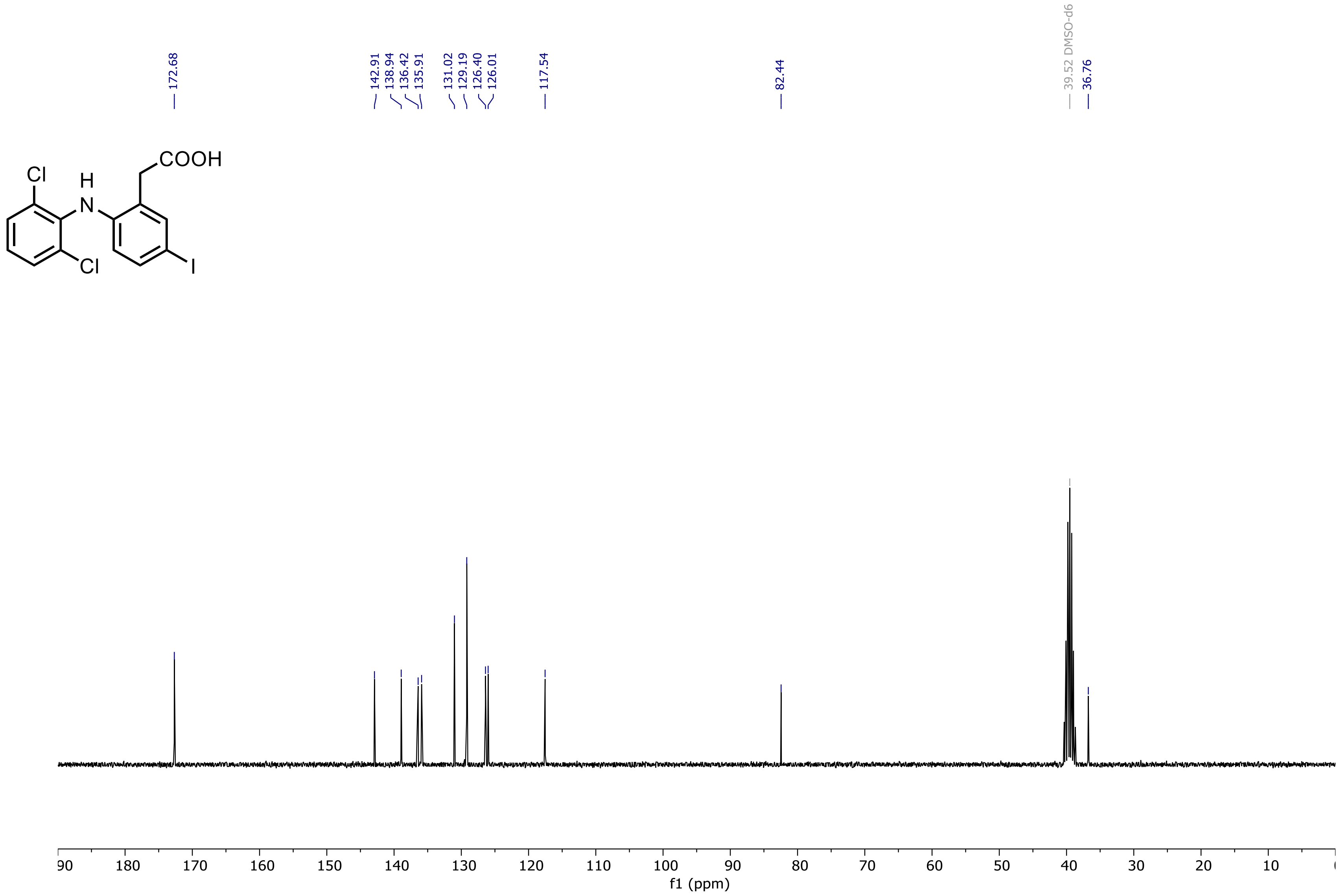

^{1}H NMR of Iodinated-napropamid (23)

CD_3CN, 500 MHz, 25 ºC

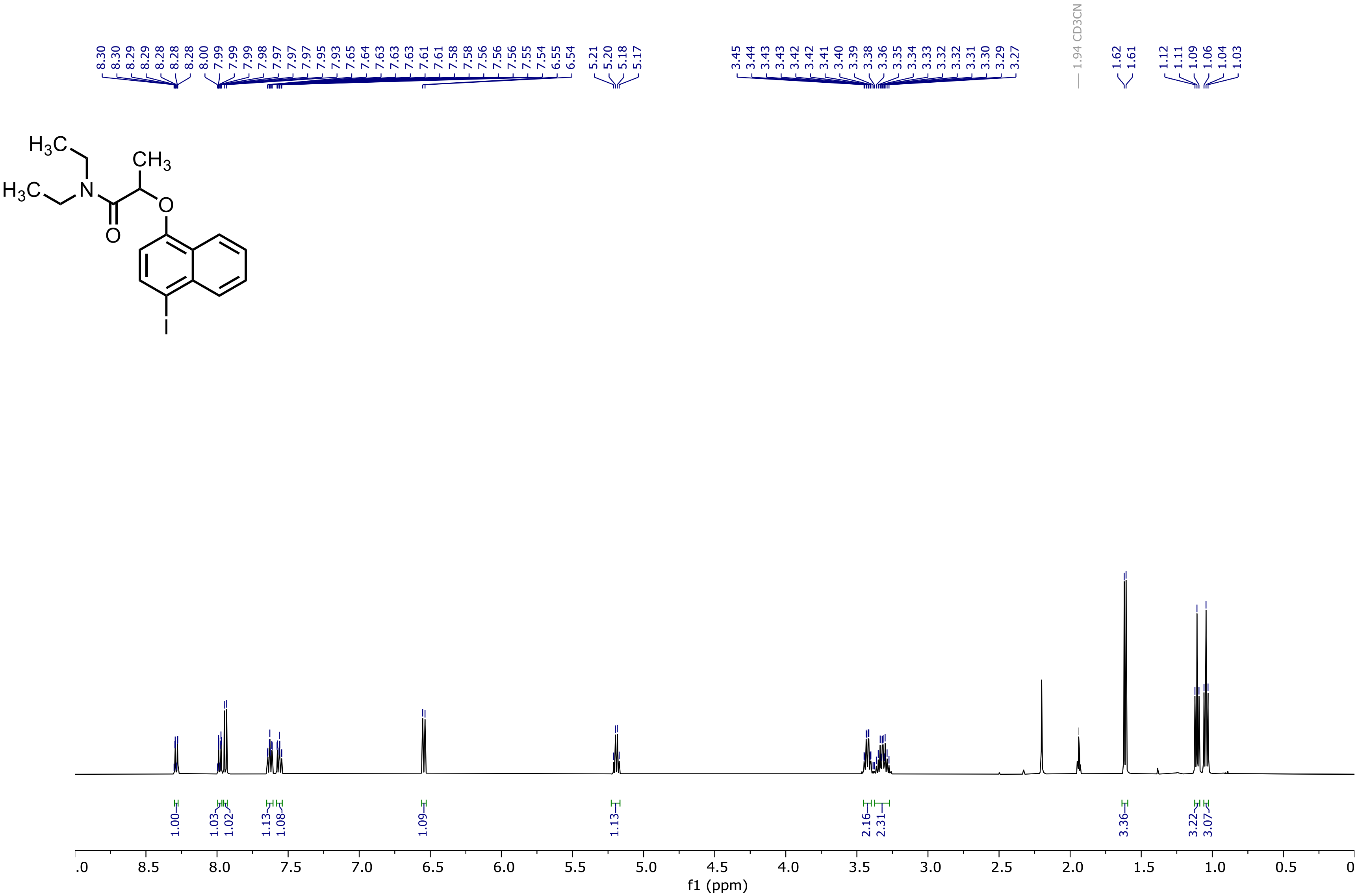

^{13}C NMR of Iodinated-napropamid (23)

CD_3CN, 126 MHz, 25 ºC

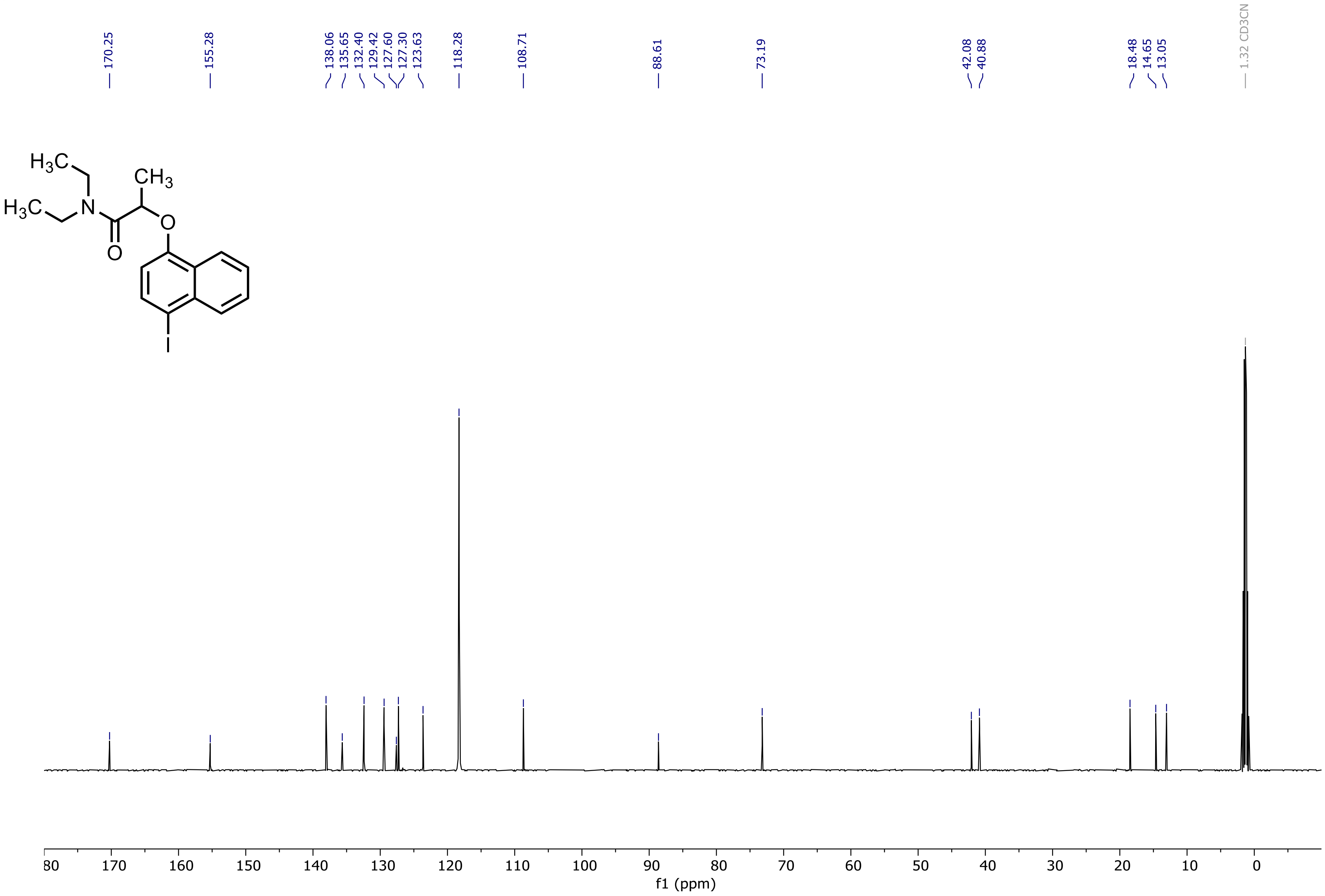

^{1}H NMR of Iodinated-fenobirinacid (24)

CD_2Cl_2, 500 MHz, 25 ºC

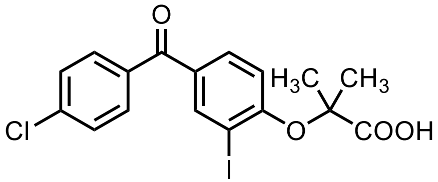

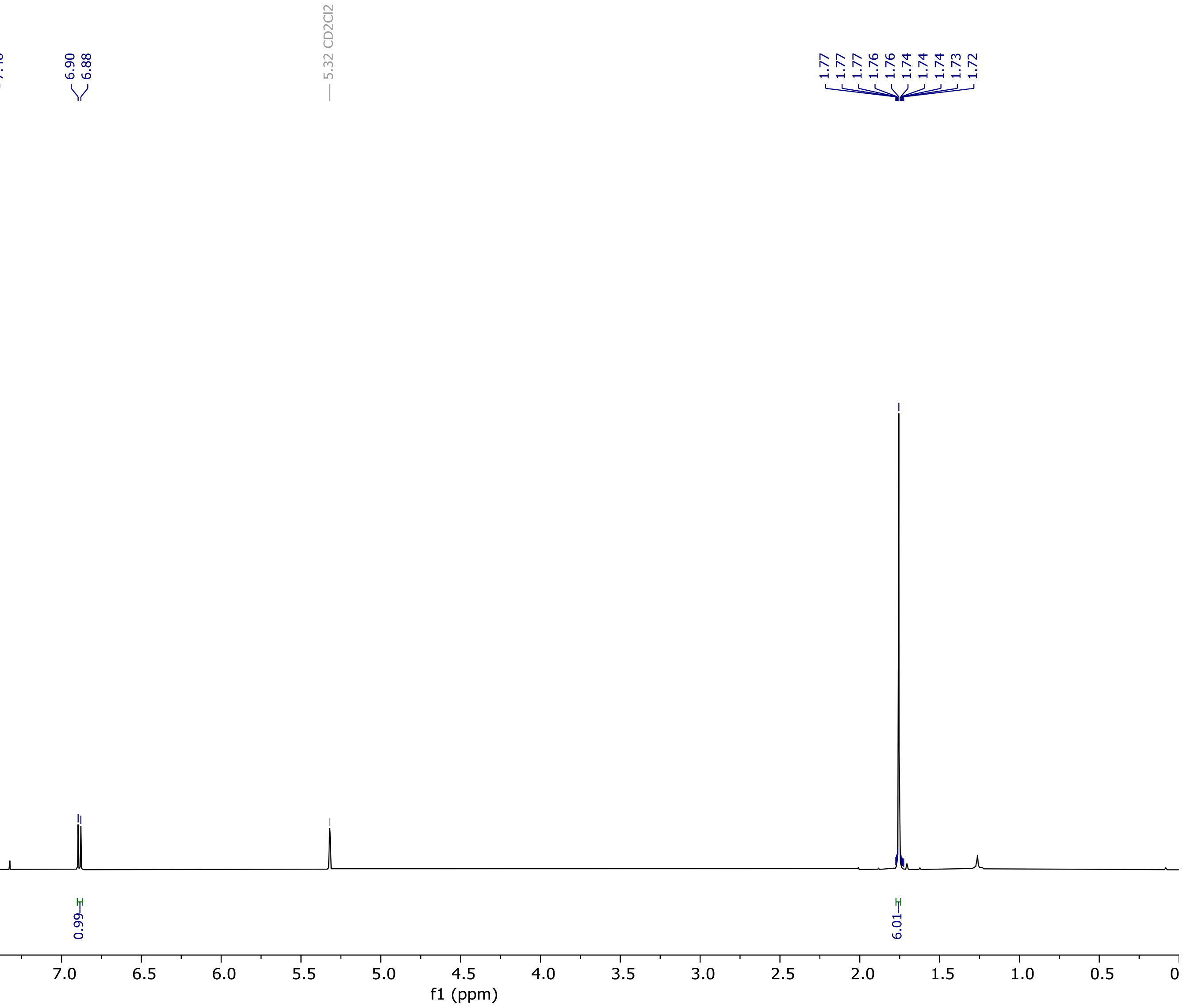

^{13}C NMR of Iodinated-fenobirinacid (24)

CD_2Cl_2, 126 MHz, 25 ºC

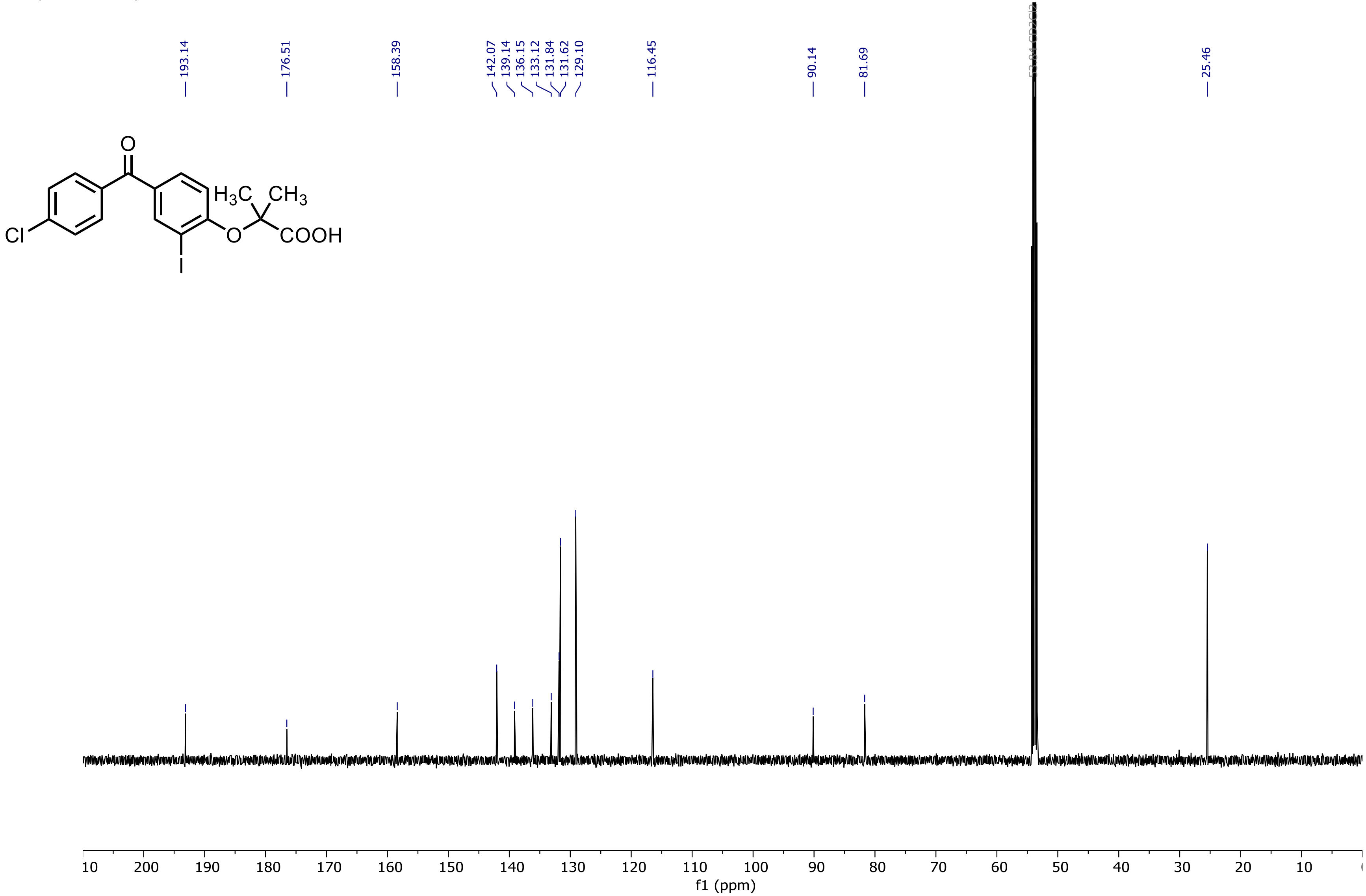

^{1}H NMR of Iodinated-strychnine triflate salt (25)

$(CD_3)_2SO$, 600 MHz, 25 ºC

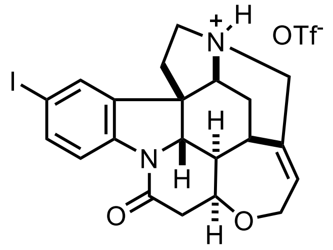

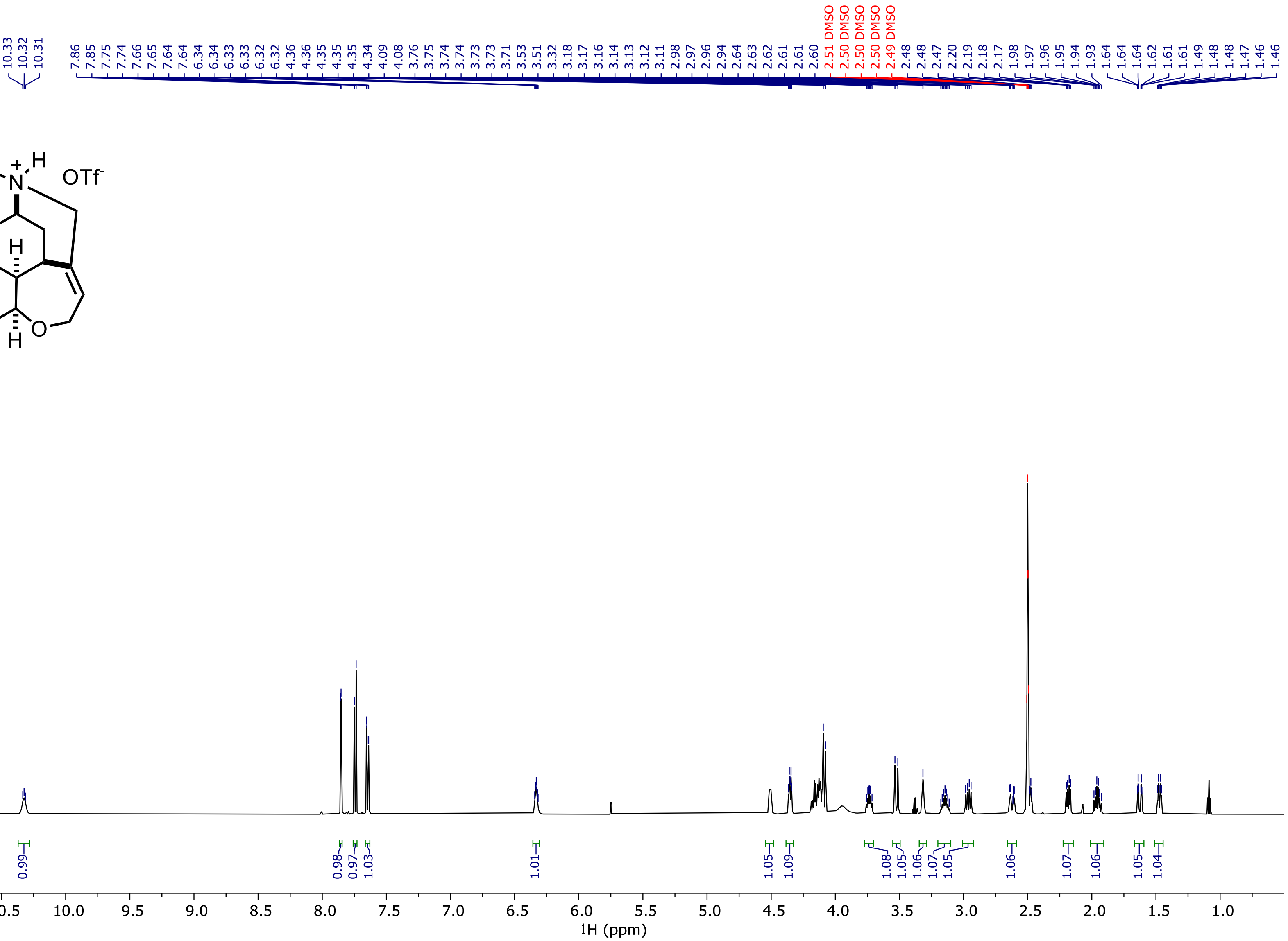

^{13}C NMR of Iodinated-strychnine triflate salt (25)

$(CD_3)_2SO$, 151 MHz, 25 ºC

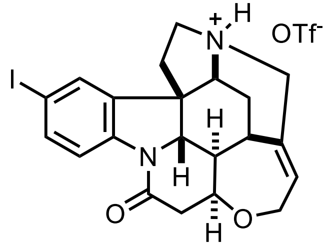

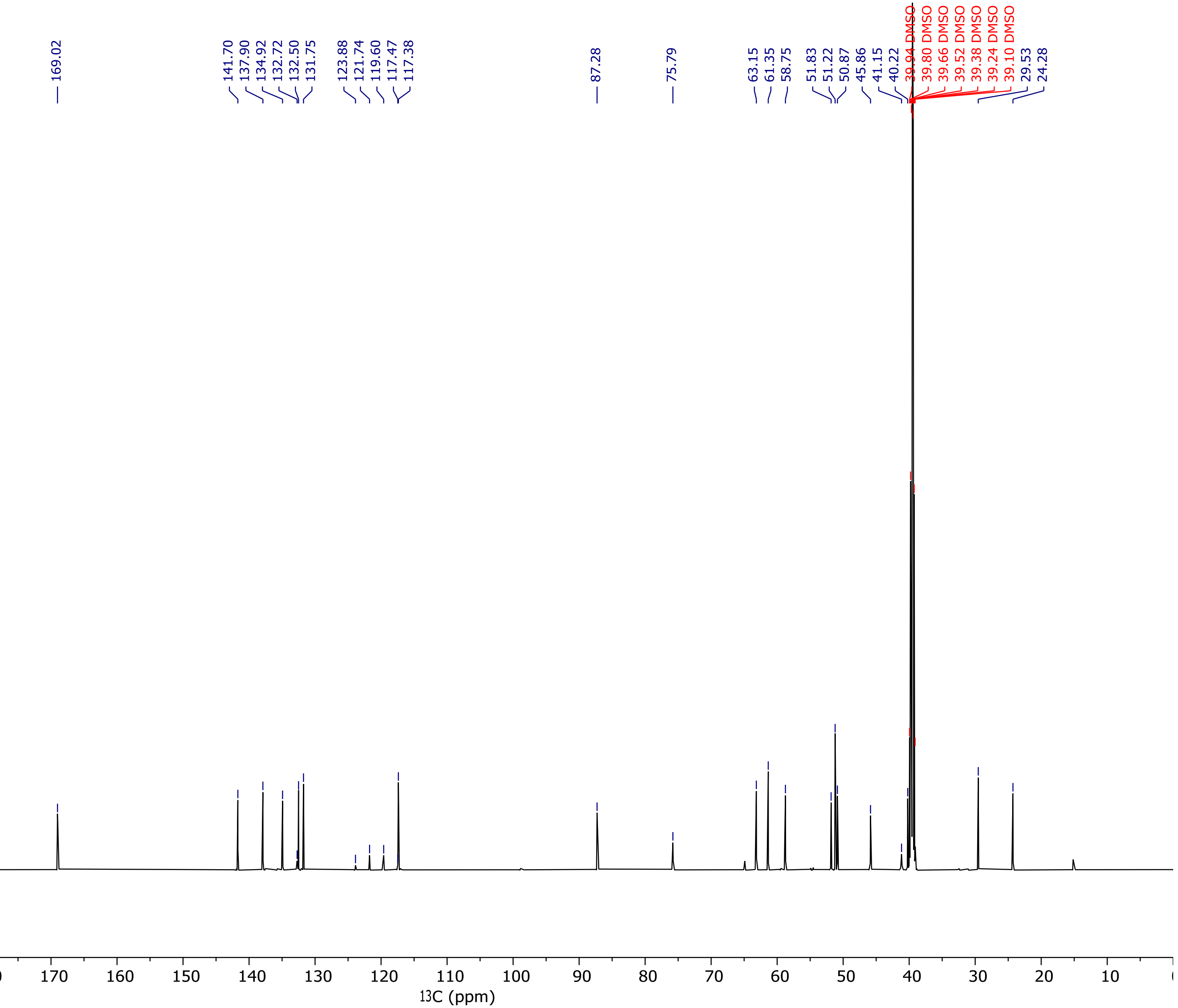

^{19}F NMR of Iodinated-strychnine triflate salt (25)

$(CD_3)_2SO$, 471 MHz, 25 ºC

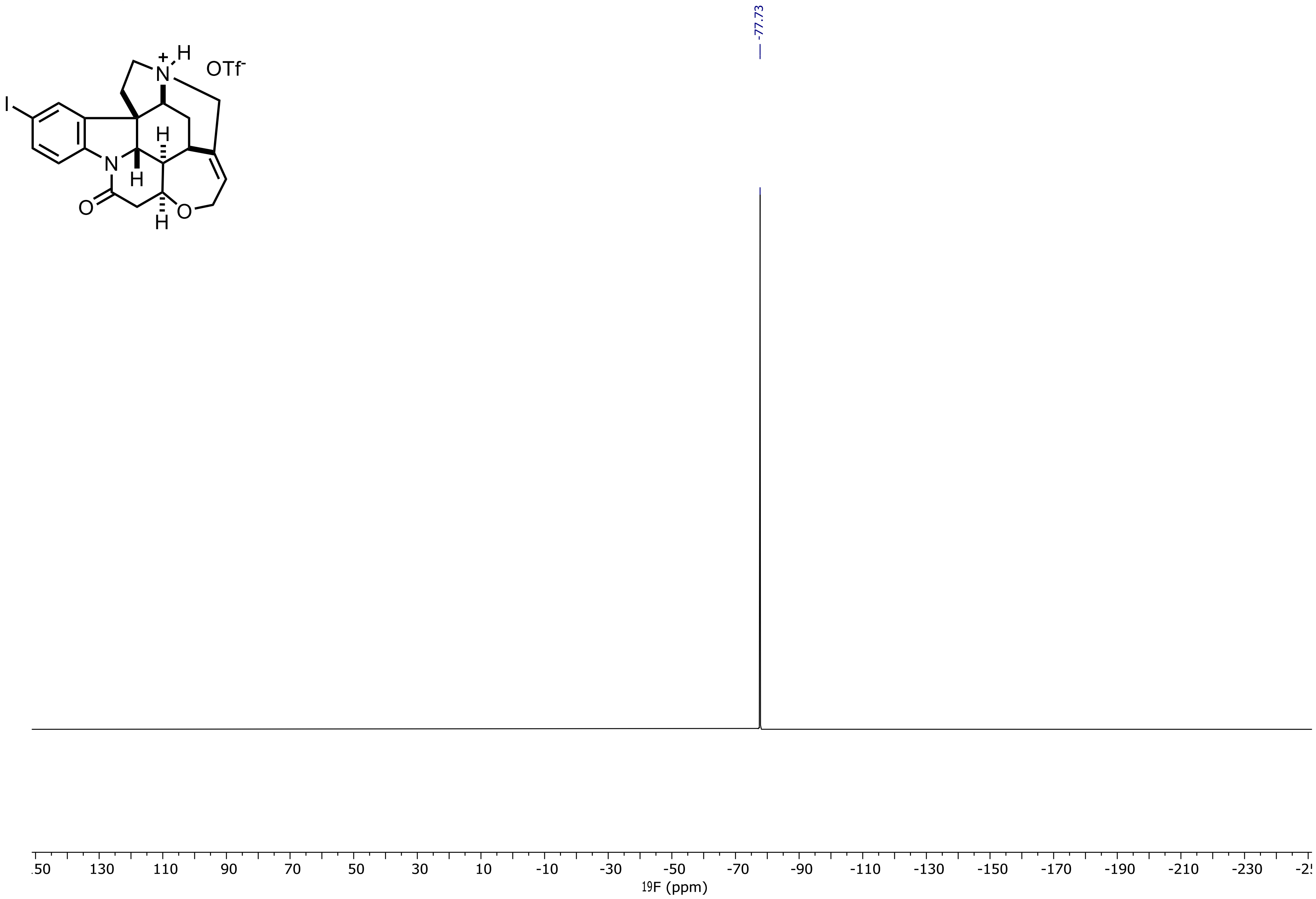

REFERENCES

1. Fulmer, G. R.; Miller, A. J. M.; Sherden, N. H.; Gottlieb, H. E.; Nudelman, A.; Stoltz, B. M.; Bercaw, J. E.; Goldberg, K. I. NMR Chemical Shifts of Trace Impurities: Common Laboratory Solvents, Organics, and Gases in Deuterated Solvents Relevant to the Organometallic Chemist. *Organometallics* **2010**, *29*, 2176–2179.
2. Guzmán Santiago, Alexis J.; Brown, Caleb A.; Sommer, Roger D.; and Ison, Elon A. Identification of key functionalization species in the Cp*Ir(III)-catalyzed- ortho halogenation of benzamides. *Dalton Trans* **2020**, *49*, 16166–16174.
